# New Monoterpene Glycoside Paeoniflorin Derivatives as NO and IL-1*β* Inhibitors: Synthesis and Biological Evaluation

**DOI:** 10.3390/molecules28196922

**Published:** 2023-10-03

**Authors:** Yongjie Chen, Guoqing Zhang, Dongyi Cao, Fei Wang, Fan Zhang, Huawu Shao, Wei Jiao

**Affiliations:** 1 Natural Products Research Centre, Chengdu Institute of Biology, Chinese Academy of Sciences, Chengdu 610041, China; 2 Nanchong Central Hospital, Nanchong 637000, China; 3 University of Chinese Academy of Sciences, Beijing 100049, China; 4 School of Pharmacy, North Sichuan Medical College, Nanchong 637100, China

**Keywords:** paeoniflorin, anti-inflammatory, nitric oxide, interleukin-1*β*

## Abstract

Several monoterpene glycoside compounds were extracted from *Paeonia lactiflora* Pall. Among them, paeoniflorin, a water-soluble monoterpene glycoside found in the root of *Paeonia lactiflora* Pall, exhibits excellent antioxidant pharmacological functions. Initially, Sc(CF_3_SO_3_)_3_ was employed as the catalyst for paeoniflorin’s dehydration and rearrangement reactions with alcohols. Subsequently, structural modifications were performed on paeoniflorin through a series of responses, including acetylation, deacetylation, and debenzoylation, ultimately yielding 46 monoterpene glycoside derivatives. The potential inhibitory effects on the pro-inflammatory mediators interleukin-1 beta (IL-1*β*) and nitric oxide (NO) were assessed in vitro. The results revealed that compounds **29** and **31** demonstrated notable inhibition of NO production, while eight derivatives (**3**, **8**, **18**, **20**, **21**, **29**, **34**, and **40**) displayed substantial inhibitory effects on the secretion of IL-1*β*. Computational research was also undertaken to investigate the binding affinity of the ligands with the target proteins. Interactions between the proteins and substrates were elucidated, and corresponding binding energies were calculated accordingly. The findings of this study could provide valuable insights into the design and development of novel anti-inflammatory agents with enhanced pharmacological properties.

## 1. Introduction

Inflammation is a multifaceted biological response pivotal in the body’s defense mechanisms against diverse pathogens and injuries [1]. Nevertheless, the excessive and persistent inflammatory reaction may lead to various chronic diseases, including rheumatoid arthritis [2], atherosclerosis [3,4], and cancer [5]. Among the key mediators of the inflammatory response are nitric oxide (NO) and interleukin-1 beta (IL-1*β*) [6,7,8,9]. Their excessive production has been associated with the pathogenesis of several inflammatory diseases. Consequently, developing novel agents capable of modulating the NO and IL-1*β* signaling pathways has garnered significant therapeutic interest.

**Figure 1:**
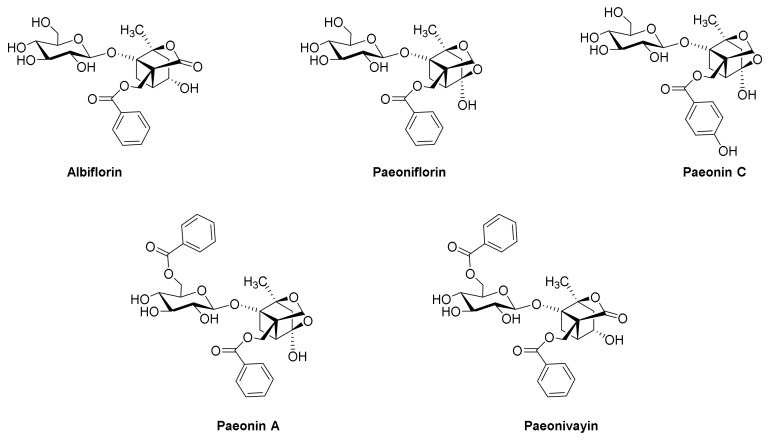
Several monoterpene glycoside compounds.

*Paeonia lactiflora* Pall, a traditional Chinese medicine renowned for its anti-inflammatory and immune-regulatory properties [10,11], has demonstrated remarkable efficacy in the treatment of various inflammatory ailments, including rheumatoid arthritis, hepatic fibrosis, and colitis [12,13]. The dried roots of *P. lactiflora* encompass a spectrum of potent constituents collectively referred to as total glucosides of paeony (TGP). Within the TGP, several compounds are comprised, such as albiflorin, paeonin C, paeonin A, paeonivayin, lactiflorin, and, notably, paeoniflorin (Figure 1) [14]. Paeoniflorin stands out as the preeminent component of TGP, constituting more than 40% of its composition. Meanwhile, paeoniflorin boasts a unique structural characteristic [15], classifying it as a cage-like monoterpenoid glycoside with a pinane skeleton [16]. Its significance is underlined by its diverse pharmacological effects, encompassing anti-inflammatory [11], analgesic [17], immunomodulatory [18], and neuroprotective activities [19]. In the context of paeoniflorin’s physicochemical properties, it exhibits solid hydrophilic properties and limited transmembrane absorption due to its weak lipophilicity [20]. These attributes substantially impede its ability to cross membranes [21,22,23,24]. Pharmaceutical chemists have endeavored to enhance its biological activity by structural modifications. For instance, removing paeoniflorin’s hydrophilic pyranose structure increases its ability to penetrate Caco-2 cells by 48 times [25]. In vitro investigations have also indicated that acetylation augments paeoniflorin’s absorption and lipophilicity of paeoniflorin, subsequently enhancing the bioavailability of benzoyl paeoniflorin sulfonate in mouse models [26].

**Scheme 1:**
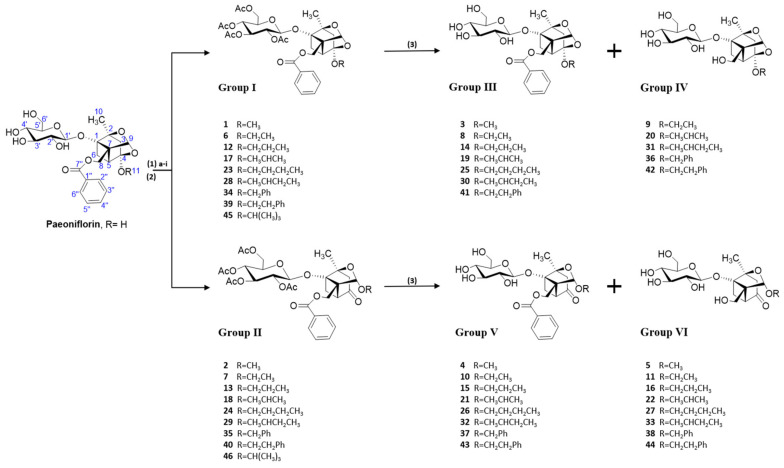
The synthetic routes to compounds **1–46**. Reagents and conditions: (1) a: CH_3_OH, Sc(CF_3_SO_3_)_3_, 65 °C, 30 min; b: EtOH, Sc(CF_3_SO_3_)_3_, 78 °C, 45 min; c: *n*-propanol, Sc(CF_3_SO_3_)_3_, 97 °C, 40 min; d: isopropyl alcohol, Sc(CF_3_SO_3_)_3_, 82.5 °C, 60 min; e: BuOH, Sc(CF_3_SO_3_)_3_, 117.7 °C, 45 min, f: 2-butyl alcohol, Sc(CF_3_SO_3_)_3_, 99.5 °C, 35 min; g: *tert*-butanol, Sc(CF_3_SO_3_)_3_, 82.4 °C, 10 h; h: THF/benzyl alcohol (1:1), Sc(CF_3_SO_3_)_3_, 66 °C, 45 min; i: THF/phenylethyl alcohol (1:1), Sc(CF_3_SO_3_)_3_, 66 °C, 1 h. (2) Acetic anhydride/pyridine (1:1), 0 °C, 1 h. (3) CH_3_OH, Et_3_N, rt, 24 h.

Furthermore, the chemical transformations of paeoniflorin are challenging, mainly because of its complex and unique structure and the limited understanding of its reactivity [27,28]. A mixture of 4-*O*-methyl(ethyl) and 4-oxo-9-*O*-methyl(ethyl) ethers was obtained when paeoniflorin was reacted with lower alcohols in the presence of cation exchanger KU-2-8 (H^+^) [29] or *p*-toluene sulfonic acid [30]. Herein, six-group derivatives (Groups I–VI) were designed and synthesized by modifying the C-4 groups [31,32], benzoyl, and glucose groups of paeoniflorin, as illustrated in Scheme 1. The obtained derivatives were subjected to cytotoxicity, inflammasome activation, and NO production by macrophages. Additionally, computational studies were conducted to elucidate the binding modes of these derivatives with the designated target proteins.

## 2. Results and Discussion

### 2.1. Chemistry

**Figure 2:**
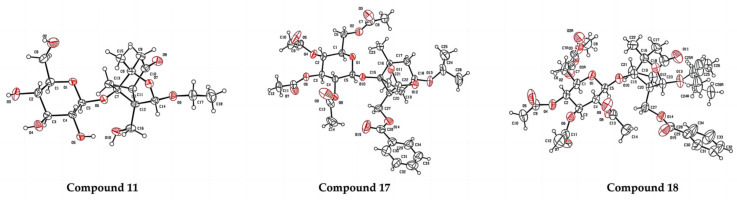
Crystal structures of compounds **11**, **17**, and **18**.

Building upon our prior investigation involving the extraction of paeoniflorin [33,34]. Initially, paeoniflorin, albiflorin, paeonin C, paeonivayin, and paeonin A were extracted from the dried roots of *P. lactiflora*. Subsequently, after meticulous evaluation, the Lewis acid Sc(CF_3_SO_3_)_3_ was determined as the optimal catalyst for paeoniflorin’s dehydration and rearrangement reactions with alcohols. This catalyst was instrumental in facilitating the intermolecular dehydration of alcohols and the hydroxyl group at the C-4 position of paeoniflorin through a ring-opening rearrangement of the acetal reaction. This cascade led to the formation of two distinct isomers, which then underwent acetylation (Groups I and II), deacetylation (Groups III and IV), and debenzoylation reactions to yield compounds of high purity (Groups V and VI). In total, paeoniflorin’s 46 derivatives were synthesized utilizing the aforementioned reaction conditions. To ensure the unequivocal identification of these derivatives, nuclear magnetic resonance spectroscopy, and mass spectrometry were employed for structural characterization. Furthermore, the X-ray structures of representative compounds are presented in Figure 2.

### 2.2. Biological Evaluation

#### 2.2.1. Cytotoxic Effects of Paeoniflorin Derivatives on Macrophages

Prior to delving into the anti-inflammatory aspects, it is imperative to assess the impact of both monoterpene glycoside compounds and paeoniflorin derivatives on cell viability. J774A.1 cells and RAW264.7 cells were separately incubated with a concentration of 5 and 10 μmol/L for 24 h. The ensuing cytotoxic effects were gauged by employing the MTT assay.

**Figure 3:**
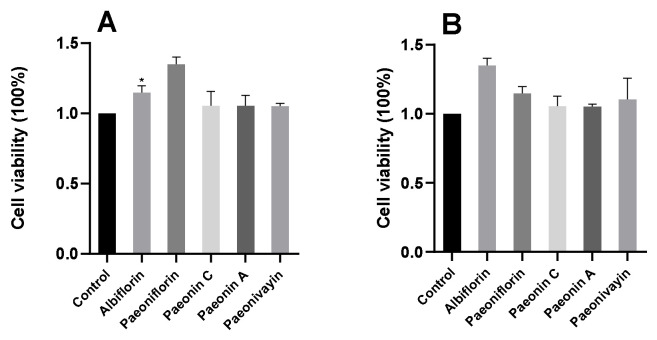
Viability of macrophage cells treated with monoterpene glycoside compounds. (**A**) Viability of J774A.1 cells treated with monoterpene glycoside compounds. (**B**) Viability of RAW264.7 cells treated with monoterpene glycoside compounds. Values are expressed as the mean ± standard deviation, and significant differences are represented with * (*p* < 0.05).

**Figure 4:**
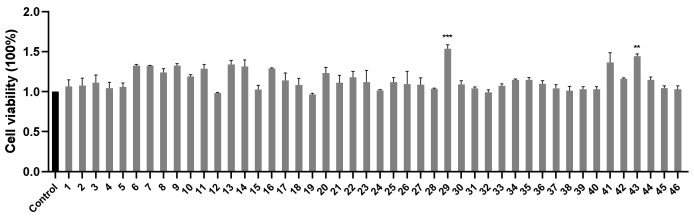
Viability of J774A.1 cells following treatment with paeoniflorin derivatives. Values are expressed as the mean ± standard deviation, and significant differences are denoted as follows: ** *p* < 0.01; *** *p* < 0.001.

**Figure 5:**
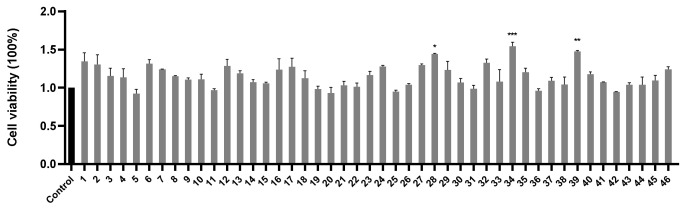
Viability of RAW264.7 cells following treatment with paeoniflorin derivatives. Values are expressed as the mean ± standard deviation, and significant differences are denoted as follows: * *p* < 0.05; ** *p* < 0.01; *** *p* < 0.001.

The results showed that albiflorin, paeonin C, paeonin A, paeonivayin, and paeoniflorin have no inhibitory effect on the proliferation of J774A.1 cells and RAW264.7 cells (Figure 3). In comparison to the control group, the paeoniflorin derivatives also exhibited no discernible suppressive effects on the proliferation of J774A.1 cells (Figure 4) and RAW264.7 cells (Figure 5). Based on these outcomes, the changes observed in subsequent experiments arguably were not caused by the cytotoxicity. Therefore, compounds at a concentration of 5 and 10 μmol/L were used for further anti-inflammatory experimentation.

#### 2.2.2. IL-1β Secretion following NLRP3 Inflammasome Activation

The NLRP3 inflammasome has been implicated in various inflammatory diseases, such as type 2 diabetes [35,36], gout [37,38], and atherosclerosis [39,40]. Activation of NLRP3 inflammatory corpuscles in macrophages would produce IL-1*β* [41,42,43]. As a key step of inflammatory reaction [44], IL-1*β* is elevated in various neuropathic pain conditions [45], which recruit innate and adaptive components of inflammation to sites of injury [46]. Therefore, the inhibition of the secretion of IL-1*β* from NLRP3 inflammasomes is always measured to evaluate anti-inflammatory activity.

In this study, to investigate the effects of monoterpene glycoside compounds and paeoniflorin derivatives on NLRP3 inflammation, IL-1*β* release was evaluated using ELISA and western blot analysis. J774A.1 cells were primed with LPS and stimulated with Nigericin. The inhibitory potential of paeoniflorin and its derivatives was evaluated by pretreatment of the cells with concentrations of 5 μmol/L of compounds for 30 min prior to incubation with inflammasome stimuli to observe the effect of compounds on IL-1*β* secretion caused by the activation of NLRP3 inflammatory corpuscle.

**Figure 6:**
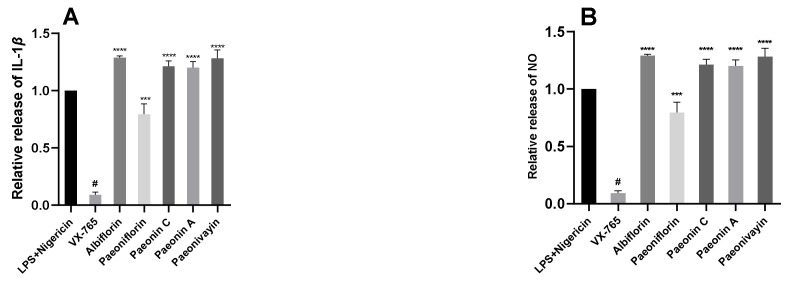

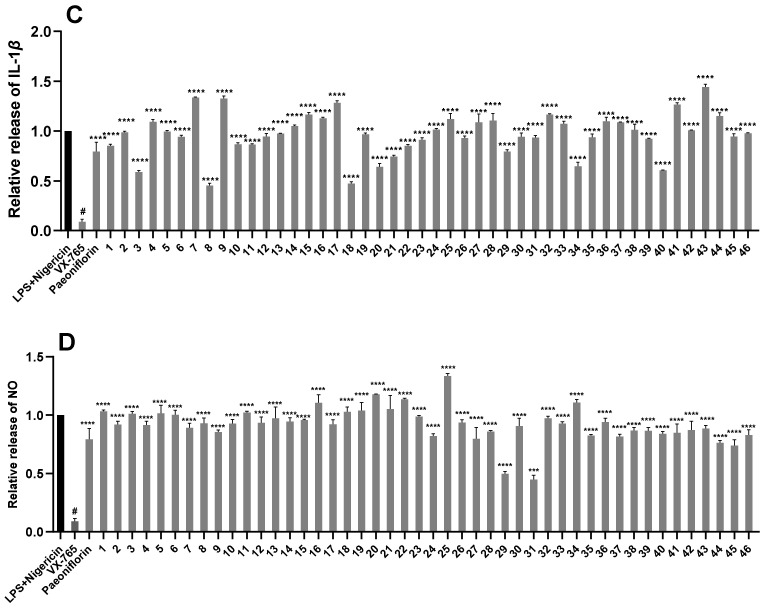
Anti-inflammatory studies in vitro models. (**A**,**C**) IL-1*β* secretion following NLRP3 inflammasome activation. (**B**,**D**) The relative release of NO on LPS-induced RAW264.7 macrophages. Values are expressed as the mean ± standard deviation. Statistical significance relative to the LPS group was indicated, # *p* < 0.05. Statistical significance relative to the VX-765 group was indicated, *** (*p* < 0.001) and **** (*p* < 0.0001).

The results showed that albiflorin, paeonin C, paeonin A, and paeonivayin had fewer inhibitory effects than paeoniflorin (Figure 6A). Therefore, further studies on the inhibitory effect on IL-1*β* secretion of paeoniflorin derivatives were carried out. The statistics proved that compounds **8** and **18** had a more obvious inhibitory effect on IL-1*β* secretion compared with other paeoniflorin derivatives. Compounds **3**, **20**, **21**, **29**, **34**, and **40** had slightly better inhibitory effects. Other compounds had fewer inhibitory effects than paeoniflorin. The results are shown in Figure 6C. These data suggested that inhibition of NLRP3 inflammasome activation might be involved in the reduction in IL-1*β* release by paeoniflorin’s derivatives.

Substitution of paeoniflorin with a methyl ether and an ethyl ether at C-4 (compounds **3** and **8**) leads to better inhibitory activity compared to paeoniflorin. Branched-chain alcohols (compounds **18**, **20**, **21**, and **29**) exhibit better IL-1*β* inhibition activity than straight-chain alcohols. Cetyl protection does not enhance the inhibitory activity of alkylated paeoniflorin (compounds **1**, **6**, **11**, **22,** etc.). However, when paeoniflorin is substituted at C-4 with an ether containing a phenyl group, the presence of acetyl protection can enhance its inhibitory activity (compounds **34** and **40**).

#### 2.2.3. Inhibition of NO Production on LPS-Induced RAW264.7 Macrophage Cells

NO is an important bioactive gas and effector molecule that is widely involved in physiological and pathological processes in immunology [47]. The high amount of NO is one of the causes that lead to tissue damage in many inflammatory disorders, such as rheumatoid arthritis, where a large amount of NO is liberated from the macrophages [48]. When stimulated and activated, macrophages release cytotoxic NO, which induces an inflammatory response [49]. Inhibition of NO production is an important pathway to treat various inflammatory diseases [50]. Therefore, Inhibition of NO production can be selected as an important indicator to evaluate anti-inflammatory activity.

Hence, the target compounds were tested for their ability to decrease NO concentration in LPS-activated RAW 264.7 macrophages. The macrophages were treated with the target compounds and LPS (10 µmol/L) for 24 h. The NO production was indirectly analyzed by measuring the sodium nitrite concentration based on the Griess reaction, and the effects of compounds on the NO production induced by activation of RAW264.7 macrophages were observed. The results showed that several TGP compounds had similar inhibitory activity (Figure 6B). The inhibitory effect of compounds **29** and **31** on the production of NO was superior to that of paeoniflorin. Other compounds had fewer inhibitory effects than paeoniflorin (Figure 6D).

Compounds **29** and **31** are glucose-deacetylated and isomers derived from the isobutyl group, which indicates that isobutyl may be the dominant group for inhibiting the production of NO. However, other alcohols could not improve the inhibitory activity of paeoniflorin. In addition, the protection of the acetyl group against the glycyclic ring could not improve the inhibitory activity of paeoniflorin.

#### 2.2.4. Docking Analysis

A molecular docking study of paeoniflorin or its derivatives was performed based on the model of the IL-1*β* (PDB ID: 5R8Q) and iNOS (PDB ID: 3HR4) to understand the binding mode of compounds at the active sites. The molecular docking study was carried out using the Glide software (Grid-Based Ligand Docking with Energetics) module incorporated in the Schrodinger molecular modeling package (Schrodinger, version 13.5, LLC, New York, NY, USA). The glide SP mode was used for the docking of the compounds.

Paeoniflorin and its derivatives (**8**, **18**, **29,** and **31)** were chosen for molecular docking and compared with a natural substrate. Each of the compounds’ docked positions was evaluated, and the pose with the lowest binding free energy was selected. The anticipated binding free energies (kcal/mol) were used to determine the molecular docking scores. The best dock score shows the strongest ligand-protein affinity, which has the lowest binding free energy (Table 1).

**Figure 7:**
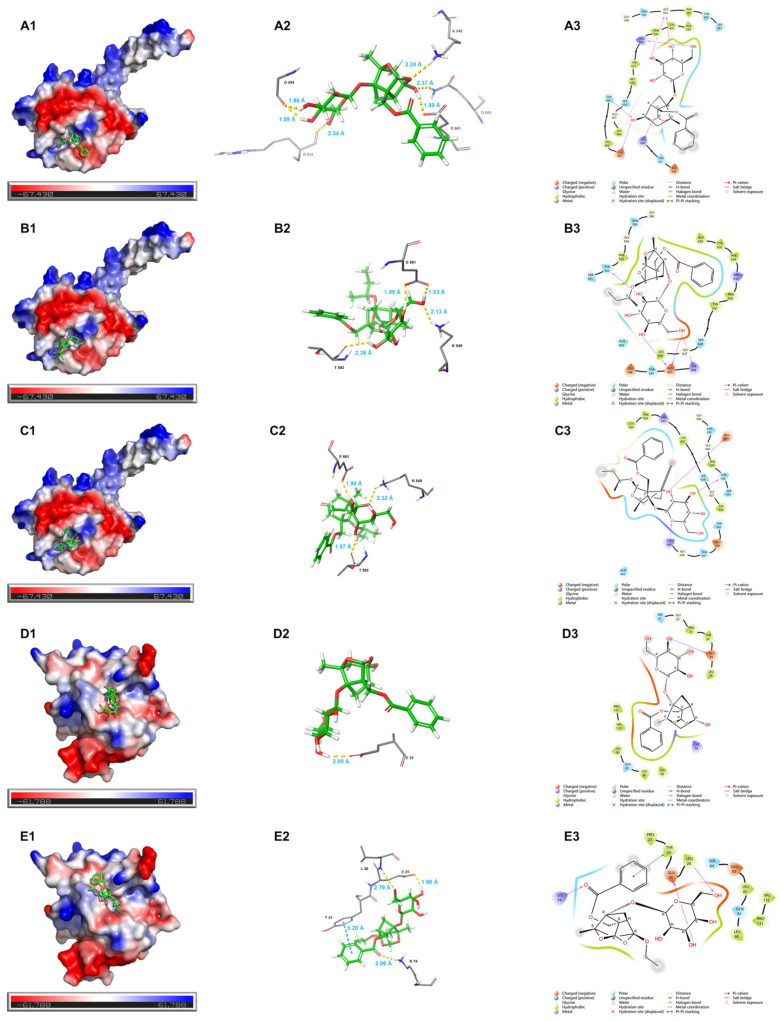

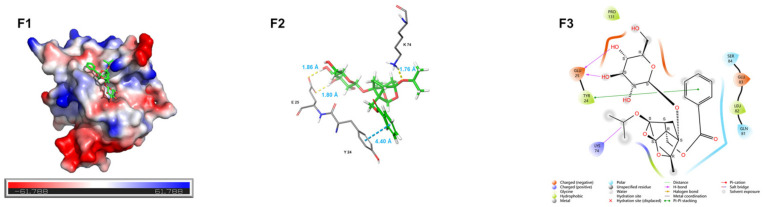
Docking results of protein (PDB code: 3HR4 and 5R8Q) binding with ligands. (**A1**) The electrostatic potential of docking complex of paeoniflorin against iNOS. (**A2**) 3D docking poses of paeoniflorin with amino acid residues. (**A3**) 2D interaction plot showing the interactions of paeoniflorin and amino acid residues. (**B1**) The electrostatic potential of docking complex of compound **29** against iNOS. (**B2**) 3D docking poses of compound **29** with amino acid residues. (**B3**) 2D interaction plot showing the interactions of compound **29** and amino acid residues. (**C1**) The electrostatic potential of docking complex of compound **31** against iNOS. (**C2**) 3D docking poses of compound **31** with amino acid residues. (**C3**) 2D interaction plot showing the interactions of compound **31** and amino acid residues. (**D1**) The electrostatic potential of docking complex of paeoniflorin against IL-1*β*. (**D2**) 3D docking poses of paeoniflorin with amino acid residues. (**D3**) 2D interaction plot showing the interactions of paeoniflorin and amino acid residues. (**E1**) The electrostatic potential of docking complex of compound **8** against IL-1*β*. (**E2**) 3D docking poses of compound **8** with amino acid residues. (**E3**) 2D interaction plot showing the interactions of compound **8** and amino acid residues. (**F1**) The electrostatic potential of docking complex of compound **18** against IL-1*β*. (**F2**) 3D docking poses of compound **18** with amino acid residues. (**F3**) 2D interaction plot showing the interactions of compound **18** and amino acid residues.

Both the substrate and the protein contain partial charges, and these charges have a considerable influence on how rapidly they connect to one another. The protein electrostatic potential diagram may be used to gain insight into the 3D architecture and topology of substrates. Each value in a protein electrostatic potential diagram represents a distinct color, from blue to red; its positive (blue) and negative (red) regions are linked to nucleophilic and electrophilic reactivity, respectively (Figure 7A1–F1).

The docking studies of compounds **29** and **31** demonstrated a stronger binding affinity to IL-1*β* when compared to paeoniflorin (−5.74 kcal/mol), which had binding energies of -6.24 and 7.49 kcal/mol with IL-1*β*, respectively. Compounds **29** and **31** established hydrogen bonds with amino acid residues GLU661, LYS549, and THR592 of IL-1*β*, as illustrated in Figure 7 and Table 1. These docking studies suggest that compounds **29** and **31** exhibit greater affinity and potency than paeoniflorin.

On the other hand, the binding free energy values for compounds **8** and **18** were −9.15 kcal/mol and −9.54 kcal/mol, respectively, superior to that of paeoniflorin (−5.58 kcal/mol). Compound **8** formed hydrogen bonds with amino acid residues GLU25, LEU26, and LYS74 of iNOS at cumulative hydrogen bond distances of 1.96 Å, 2.79 Å, and 2.06 Å, and pi-pi stacking with the active site residue TYR24 (5.20 Å) of iNOS. Similarly, in addition to forming pi-pi stacking with TYR24 (5.20 Å), compound **18** also formed hydrogen bonds with GLU25 and LYS74. In contrast, paeoniflorin only formed one hydrogen bond with the active site residue GlU25. These results suggest that the synthesized molecules have a higher dock score and more stable conformation with iNOS than paeoniflorin, indicating greater target-ligand affinity when compared to paeoniflorin, as shown in Figure 7 and Table 1.

## 3. Materials and Methods

### 3.1. Cell Culture

J774A.1 and RAW264.7 cell lines were purchased from ProteinTech Group Inc. (Wuhan, China) and examined for mycoplasma contamination by short tandem repeat profiling. Both J774A.1 cells and RAW264.7 cells were cultured in DMEM complete medium with high glucose containing 10% fetal bovine serum (FBS), 100 U/mL penicillin, and 100 U/mL streptomycin in a humidified 5% CO_2_ incubator at 37 °C.

### 3.2. Cell Viability Assay

Cell viability was measured using the MTT assay. Cells at a density of 2 × 104 cells per well were plated in 24-well culture plates. After 24 h, the medium was removed, and 500 μL fresh medium containing 0.1% DMSO or different concentrations of the test compounds, as indicated, were added to the 24-well culture plates and incubated for 48 h. MTT was added at a final concentration of 1 mg/mL and incubated for 3 h. The absorbance was determined at 570 nm, and the viability (%) was calculated. For each compound, the experiment was conducted in triplicate.

### 3.3. NLRP3 Inflammasome Corpuscle Stimulation

The supernatant of J774A.1 cells was discarded and washed with PBS once or twice. Then 10 mL of PBS containing 1% EDTAD was added for digestion for 1 min, the J774A.1 cells were blown and transferred to a 50 mL centrifuge tube at 1500 rpm for 10 min. The supernatant was discarded, and the retained substance was resuspended with 10 mL medium and then was counted to 5 × 10^5^/mL (divided into 12-well plates, 1 mL per well). The control, paeoniflorin, and paeoniflorin derivatives groups were set up with two multiple holes in each group. Opti-MEM (500 μL per well) was added, and J774A.1 cells were then pre-stimulated with 1% FBS and 50 ng/mL LPS for 3 h. Subsequently, five natural compounds and paeoniflorin derivatives were added for 30-minute intervention. J774A.1 cells were stimulated by MSU (150 µg/mL, 4 h), Nigericin (10 µg/mL, 30 min), ATP (2.5 mg/mL, 30 min), and poly-A: T (0.5 µg/mL, 4 h), respectively. After 30 min of J774A.1 cells were infected with Salmonella were then stimulated by gentamicin (50 µg/mL, 4 h). The supernatant was collected into a 1.5 mL EP tube, and 100 µL sample buffer was added to lyse the cells. The macrophage was bathed in a 101 °C metal for 10 min. Finally, the supernatant was centrifuged, and ELISA directly detected the cytokine.

### 3.4. Nitrite Assay

RAW264.7 cells were pretreated with the target compounds at the concentrations mentioned above for 2 h and treated with LPS (1 μg/mL) for 24 h. To measure the NO production, 50 μL of Griess reagent I (1% sulfanilamide containing 5% phosphoric acid) and Griess Reagent II (0.1% N-1-naphthyl ethylenediamine dihydrochloride) were successively added to the culture medium and incubated at room temperature for 10 min. The optical density was examined with the ELISA reader at 540 nm. Sodium nitrite at 0–100 μM was used as a standard to assess nitrite concentrations.

### 3.5. Statistical Analysis

For statistical analysis, data were expressed as mean ± SD from three independent experiments. Student’s paired *t*-test was used for statistical analyses, which were conducted using Prism 8 (GraphPad Software) and OriginPro 8 software package. Only *p* values < 0.05 were reported as significant.

### 3.6. Chemistry

All commercially sourced reagents were used as supplied unless otherwise stated. The ^1^H NMR and ^13^C NMR spectra were recorded on a Bruker Avance 400 spectrometer (400 MHz or 100 MHz, respectively). ^1^H NMR chemical shifts were reported in ppm (*δ*) relative to tetramethylsilane (TMS) with the solvent resonance employed as the internal standard (CDCl_3_, *δ* 7.26 ppm). ^13^C NMR chemical shifts were determined relative to the solvent: CDCl_3_ at *δ* 77.16 ppm. High-resolution mass spectroscopy data of the products were collected on a BioTOF Q instrument. Single-crystal (X-ray) diffraction measurements were conducted on Oxford Xcalibur E CCD X. Visualization on TLC was achieved by UV light (254 nm). Flash chromatography was performed on silica gel 200–300 mesh.

#### 3.6.1. Extraction of Natural Compounds

The dried roots (10 kg) of *P. lactiflora* were extracted with ethanol at room temperature. The concentrated syrup was suspended in H_2_O and then extracted successively with petroleum ether and ethyl acetate. The ethyl acetate extract (560 g) was chromatographed over a silica gel column and purified on an ODS silica gel column and preparative silica gel TLC to give natural compounds. Paeoniflorin, albiflorin, paeonin C, paeonivayin, and paeonin A were confirmed by comparing with standard samples and samples previously identified [33].

#### 3.6.2. General Procedure for the Synthesis of Paeoniflorin Derivatives

A solution of paeoniflorin (0.5 mmol) in alcohol (12 mL) was treated with Sc(CF_3_SO_3_)_3_ (0.5 mmol) and refluxed for 45 min. After completely consuming the substrates (monitored by TLC), the mixture was diluted with ethyl acetate and washed with saturated NaCl solution (×3). The organic layer was dried over Na_2_SO_4_ and concentrated under a vacuum. Then, the obtained mixture was acetylated for 1 h using an Ac_2_O-Py combination (1:1, 6 mL) at 0 °C. The reaction solution was diluted with ethyl acetate and washed with 5% H_2_SO_4_ solution, followed by water and saturated NaHCO_3_. The organic layer was dried over Na_2_SO_4_ and concentrated under a vacuum. The residue was purified by column chromatography to obtain Group I and Group II. Group I and Group II (0.09 mmol) were dissolved in CH_3_OH (3 mL), and Et_3_N (0.30 mL, 2.3 mmol) was added. After 24 h of reaction at room temperature and total consumption of substrates (monitored by TLC), the resulting mixture was diluted with CH_2_Cl_2_ and washed with saturated NaCl solution (×3). The organic layer was dried over Na_2_SO_4_ and concentrated under a vacuum. The residue was purified by column chromatography to obtain Group III, Group IV, Group V, and Group VI. Several compounds (compounds **1**, **2**, **3**, **4**, **6**, **7**, **8**, and **10**), which had been previously reported [29,33,34], were confirmed using only ^1^H NMR spectra and compared with previous data. Detailed physicochemical properties of paeoniflorin derivatives (see the Supplementary Materials for details):

**2,3,4,6-Tetra-*O*-acetyl-4-*O*-methylpaeoniflorin (1)**: white solid, 127.2 mg (42.4%, petroleum: ethyl acetate, 4:1), *R_f_* 0.38 (petroleum: ethyl acetate, 1:1), mp 119.0 °C, ^1^H NMR (400 MHz, Chloroform-*d*) δ 8.03 (d, *J* = 6.9 Hz 2H, H-2″, H-6″), 7.60 (t, *J* = 7.5 Hz, 1H, H-4″), 7.47 (t, *J* = 7.7 Hz, 2H, H-3″, H-5″), 5.46 (s, 1H, H-9), 5.13–4.96 (m, 3H, H-2′, H-3′, H-4′), 4.75 (d, *J* = 7.8 Hz, 1H, H-1′), 4.59 (d, *J* = 12.0 Hz, 1H, H-8), 4.47 (d, *J* = 12.0 Hz, 1H, H-8), 4.18 (dd, *J* = 12.2, 2.5 Hz, 1H, H-6′), 4.11 (dd, *J* = 12.2, 5.4 Hz, 1H, H-6′), 3.64–3.58 (m, 1H, H-5′), 3.41 (s, 3H, H-11), 2.77 (d, *J* = 6.9 Hz, 1H, H-5), 2.32 (dd, *J* = 10.7, 6.9 Hz, 1H, H-6), 2.10–1.93 (m, 14H, 4COCH_3_, H-3), 1.79 (d, *J* = 10.6 Hz, 1H, H-6), 1.36 (s, 3H, H-10). ESI-HRMS: *m/z* calcd for C_32_H_38_O_15_Na [M + Na]^+^ 685.2103, found 685.2111. 

**2,3,4,6-Tetra-*O*-acetyl-4-oxo-9-*O*-methylpaeoniflorin (2)**: white solid, 86.4 mg (28.8%, petroleum: ethyl acetate, 3:1), *R_f_* 0.30 (petroleum: ethyl acetate, 1:1), mp 177.8 °C, ^1^H NMR (400 MHz, Chloroform-*d*) δ 8.01 (d, *J* = 7.0 Hz, 2H, H-2″, H-6″), 7.63–7.57 (m, 1H, H-4″), 7.46 (t, *J* = 7.7 Hz, 2H, H-3″, H-5″), 5.16 (t, *J* = 9.4 Hz, 1H, H-3′), 5.08–5.02 (m, 2H, H-2′, H-4′), 5.01 (s, 1H, H-9), 4.78 (d, *J* = 7.9 Hz, 1H, H-1′), 4.51 (d, *J* = 1.6 Hz, 2H, H-8), 4.19 (dd, *J* = 12.2, 2.6 Hz, 1H, H-6′), 4.14 (d, *J* = 5.5 Hz, 1H, H-6′), 3.68–3.62 (m, 1H, H-5′), 3.35 (s, 3H, H-11), 3.04 (d, *J* = 7.3 Hz, 1H, H-5), 2.71 (t, *J* = 9.3 Hz, 1H, H-6), 2.65 (d, *J* = 6.0 Hz, 2H, H-6, H-3), 2.09–1.96 (m, 13H, 4COCH_3_, H-3), 1.40 (s, 3H, H-10). ESI-HRMS: *m/z* calcd for C_32_H_38_O_15_Na [M + Na]^+^ 685.2103, found 685.2150.

**4-*O*-methylpaeoniflorin (3)**: colorless syrup, 22.3 mg (44.5%, CH_2_Cl_2_: CH_3_OH, 22:1), *R_f_* 0.36 (CH_2_Cl_2_: CH_3_OH, 7:1), ^1^H NMR (400 MHz, Chloroform-*d*) δ 7.93 (d, *J* = 7.2 Hz, 2H, H-2″, H-6″), 7.49 (t, *J* = 7.4 Hz, 1H, H-4″), 7.35 (t, *J* = 7.7 Hz, 2H, H-3″, H-5″), 5.49 (s, 1H, H-9), 4.70–4.61 (m, 2H, H-8, H-1′), 4.50 (d, *J* = 7.7 Hz, 1H, H-1′), 3.80 (s, 2H, H-6′), 3.59 (s, 2H, H-3′, H-4′), 3.42 (s, 1H, H-2′), 3.34 (s, 4H, H-5′, H-11), 2.68 (d, *J* = 6.6 Hz, 1H, H-6), 2.39 (d, *J* = 10.0 Hz, 1H, H-5), 1.95 (s, 2H, H-3), 1.78 (d, *J* = 10.7 Hz, 1H, H-6), 1.34 (s, 3H, H-10). ESI-HRMS: *m/z* calcd for C_24_H_30_O_11_Na [M + Na]^+^ 517.1681, found 517.1663.

**4-Oxo-9-*O*-methylpaeoniflorin (4):** colorless syrup, 29.7 mg (48%, CH_2_Cl_2_: CH_3_OH, 21:1), *R_f_* 0.35 (CH_2_Cl_2_: CH_3_OH, 7:1), ^1^H NMR (400 MHz, Chloroform-*d*) δ 7.90 (d, *J* = 7.7 Hz, 2H, H-2″, H-6″), 7.51 (t, *J* = 7.4 Hz, 1H, H-4″), 7.35 (t, *J* = 7.7 Hz, 2H, H-3″, H-5″), 5.02 (s, 1H, H-9), 4.73 (d, *J* = 11.7 Hz, 1H, H-8), 4.56 (d, *J* = 12.8 Hz, 2H, H-8, H-1′), 3.84 (s, 2H, H-6′), 3.64 (s, 2H, H-3′, H-4′), 3.48 (s, 1H, H-2′), 3.35 (s, 1H, H-5′), 3.27 (s, 3H, H-11), 2.96 (d, *J* = 7.0 Hz, 1H, H-6), 2.80 (d, *J* = 10.6 Hz, 1H, H-5), 2.60 (q, *J* = 18.3 Hz, 2H, H-3), 2.00 (d, *J* = 10.9 Hz, 1H, H-6), 1.40 (s, 3H, H-10). ESI-HRMS: *m/z* calcd for C_24_H_30_O_11_Na [M + Na]^+^ 517.1681, found 517.1700.

**4-Oxo-9-*O*-methyldebenzoylpaeoniflorin (5):** white solid, 15.4 mg (31.5%, CH_2_Cl_2_: CH_3_OH, 16:1), *R_f_* 0.08 (CH_2_Cl_2_:CH_3_OH, 7:1), mp 162.9 °C, ^1^H NMR (400 MHz, Acetone-*d*_6_) δ 5.10 (s, 1H, H-9), 4.76 (d, *J* = 7.7 Hz, 1H, H-8), 4.02 (d, *J* = 12.6 Hz, 1H, H-6′), 3.81 (dd, *J* = 11.7, 2.6 Hz, 1H, H-6′), 3.72 (d, *J* = 12.5 Hz, 1H, H-2′), 3.62 (dd, *J* = 10.2, 4.2 Hz, 2H, H-4′, H-5′), 3.48 (t, *J* = 8.6 Hz, 1H, H-3′), 3.39–3.31 (m, 2H, H-8, H-1′), 3.26 (s, 3H, H-11), 2.90 (dd, *J* = 11.1, 7.3 Hz, 1H, H-5), 2.75 (d, *J* = 17.9 Hz, 1H, H-6), 2.48 (d, *J* = 7.5 Hz, 1H, H-3), 2.36 (d, *J* = 17.3 Hz, 1H, H-3), 2.07 (d, *J* = 4.2 Hz, 1H, H-6), 1.35 (s, 3H, H-10). ^13^C NMR (101 MHz, Acetone-*d*_6_) δ 205.27 (C-4), 105.31 (C-9), 98.93 (C-1′), 88.26 (C-1), 87.13 (C-2), 78.22 (C-3′), 77.54 (C-5′), 74.59 (C-2′), 71.77 (C-11), 65.21 (C-7), 62.97 (C-6′), 60.19 (C-8), 55.48 (C-4′), 47.17 (C-3), 46.66 (C-5), 26.67 (C-6), 20.75 (C-10). ESI-HRMS: *m/z* calcd for C_17_H_26_O_10_Na [M + Na]^+^ 413.1419, found 413.1402.

**2,3,4,6-Tetra-*O*-acetyl-4-*O*-ethylpaeoniflorin (6):** white solid, 103.7 mg (35.5%, petroleum: ethyl acetate, 5:1), *R_f_* 0.40 (petroleum: ethyl acetate, 1:1), mp 145.1 °C, ^1^H NMR (400 MHz, Chloroform-*d*) δ 8.03 (d, *J* = 7.1 Hz, 2H, H-2″, H-6″), 7.60 (t, *J* = 7.4 Hz, 1H, H-4″), 7.48 (t, *J* = 7.7 Hz, 2H, H-3″, H-5″), 5.44 (s, 1H, H-9), 5.12–4.95 (m, 3H, H-2′, H-3′, H-4′), 4.75 (d, *J* = 7.8 Hz, 1H, H-1′), 4.59 (d, *J* = 12.0 Hz, 1H, H-8), 4.47 (d, *J* = 12.0 Hz, 1H, H-8), 4.18 (dd, *J* = 12.2, 2.6 Hz, 1H, H-6′), 4.13–4.08 (m, 1H, H-6′), 3.69 (qd, *J* = 7.0, 2.7 Hz, 2H, H-11), 3.60 (ddd, *J* = 9.6, 5.3, 2.6 Hz, 1H, H-5′), 2.76 (d, *J* = 6.0 Hz, 1H, H-5), 2.32 (dd, *J* = 10.7, 6.9 Hz, 1H, H-6), 2.02 (dd, *J* = 21.9, 16.2 Hz, 14H, 4COCH_3_, H-3), 1.80 (d, *J* = 10.6 Hz, 1H, H-6), 1.35 (s, 3H, H-10), 1.21 (t, *J* = 7.0 Hz, 3H, H-12). ESI-HRMS: *m/z* calcd for C_33_H_40_O_15_Na [M + Na]^+^ 699.2260, found 699.2263.

**2,3,4,6-Tetra-*O*-acetyl-4-oxo-9-*O*-ethylpaeoniflorin (7):** white solid, 97.3 mg (33.4%, petroleum: ethyl acetate, 4:1), *R_f_* 0.32 (petroleum: ethyl acetate, 1:1), mp 161.8 °C, ^1^H NMR (400 MHz, Chloroform-*d*) δ 8.01 (d, *J* = 6.9 Hz, 2H, H-2″, H-6″), 7.59 (t, *J* = 7.4 Hz, 1H, H-4″), 7.45 (t, *J* = 7.8 Hz, 2H, H-3″, H-5″), 5.17 (t, *J* = 9.4 Hz, 1H, H-3′), 5.11 (s, 1H, H-9), 5.08–5.00 (m, 2H, H-2′, H-4′), 4.78 (d, *J* = 7.8 Hz, 1H, H-1′), 4.57–4.47 (m, 2H, H-8), 4.19 (dd, *J* = 12.3, 2.6 Hz, 1H, H-6′), 4.12 (dd, *J* = 12.2, 5.5 Hz, 1H, H-6′), 3.73 (dd, *J* = 9.6, 7.1 Hz, 1H, H-11), 3.68–3.62 (m, 1H, H-5′), 3.51–3.41 (m, 1H, H-11), 3.04 (d, *J* = 7.3 Hz, 1H, H-5), 2.70 (dd, *J* = 11.1, 7.4 Hz, 1H, H-6), 2.65 (d, *J* = 4.1 Hz, 2H, H-3), 2.09–1.96 (m, 13H, 4COCH_3_, H-6), 1.39 (s, 3H, H-10), 1.10 (t, *J* = 7.0 Hz, 3H, H-12). ESI-HRMS: *m/z* calcd for C_33_H_40_O_15_Na [M + Na]^+^ 699.2260, found 699.2271.

**4-*O*-ethylpaeoniflorin (8):** white solid, 14.5 mg (30.6%, CH_2_Cl_2_: CH_3_OH, 23:1), *R_f_* 0.35 (CH_2_Cl_2_: CH_3_OH, 7:1), mp 123.6 °C, ^1^H NMR (400 MHz, Chloroform-*d*) δ 7.94 (d, *J* = 7.8 Hz, 2H, H-2″, H-6″), 7.50 (t, *J* = 7.4 Hz, 1H, H-4″), 7.36 (t, *J* = 7.6 Hz, 2H, H-3″, H-5″), 5.48 (s, 1H, H-9), 4.66 (q, *J* = 12.1 Hz, 2H, H-8, H-1′), 4.49 (d, *J* = 7.5 Hz, 1H, H-8), 3.81 (d, *J* = 15.0 Hz, 2H, H-6′), 3.60 (dt, *J* = 19.5, 9.5 Hz, 4H, H-3′, H-4′, H-11), 3.43–3.36 (m, 1H, H-2′), 3.28 (d, *J* = 8.6 Hz, 1H, H-5′), 2.67 (d, *J* = 6.5 Hz, 1H, H-6), 2.37 (dd, *J* = 11.0, 6.9 Hz, 1H, H-5), 1.97 (s, 2H, H-3), 1.79 (d, *J* = 10.7 Hz, 1H, H-6), 1.33 (s, 3H, H-10), 1.17 (t, *J* = 7.0 Hz, 3H, H-12). ESI-HRMS: *m/z* calcd for C_25_H_32_O_11_Na [M + Na]^+^ 531.1837, found 531.1871.

**4-*O*-ethyldebenzoylpaeoniflorin (9):** colorless syrup, 26 mg (61.5%, CH_2_Cl_2_: CH_3_OH, 15:1), *R_f_* 0.07 (CH_2_Cl_2_: CH_3_OH, 7:1), ^1^H NMR (400 MHz, Acetone-*d*_6_) δ 5.21 (s, 1H, H-9), 4.65 (d, *J* = 7.7 Hz, 1H, H-8), 4.00 (d, *J* = 12.4 Hz, 1H, H-8), 3.90 (d, *J* = 12.3 Hz, 1H, H-1′), 3.81 (d, *J* = 11.5 Hz, 1H, H-6′), 3.67–3.58 (m, 3H, H-6′, H-11), 3.46 (t, *J* = 8.7 Hz, 1H, H-5′), 3.35–3.30 (m, 2H, H-3′, H-4′), 3.27–3.21 (m, 1H, H-2′), 2.49 (d, *J* = 8.6 Hz, 1H, H-6), 2.41 (dd, *J* = 10.6, 6.9 Hz, 1H, H-5), 1.98 (d, *J* = 17.3 Hz, 1H, H-3), 1.88–1.79 (m, 2H, H-6, H-3), 1.29 (s, 3H, H-10), 1.12 (t, *J* = 7.1 Hz, 3H, H-12). ^13^C NMR (101 MHz, Acetone-*d*_6_) δ 108.41 (C-4), 101.98 (C-9), 99.48 (C-1′), 88.96 (C-1), 86.10 (C-2), 78.14 (C-3′), 77.43 (C-5′), 74.77 (C-2′), 72.68 (C-11), 71.77 (C-4′), 62.99 (C-7), 59.51 (C-6′), 59.09 (C-8), 42.57 (C-3), 41.16 (C-5), 23.20 (C-12), 19.66 (C-6), 15.92 (C-10). ESI-HRMS: *m/z* calcd for C_18_H_28_O_10_Na [M + Na]^+^ 427.1575, found 427.1602.

**4-Oxo-9-*O*-ethylpaeoniflorin (10):** colorless syrup, 30 mg (26.8%, CH_2_Cl_2_: CH_3_OH, 22:1), *R_f_* 0.34 (CH_2_Cl_2_: CH_3_OH, 7:1), ^1^H NMR (400 MHz, Chloroform-*d*) δ 7.90 (d, *J* = 7.4 Hz, 2H, H-2″, H-6″), 7.50 (t, *J* = 7.5 Hz, 1H, H-4″), 7.34 (t, *J* = 7.7 Hz, 2H, H-3″, H-5″), 5.12 (s, 1H, H-9), 4.74 (d, *J* = 11.3 Hz, 1H, H-8), 4.55 (d, *J* = 7.2 Hz, 2H, H-8, H-1′), 3.83 (s, 2H, H-6′), 3.64 (p, *J* = 9.7, 8.4 Hz, 3H, H-2′, H-3′, H-4′), 3.48 (s, 1H, H-5′), 3.39–3.31 (m, 2H, H-11), 2.96 (d, *J* = 7.1 Hz, 1H, H-6), 2.80 (d, *J* = 10.2 Hz, 1H, H-5), 2.60 (q, *J* = 18.2 Hz, 2H, H-3), 2.00 (d, *J* = 10.8 Hz, 1H, H-6), 1.39 (s, 3H, H-10), 1.02 (t, *J* = 7.0 Hz, 3H, H-12). ESI-HRMS: *m/z* calcd for C_18_H_28_O_10_Na [M + Na]^+^ 531.1837, found 531.1852.

**4-Oxo-9-*O*-ethyldebenzoylpaeoniflorin (11):** white solid, 60 mg (67.5%, CH_2_Cl_2_: CH_3_OH, 16:1), mp 105.6 °C, *R_f_* 0.06 (CH_2_Cl_2_: CH_3_OH, 7:1), ^1^H NMR (400 MHz, Acetone-*d*_6_) δ 5.20 (s, 1H, H-9), 4.76 (d, *J* = 7.7 Hz, 1H, H-8), 4.02 (dd, *J* = 12.5, 3.6 Hz, 1H, H-6′), 3.85–3.78 (m, 1H, H-11), 3.76–3.58 (m, 3H, H-6′, H-11, H-5′), 3.52–3.41 (m, 2H, H-3′, H-4′), 3.37–3.25 (m, 3H, H-2′, H-8, H-1′), 2.88 (d, *J* = 6.5 Hz, 1H, H-5), 2.74 (d, *J* = 17.9 Hz, 1H, H-6), 2.49 (d, *J* = 7.5 Hz, 1H, H-3), 2.38 (d, *J* = 17.9 Hz, 1H, H-3), 2.06 (d, *J* = 3.1 Hz, 1H, H-6), 1.34 (s, 3H, H-10), 1.06 (t, *J* = 7.1 Hz, 3H, H-12). ^13^C NMR (101 MHz, Acetone-*d*_6_) δ 205.35 (C-4), 103.66 (C-9), 98.89 (C-1′), 88.18 (C-1), 86.98 (C-2), 78.25 (C-3′), 77.50 (C-5′), 74.58 (C-2′), 71.79 (C-11), 65.24 (C-4′), 63.91 (C-7), 63.00 (C-8), 60.23 (C-6′), 49.48 (C-3), 47.23 (C-5), 26.63 (C-6), 20.77 (C-10), 15.09 (C-12). ESI-HRMS: *m/z* calcd for C_18_H_28_O_10_Na [M + Na]^+^ 427.1575, found 427.1612.

**2,3,4,6-Tetra-*O*-acetyl-4-*O*-propylpaeoniflorin (12):** white solid, 57.2 mg (30.7%, petroleum: ethyl acetate, 4:1), mp 143.1 °C, *R_f_* 0.42 (petroleum: ethyl acetate, 1:1), ^1^H NMR (400 MHz, Chloroform-*d*) δ 8.03 (d, *J* = 7.0 Hz, 2H, H-2″, H-6″), 7.60 (t, *J* = 7.4 Hz, 1H, H-4″), 7.48 (t, *J* = 7.7 Hz, 2H, H-3″, H-5″), 5.44 (s, 1H, H-9), 5.11–4.95 (m, 3H, H-2′, H-3′, H-4′), 4.75 (d, *J* = 7.8 Hz, 1H, H-1′), 4.59 (d, *J* = 12.0 Hz, 1H, H-8), 4.48 (d, *J* = 12.0 Hz, 1H, H-8), 4.18 (dd, *J* = 12.2, 2.6 Hz, 1H, H-6′), 4.11 (dd, *J* = 12.2, 5.3 Hz, 1H, H-6′), 3.63–3.53 (m, 3H, H-11, H-5′), 2.77 (d, *J* = 6.8 Hz, 1H, H-5), 2.32 (dd, *J* = 10.7, 7.0 Hz, 1H, H-6), 2.02 (dd, *J* = 21.4, 16.8 Hz, 14H, 4COCH_3_, H-3), 1.80 (d, *J* = 10.7 Hz, 1H, H-6), 1.59 (q, *J* = 7.1 Hz, 2H, H-12), 1.35 (s, 3H, H-10), 0.90 (t, *J* = 7.4 Hz, 3H, H-13).^13^C NMR (101 MHz, Chloroform-*d*) δ 170.53, 170.31, 169.49, 169.40 (4CH_3_CO), 166.48 (C-7″), 133.54 (C-4″), 129.72 (C-2″, C-6″), 129.65 (C-1″), 128.73 (C-3″, C-5″), 107.62 (C-4), 101.16 (C-9), 96.41 (C-1′), 88.43 (C-1), 85.64 (C-2), 73.04 (C-3′), 71.81 (C-5′), 71.36 (C-2′), 69.83 (C-11), 68.47 (C-4′), 65.80 (C-7), 62.06 (C-6′), 60.25 (C-8), 41.60 (C-3), 40.90 (C-5), 23.21 (C-12), 22.31 (C-6), 20.82, 20.67, 20.65×2 (4CH_3_CO), 19.22 (C-10), 10.47 (C-13). ESI-HRMS: *m/z* calcd for C_34_H_42_O_15_Na [M + Na]^+^ 713.2416, found 713.2493.

**2,3,4,6-Tetra-*O*-acetyl-4-oxo-9-*O*-propylpaeoniflorin (13):** colorless syrup, 73.4 mg (39.4%, petroleum: ethyl acetate, 2:1), *R_f_* 0.34 (petroleum: ethyl acetate, 1:1), ^1^H NMR (400 MHz, Chloroform-*d*) δ 8.01 (d, *J* = 7.0 Hz, 2H, H-2″, H-6″), 7.59 (t, *J* = 7.5 Hz, 1H, H-4″), 7.45 (t, *J* = 7.8 Hz, 2H, H-3″, H-5″), 5.17 (t, *J* = 9.4 Hz, 1H, H-3′), 5.11 (s, 1H, H-9), 5.04 (dd, *J* = 16.9, 9.2 Hz, 2H, H-2′′, H-4′′), 4.78 (d, *J* = 7.9 Hz, 1H, H-1′), 4.56–4.47 (m, 2H, H-8), 4.19 (dd, *J* = 12.2, 2.5 Hz, 1H, H-6′), 4.13 (dd, *J* = 12.2, 5.5 Hz, 1H, H-6′), 3.64 (dt, *J* = 9.6, 6.7 Hz, 2H, H-11, H-5′), 3.35 (dt, *J* = 9.3, 6.4 Hz, 1H, H-11), 3.05 (d, *J* = 7.4 Hz, 1H, H-5), 2.73–2.67 (m, 1H, H-6), 2.65 (d, *J* = 4.4 Hz, 2H, H-3), 2.10–1.97 (m, 13H, 4COCH_3_, H-6), 1.49 (q, *J* = 6.9 Hz, 2H, H-12), 1.39 (s, 3H, H-10), 0.83 (t, *J* = 7.4 Hz, 3H, H-13).^13^C NMR (101 MHz, Chloroform-*d*) δ 204.89 (C-4), 170.48, 170.32, 169.51, 169.42 (4CH_3_CO), 166.46 (C-7″), 133.58 (C-4″), 129.78 (C-2″, C-6″), 129.51 (C-1″), 128.66 (C-3″, C-5″), 104.88 (C-9), 96.29 (C-1′), 87.93 (C-1), 85.78 (C-2), 72.98 (C-3′), 72.04 (C-5′), 71.51 (C-2′), 70.53 (C-11), 68.40 (C-4′), 63.16 (C-7), 62.41 (C-8), 62.08 (C-6′), 48.91 (C-3), 46.91 (C-5), 26.28 (C-6), 22.69 (C-12), 20.81, 20.73, 20.68×2 (4CH_3_CO), 20.47 (C-10), 10.72 (C-13). ESI-HRMS: *m/z* calcd for C_34_H_42_O_15_Na [M + Na]^+^ 713.2416, found 713.2420.

**4-*O*-propylpaeoniflorin (14):** colorless syrup, 25.3 mg (44.1%, CH_2_Cl_2_: CH_3_OH, 22:1), *R_f_* 0.32 (CH_2_Cl_2_: CH_3_OH, 8:1), ^1^H NMR (400 MHz, Chloroform-*d*) δ 7.93 (d, *J* = 7.4 Hz, 2H, H-2″, H-6″), 7.49 (t, *J* = 7.4 Hz, 1H, H-4″), 7.34 (t, *J* = 7.7 Hz, 2H, H-3″, H-5″), 5.45 (s, 1H, H-9), 4.65 (q, *J* = 12.2 Hz, 2H, H-8, H-1′), 4.49 (d, *J* = 7.6 Hz, 1H, H-8), 3.78 (q, *J* = 11.8 Hz, 2H, H-6′), 3.57 (s, 2H, H-3′, H-4′), 3.50 (t, *J* = 5.5 Hz, 2H, H-11), 3.39 (s, 1H, H-2′), 3.27 (s, 1H, H-5′), 2.66 (d, *J* = 6.4 Hz, 1H, H-6), 2.36 (s, 1H, H-5), 1.95 (s, 2H, H-3), 1.78 (d, *J* = 10.6 Hz, 1H, H-6), 1.54 (q, *J* = 7.2 Hz, 2H, H-12), 1.32 (s, 3H, H-10), 0.86 (t, *J* = 7.4 Hz, 3H, H-13). ^13^C NMR (101 MHz, Chloroform-*d*) δ 167.13 (C-7″), 133.47 (C-4″), 129.85 (C-2″, C-6″), 129.61 (C-1″), 128.64 (C-3″, C-5″), 107.70 (C-4), 101.25 (C-9), 98.82 (C-1′), 88.41 (C-1), 85.72 (C-2), 76.22 (C-3′), 75.72 (C-5′), 73.61 (C-2′), 70.12 (C-11), 69.70 (C-4′), 65.55 (C-7), 61.61 (C-6′), 60.93 (C-8), 41.78 (C-3), 40.33 (C-5), 23.23 (C-12), 19.53 (C-6), 19.53 (C-10), 10.49 (C-13). ESI-HRMS: *m/z* calcd for C_26_H_34_O_11_Na [M + Na]^+^ 545.1994, found 545.2017.

**4-Oxo-9-*O*-propylpaeoniflorin (15):** white solid, 35.3 mg (30.7%, CH_2_Cl_2_: CH_3_OH = 22:1), mp 105.1 °C, *R_f_* 0.36 (CH_2_Cl_2_: CH_3_OH, 7:1), ^1^H NMR (400 MHz, Chloroform-*d*) δ 7.90 (d, *J* = 7.1 Hz, 2H, H-2″, H-6″), 7.50 (t, *J* = 7.4 Hz, 1H, H-4″), 7.33 (t, *J* = 7.7 Hz, 2H, H-3″, H-5″), 5.10 (s, 1H, H-9), 4.75 (d, *J* = 11.7 Hz, 1H, H-8), 4.60–4.52 (m, 2H, H-8, H-1′), 3.84 (s, 2H, H-6′), 3.66 (s, 2H, H-3′, H-4′), 3.53 (m, 2H, H-11), 3.36 (d, *J* = 8.1 Hz, 1H, H-2′), 3.27–3.20 (m, 1H, H-5′), 2.97 (d, *J* = 7.1 Hz, 1H, H-6), 2.83–2.75 (m, 1H, H-5), 2.59 (q, *J* = 18.2 Hz, 2H, H-3), 1.99 (d, *J* = 10.9 Hz, 1H, H-6), 1.41 (d, *J* = 10.2 Hz, 5H, H-10, H-12), 0.77 (t, *J* = 7.4 Hz, 3H, H-13). ^13^C NMR (101 MHz, Chloroform-*d*) δ 205.24 (C-4), 167.28 (C-7″), 133.70 (C-4″), 129.81 (C-2″, C-6″), 129.33 (C-1″), 128.62 (C-3″, C-5″), 105.30 (C-9), 98.73 (C-1′), 87.97 (C-1), 85.82 (C-2),76.53 (C-3′), 75.89 (C-5′), 73.63 (C-2′), 70.42 (C-11), 69.52 (C-4′), 63.48 (C-7), 63.30 (C-8), 61.61 (C-6′), 48.85 (C-3), 46.98 (C-5), 26.59 (C-6), 22.69 (C-12), 20.72 (C-10), 10.73 (C-13). ESI-HRMS: *m/z* calcd for C_26_H_34_O_11_Na [M + Na]^+^ 545.1994, found 545.2075.

**4-Oxo-9-*O*-propyldebenzoylpaeoniflorin (16):** white solid, 46.7 mg (40.6%, CH_2_Cl_2_: CH_3_OH = 15:1), mp 133.3 °C, *R_f_* 0.05 (CH_2_Cl_2_: CH_3_OH, 7:1), ^1^H NMR (400 MHz, Acetone-*d*_6_) δ 5.19 (s, 1H, H-9), 4.76 (d, *J* = 7.7 Hz, 1H, H-8), 4.02 (d, *J* = 12.6 Hz, 1H, H-6′), 3.81 (dd, *J* = 11.8, 2.6 Hz, 1H, H-11), 3.71 (d, *J* = 12.6 Hz, 1H, H-6′), 3.64–3.55 (m, 2H, H-5′, H-11), 3.48 (t, *J* = 8.7 Hz, 1H, H-3′), 3.40–3.32 (m, 3H, H-2′, H-4′, H-8), 3.27 (d, *J* = 9.0 Hz, 1H, H-1′), 2.90 (dd, *J* = 12.3, 7.6 Hz, 1H, H-5), 2.75 (d, *J* = 17.9 Hz, 1H, H-6), 2.49 (d, *J* = 7.4 Hz, 1H, H-3), 2.38 (d, *J* = 16.9 Hz, 1H, H-3), 2.09 (s, 1H, H-6), 1.47 (q, *J* = 6.9 Hz, 2H, H-12), 1.35 (s, 3H, H-10), 0.84 (t, *J* = 7.4 Hz, 3H, H-13). ^13^C NMR (101 MHz, Acetone-*d*_6_) δ 205.32 (C-4), 104.10 (C-9), 98.89 (C-1′), 88.21 (C-1), 86.96 (C-2), 78.17 (C-3′), 77.50 (C-5′), 74.53 (C-2′), 71.71 (C-11), 70.62 (C-4′), 65.34 (C-7), 62.92 (C-6′), 60.17 (C-8), 49.52 (C-3), 47.20 (C-5), 26.63 (C-6), 23.46 (C-12), 20.78 (C-10), 11.01 (C-13). ESI-HRMS: *m/z* calcd for C_19_H_30_O_10_Na [M + Na]^+^ 441,1732, found 441.1733.

**2,3,4,6-Tetra-*O*-acetyl-4-*O*-iso-propylpaeoniflorin (17):** white solid, 61mg (37.5%, petroleum: ethyl acetate = 4:1), mp 117.8 °C, *R_f_* 0.40 (petroleum: ethyl acetate, 1:1), mp 113–115 °C, ^1^H NMR (400 MHz, Chloroform-*d*) δ 8.03 (d, *J* = 7.0 Hz, 2H, H-2″, H-6″), 7.60 (t, *J* = 7.4 Hz, 1H, H-4″), 7.48 (t, *J* = 7.7 Hz, 2H, H-3″, H-5″), 5.42 (s, 1H, H-9), 5.08–4.96 (m, 3H, H-2′, H-3′, H-4′), 4.74 (d, *J* = 7.7 Hz, 1H, H-1′), 4.59 (d, *J* = 11.9 Hz, 1H, H-8), 4.49 (d, *J* = 12.0 Hz, 1H, H-8), 4.17 (dd, *J* = 12.3, 2.7 Hz, 1H, H-6′), 4.11 (dd, *J* = 12.2, 5.6 Hz, 2H, H-6′, H-11), 3.62–3.56 (m, 1H, H-5′), 2.73 (d, *J* = 6.7 Hz, 1H, H-5), 2.32 (dd, *J* = 10.7, 7.0 Hz, 1H, H-6), 2.17 (d, *J* = 3.3 Hz, 1H, H-3), 2.07, 2.01, 1.97 (13H, 4COCH_3_, H-3), 1.80 (d, *J* = 10.6 Hz, 1H, H-6), 1.34 (s, 3H, H-10), 1.18 (dd, *J* = 6.2, 2.7 Hz, 6H, H-12, H-13). ^13^C NMR (101 MHz, Chloroform-*d*) δ 170.54, 170.32, 169.50, 169.40 (4CH_3_CO), 166.50 (C-7″), 133.55 (C-4″), 129.73 (C-2″, C-6″, C-1″), 128.76 (C-3″, C-5″), 107.81 (C-4), 101.15 (C-9), 96.44 (C-1′), 88.39 (C-1), 85.70 (C-2), 73.09 (C-3′), 71.83 (C-5′), 71.39 (C-2′), 69.57 (C-11), 68.51 (C-4′), 67.44 (C-7), 62.08 (C-6′), 60.34 (C-8), 42.14 (C-3), 41.88 (C-5), 24.26 (C-12), 24.13 (C-13), 22.40 (C-6), 20.83, 20.70, 20.68, 20.66 (4CH_3_CO), 19.26 (C-10). ESI-HRMS: *m/z* calcd for C_34_H_42_O_15_Na [M + Na]^+^ 713.2416, found 713.2490.

**2,3,4,6-Tetra-*O*-acetyl-4-oxo-9-*O*-iso-propylpaeoniflorin (18):** colorless syrup, 62 mg (38.1%, petroleum: ethyl acetate = 3:1), *R_f_* 0.36 (petroleum: ethyl acetate, 1:1), mp 147–149 °C, ^1^H NMR (400 MHz, Chloroform-*d*) δ 8.01 (d, *J* = 7.0 Hz, 2H, H-2″, H-6″), 7.58 (t, *J* = 7.5 Hz, 1H, H-4″), 7.44 (t, *J* = 7.6 Hz, 2H, H-3″, H-5″), 5.20 (s, 1H, H-9), 5.15 (d, *J* = 9.4 Hz, 1H, H-2′), 5.07–4.99 (m, 2H, H-3′, H-4′), 4.77 (d, *J* = 7.9 Hz, 1H, H-1′), 4.51 (q, *J* = 11.8 Hz, 2H, H-8), 4.18 (dd, *J* = 12.2, 2.4 Hz, 1H, H-6′), 4.12 (dd, *J* = 12.2, 5.3 Hz, 1H, H-6′), 3.91 (p, *J* = 6.2 Hz, 1H, H-11), 3.68–3.62 (m, 1H, H-5′), 3.03 (d, *J* = 7.3 Hz, 1H, H-5), 2.67 (m, 3H, H-6, H-3), 2.08, 2.05, 2.02, 1.99 (13H, 4COCH_3_, H-3), 1.37 (s, 3H, H-10), 1.11 (d, *J* = 6.2 Hz, 3H, H-12), 1.00 (d, *J* = 6.1 Hz, 3H, H-13). ^13^C NMR (101 MHz, Chloroform-*d*) δ ^13^C NMR (101 MHz, Chloroform-*d*) δ 205.05 (C-4), 170.48, 170.33, 169.51, 169.43 (4CH_3_CO), 166.48 (C-7″), 133.58 (C-4″), 129.82 (C-2″, C-6″), 129.50 (C-1″), 128.65 (C-3″, C-5″), 102.42 (C-9), 96.29 (C-1′), 87.91 (C-1), 85.61 (C-2), 73.00 (C-3′), 72.04 (C-5′), 71.53 (C-2′), 70.13 (C-11), 68.42 (C-4′), 62.90 (C-7), 62.40 (C-8), 62.10 (C-6′), 49.01 (C-3), 46.97 (C-5), 26.26 (C-6), 22.54 (C-12), 21.32 (C-13), 20.82, 20.75, 20.69×2 (4CH_3_CO), 20.54 (C-10). ESI-HRMS: *m/z* calcd for C_34_H_42_O_15_Na [M + Na]^+^ 713.2416, found 713.2419.

**4-*O*-iso-propylpaeoniflorin (19):** colorless syrup, 24.7 mg (52.5%, CH_2_Cl_2_: CH_3_OH, 23:1), *R_f_* 0.39 (CH_2_Cl_2_: CH_3_OH, 7:1), ^1^H NMR (400 MHz, Chloroform-*d*) δ 7.94 (d, *J* = 6.9 Hz, 2H, H-2″, H-6″), 7.50 (t, *J* = 7.4 Hz, 1H, H-4″), 7.36 (t, *J* = 7.6 Hz, 2H, H-3″, H-5″), 5.46 (s, 1H, H-9), 4.70–4.60 (m, 2H, H-8, H-1′), 4.49 (d, *J* = 7.4 Hz, 1H, H-8), 4.05 (p, *J* = 6.1, 5.6 Hz, 1H, H-11), 3.79 (d, *J* = 9.3 Hz, 2H, H-6′), 3.54 (d, *J* = 10.6 Hz, 2H, H-3′, H-4′), 3.39 (s, 1H, H-2′), 3.26 (d, *J* = 8.6 Hz, 1H, H-5′), 2.64 (d, *J* = 6.6 Hz, 1H, H-6), 2.37 (dd, *J* = 10.9, 6.9 Hz, 1H, H-5), 1.96 (s, 2H, H-3), 1.79 (d, *J* = 10.7 Hz, 1H, H-6), 1.32 (s, 3H, H-10), 1.14 (d, *J* = 6.1 Hz, 6H, H-12, H-13). ^13^C NMR (101 MHz, Chloroform-*d*) δ 167.11 (C-7″), 133.45 (C-4″), 129.84 (C-2″, C-6″), 129.68 (C-1″), 128.63 (C-3″, C-5″), 107.87 (C-4), 101.19 (C-9), 98.91 (C-1′), 88.41 (C-1), 85.76 (C-2), 76.39 (C-3′), 75.73 (C-5′), 73.51 (C-2′), 69.84 (C-11), 69.53 (C-4′), 67.19 (C-7), 61.69 (C-6′), 60.92 (C-8), 42.34 (C-3), 41.21 (C-5), 24.25 (C-12), 24.13 (C-13), 22.80 (C-6), 19.55 (C-10). ESI-HRMS: *m/z* calcd for C_26_H_34_O_11_Na [M + Na]^+^ 545.1994, found 545.2083.

**4-*O*-iso-propyldebenzoylpaeoniflorin (20):** colorless syrup, 12.2 mg (32.4%, CH_2_Cl_2_: CH_3_OH, 16:1), *R_f_* 0.08 (CH_2_Cl_2_: CH_3_OH, 7:1), ^1^H NMR (400 MHz, Acetone-*d*_6_) δ 5.20 (s, 1H, H-9), 4.65 (d, *J* = 7.7 Hz, 1H, H-8), 3.99 (d, *J* = 12.3 Hz, 1H, H-8), 3.88 (d, *J* = 12.4 Hz, 1H, H-1′), 3.81 (dd, *J* = 11.6, 2.1 Hz, 1H, H-6′), 3.61 (dd, *J* = 11.6, 5.3 Hz, 1H, H-11), 3.43 (t, *J* = 8.3 Hz, 1H, H-5′), 3.37–3.30 (m, 3H, H-3′, H-4′, H-6′), 3.25–3.20 (m, 1H, H-2′), 2.46–2.37 (m, 2H, H-5, H-6), 2.02 (d, *J* = 12.3 Hz, 1H, H-3), 1.87–1.79 (m, 2H, H-3, H-6), 1.28 (s, 3H, H-10), 1.11 (dd, *J* = 6.1, 1.6 Hz, 6H, H-12, H-13). ^13^C NMR (101 MHz, Acetone-*d*_6_) δ 108.58 (C-4), 101.96 (C-9), 99.47 (C-1′), 88.88 (C-1), 86.07 (C-2), 78.27 (C-3′), 77.46 (C-5′), 74.81 (C-2′), 72.43 (C-11), 71.82 (C-4′), 66.91 (C-7), 63.04 (C-6′), 59.12 (C-8), 43.07 (C-3), 41.91 (C-5), 24.55 (C-12), 24.39 (C-13), 23.16 (C-6), 19.65 (C-10). ESI-HRMS: *m/z* calcd for C_19_H_30_O_10_Na [M + Na]^+^ 441.1732, found 441.1787.

**4-Oxo-9-*O*-iso-propylpaeoniflorin (21):** colorless syrup, 18.3 mg (38.9%, CH_2_Cl_2_: CH_3_OH, 22:1), *R_f_* 0.34 (CH_2_Cl_2_: CH_3_OH, 7:1), ^1^H NMR (400 MHz, Chloroform-*d*) δ 7.92 (d, *J* = 7.3 Hz, 2H, H-2″, H-6″), 7.50 (t, *J* = 7.5 Hz, 1H, H-4″), 7.34 (t, *J* = 7.7 Hz, 2H, H-3″, H-5″), 5.21 (s, 1H, H-9), 4.78 (d, *J* = 11.7 Hz, 1H, H-8), 4.58–4.51 (m, 2H, H-8, H-1′), 3.81 (dd, *J* = 11.2, 5.1 Hz, 3H, H-6′, H-11), 3.68–3.61 (m, 2H, H-3′, H-4′), 3.49 (s, 1H, H-2′), 3.35 (s, 1H, H-5′), 2.95 (d, *J* = 7.0 Hz, 1H, H-6), 2.78 (t, *J* = 9.3 Hz, 1H, H-5), 2.67–2.53 (m, 2H, H-3), 1.98 (d, *J* = 10.8 Hz, 1H, H-6), 1.38 (s, 3H, H-10), 1.05 (d, *J* = 6.2 Hz, 3H, H-12), 0.90 (d, *J* = 6.1 Hz, 3H, H-13).^13^C NMR (101 MHz, Chloroform-*d*) δ 205.42 (C-4), 167.31 (C-7″), 133.85 (C-4″), 129.89 (C-2″,C-6″), 129.33 (C-1″), 128.64 (C-3″, C-5″), 102.97 (C-9), 98.73 (C-1′), 87.86 (C-1), 85.67 (C-2),76.47 (C-3′), 75.93 (C-5′), 73.69 (C-2′), 70.22 (C-11), 69.72 (C-4′), 63.45 (C-7), 63.12(C-8), 61.67 (C-6′), 49.01 (C-3), 45.80 (C-5), 26.76 (C-6), 22.58 (C-12), 21.39 (C-13), 20.78 (C-10). ESI-HRMS: *m/z* calcd for C_26_H_34_O_11_Na [M + Na]^+^ 545.1994, found 545.2084.

**4-Oxo-9-*O*-iso-propyldebenzoylpaeoniflorin (22):** colorless syrup, 14.5 mg (38.5%, CH_2_Cl_2_: CH_3_OH, 15:1), *R_f_* 0.04 (CH_2_Cl_2_: CH_3_OH, 7:1), ^1^H NMR (400 MHz, Acetone-*d*_6_) δ 5.32 (s, 1H, H-9), 4.76 (d, *J* = 7.7 Hz, 1H, H-8), 4.01 (d, *J* = 12.5 Hz, 1H, H-6′), 3.81 (dd, *J* = 11.7, 2.6 Hz, 1H, H-11), 3.70 (d, *J* = 12.5 Hz, 1H, H-6′), 3.62 (dd, *J* = 11.7, 5.6 Hz, 1H, H-5′), 3.48 (t, *J* = 8.6 Hz, 1H, H-3), 3.40–3.34 (m, 2H, H-2′, H-4′), 3.33–3.25 (m, 2H, H-8, H-1′), 2.89 (dd, *J* = 11.2, 7.4 Hz, 1H, H-5), 2.74 (d, *J* = 18.2 Hz, 1H, H-6), 2.47–2.38 (m, 2H, H-3), 2.06 (s, 1H, H-6), 1.34 (s, 3H, H-10), 1.05 (dd, *J* = 7.8, 6.2 Hz, 6H, H-12, H-13). ^13^C NMR (101 MHz, Acetone-*d*_6_) δ 205.48 (C-4), 101.61 (C-9), 98.87 (C-1′), 87.72 (C-1), 86.89 (C-2), 78.16 (C-3′), 77.48 (C-5′), 74.52 (C-2′), 71.70 (C-11), 69.93 (C-4′), 65.09 (C-7), 62.91 (C-6′), 60.07 (C-8), 49.59 (C-3), 47.24 (C-5), 26.69 (C-6), 22.91 (C-12), 21.64 (C-13), 20.83 (C-10). ESI-HRMS: *m/z* calcd for C_19_H_30_O_10_Na [M + Na]^+^ 441,1732, found 441.1783.

**2,3,4,6-Tetra-*O*-acetyl-4-*O*-butylpaeoniflorin (23):** white solid, 31.4 mg (17.7%, petroleum: ethyl acetate, 4:1), mp 108.4 °C, *R_f_* 0.39 (petroleum: ethyl acetate, 1:1), ^1^H NMR (400 MHz, Chloroform-*d*) δ 8.03 (d, *J* = 7.1 Hz, 2H, H-2″, H-6″), 7.60 (d, *J* = 14.9 Hz, 1H, H-4″), 7.47 (t, *J* = 7.7 Hz, 2H, H-3″, H-5″), 5.44 (s, 1H, H-9), 5.12–4.96 (m, 3H, H-2′, H-3′, H-4′), 4.75 (d, *J* = 7.8 Hz, 1H, H-1′), 4.59 (d, *J* = 12.0 Hz, 1H, H-8), 4.48 (d, *J* = 12.0 Hz, 1H, H-8), 4.18 (dd, *J* = 12.2, 2.6 Hz, 1H, H-6′), 4.11 (dd, *J* = 12.2, 5.3 Hz, 1H, H-6′), 3.61 (tt, *J* = 5.3, 2.8 Hz, 3H, H-11, H-5′), 2.77 (d, *J* = 8.5 Hz, 1H, H-5), 2.32 (dd, *J* = 10.7, 6.9 Hz, 1H, H-6), 2.07, 2.03, 2.02, 1.98 (m, 14H, 4COCH_3_, H-3), 1.79 (d, *J* = 10.7 Hz, 1H, H-6), 1.58–1.50 (m, 2H, H-13), 1.35 (m, 3H, H-12, H-10), 0.90 (t, *J* = 7.4 Hz, 3H, H-14). ^13^C NMR (101 MHz, Chloroform-*d*) δ 170.58, 170.36, 169.53, 169.43 (4CH_3_CO), 166.52 (C-7″), 133.58 (C-4″), 129.75 (C-2″, C-6″), 129.67 (C-1″), 128.76 (C-3″, C-5″), 107.65 (C-4), 101.20 (C-9), 96.44 (C-1′), 88.45 (C-1), 85.67 (C-2), 73.08 (C-3′), 71.84 (C-5′), 71.39 (C-2′), 69.86 (C-11), 68.49 (C-4′), 64.03 (C-7), 62.09 (C-6′), 60.27 (C-8), 41.63 (C-3), 40.92 (C-5), 32.01 (C-12), 22.34 (C-6), 20.86×2, 20.72×2 (4CH_3_CO), 20.69 (C-10), 19.28 (C-13), 13.94 (C-14). ESI-HRMS: *m/z* calcd for C_35_H_44_O_15_Na [M + Na]^+^ 727.2573, found 727.2635.

**2,3,4,6-Tetra-*O*-acetyl-4-oxo-9-*O*-butylpaeoniflorin (24):** white solid, 72.6 mg (40.9%, petroleum: ethyl acetate, 3:1). *R_f_* 0.35 (petroleum: ethyl acetate, 1:1), mp 119.7 °C, ^1^H NMR (400 MHz, Chloroform-*d*) δ 8.01 (d, *J* = 8.5 Hz, 2H, H-2″, H-6″), 7.59 (t, *J* = 7.4 Hz, 1H, H-4″), 7.45 (t, *J* = 7.7 Hz, 2H, H-3″, H-5″), 5.17 (t, *J* = 9.4 Hz, 1H, H-2′), 5.10 (s, 1H, H-9), 5.08–5.00 (m, 2H, H-3′, H-4′), 4.78 (d, *J* = 7.9 Hz, 1H, H-1′), 4.56–4.47 (m, 2H, H-8), 4.19 (dd, *J* = 12.2, 2.5 Hz, 1H, H-6′), 4.13 (dd, *J* = 12.2, 5.4 Hz, 1H, H-6′), 3.72–3.62 (m, 2H, H-11, H-5′), 3.37 (dt, *J* = 9.5, 6.4 Hz, 1H, H-11), 3.04 (d, *J* = 7.3 Hz, 1H, H-5), 2.73–2.67 (m, 1H, H-6), 2.65 (d, *J* = 5.7 Hz, 2H, H-3,H-6), 2.08, 2.05, 2.03, 2.00 (m, 13H, 4COCH_3_, H-3), 1.44 (dt, *J* = 8.8, 6.2 Hz, 2H, H-13), 1.39 (s, 3H, H-10), 1.26 (d, *J* = 7.8 Hz, 2H, H-12), 0.83 (t, *J* = 7.3 Hz, 3H, H-14).^13^C NMR (101 MHz, Chloroform-*d*) δ 204.90 (C-4), 170.52, 170.37, 169.54, 169.46 (4CH_3_CO), 166.50 (C-7″), 133.61(C-4″), 129.83 (C-2″, C-6″), 129.53 (C-1″), 128.68 (C-3″, C-5″), 104.94 (C-9), 96.32 (C-1′), 87.96 (C-1), 85.77 (C-2), 73.01 (C-3′), 72.08 (C-5′), 71.54 (C-2′), 68.64 (C-11), 68.41 (C-4′), 63.15 (C-7), 62.43 (C-8), 62.11 (C-6′), 48.94 (C-3), 46.92 (C-5), 31.48 (C-12), 26.31 (C-6), 20.85, 20.77, 20.72×2 (4CH_3_CO), 20.51 (C-10), 19.32 (C-13), 13.92 (C-14). ESI-HRMS: *m/z* calcd for C_35_H_44_O_15_Na [M + Na]^+^ 727.2573, found 727.2633.

**4-*O*-butylpaeoniflorin (25):** colorless syrup, 21.7 mg (44.9%, CH_2_Cl_2_: CH_3_OH, 22:1), *R_f_* 0.34 (CH_2_Cl_2_: CH_3_OH, 7:1). ^1^H NMR (400 MHz, Chloroform-*d*) δ 7.92 (d, *J* = 7.2 Hz, 2H, H-2″, H-6″), 7.48 (t, *J* = 7.5 Hz, 1H, H-4″), 7.34 (t, *J* = 7.6 Hz, 2H, H-3″, H-5″), 5.46 (s, 1H, H-9), 4.66 (t, *J* = 8.5 Hz, 2H, H-8, H-1′), 4.50 (d, *J* = 7.2 Hz, 1H, H-8), 3.79 (d, *J* = 10.4 Hz, 2H, H-6′), 3.62–3.47 (m, 4H, H-3′, H-4′, H-11), 3.41 (s, 1H, H-2′), 3.29 (s, 1H, H-5′), 2.66 (d, *J* = 6.3 Hz, 1H, H-6), 2.38 (d, *J* = 10.0 Hz, 1H, H-5), 1.94 (s, 2H, H-3), 1.78 (d, *J* = 10.4 Hz, 1H, H-6), 1.50 (p, *J* = 6.9 Hz, 2H, H-12), 1.36–1.27 (m, 5H, H-10, H-13), 0.87 (t, *J* = 7.3 Hz, 3H, H-14). ^13^C NMR (101 MHz, Chloroform-*d*) δ 167.12 (C-7″), 133.45 (C-4″), 129.85 (C-2″, C-6″), 129.64 (C-1″), 128.62 (C-3″, C-5″), 107.72 (C-4), 101.25 (C-9), 98.90 (C-1′), 88.48 (C-1), 85.73 (C-2), 76.38 (C-3′), 75.75 (C-5′), 73.57 (C-2′), 70.10 (C-11), 69.53 (C-4′), 63.74 (C-7), 61.67 (C-6′), 60.94 (C-8), 41.81 (C-3), 40.27 (C-5), 32.01 (C-12), 29.84 (C-6), 19.55 (C-10), 19.26 (C-13), 13.94 (C-14). ESI-HRMS: *m/z* calcd for C_27_H_36_O_11_Na [M + Na]^+^ 559.2150, found 559.2115.

**4-Oxo-9-*O*-butylpaeoniflorin (26):** colorless syrup, 25.1 mg (42.5%, CH_2_Cl_2_: CH_3_OH, 23:1), *R_f_* 0.35 (CH_2_Cl_2_: CH_3_OH, 7:1), ^1^H NMR (400 MHz, Chloroform-*d*) δ 7.90 (d, *J* = 7.2 Hz, 2H, H-2″, H-6″), 7.50 (t, *J* = 7.5 Hz, 1H, H-4″), 7.33 (t, *J* = 7.7 Hz, 2H, H-3″, H-5″), 5.09 (s, 1H, H-9), 4.75 (d, *J* = 11.7 Hz, 1H, H-8), 4.59–4.51 (m, 2H, H-8, H-1′), 3.84 (s, 2H, H-6′), 3.66 (d, *J* = 6.9 Hz, 2H, H-11), 3.58 (t, *J* = 7.9 Hz, 1H, H-3′), 3.50 (s, 1H, H-4′), 3.38–3.34 (m, 1H, H-2′), 3.25 (q, *J* = 7.3, 6.5 Hz, 1H, H-5′), 2.97 (d, *J* = 7.1 Hz, 1H, H-6), 2.79 (d, *J* = 9.6 Hz, 1H, H-5), 2.67–2.49 (m, 2H, H-3), 1.99 (d, *J* = 10.8 Hz, 1H, H-6), 1.36 (d, *J* = 20.3 Hz, 5H, H-10, H-12), 1.18 (q, *J* = 7.5 Hz, 2H, H-13), 0.77 (t, *J* = 7.3 Hz, 3H, H-14). ^13^C NMR (101 MHz, Chloroform-*d*) δ 205.17 (C-4), 167.30 (C-7″), 133.71 (C-4″), 129.85 (C-2″, C-6″), 129.37 (C-1″), 128.63 (C-3″, C-5″), 105.35 (C-9), 98.74 (C-1′), 87.98 (C-1), 85.82 (C-2), 76.55 (C-3′), 75.92 (C-5′), 73.69 (C-2′), 69.62 (C-11), 68.52 (C-4′), 63.49 (C-7), 63.30 (C-8), 61.64 (C-6′), 48.87 (C-3), 46.99 (C-5), 31.48 (C-12), 26.62 (C-6), 20.74 (C-10), 19.29 (C-13), 13.90 (C-14). ESI-HRMS: *m/z* calcd for C_27_H_36_O_11_Na [M + Na]^+^ 559.2150, found 559.2146.

**4-Oxo-9-*O*-butyldebenzoylpaeoniflorin (27):** white solid, 17.8 mg (37.4%, CH_2_Cl_2_: CH_3_OH, 15:1), *R_f_* 0.06 (CH_2_Cl_2_: CH_3_OH, 7:1), mp 125.9 °C, ^1^H NMR (400 MHz, Acetone-*d*_6_) δ 5.18 (s, 1H, H-9), 4.76 (d, *J* = 7.7 Hz, 1H, H-8), 4.02 (d, *J* = 12.5 Hz, 1H, H-6′), 3.81 (dd, *J* = 11.7, 2.6 Hz, 1H, H-11), 3.72 (d, *J* = 12.6 Hz, 1H, H-6′), 3.66–3.59 (m, 2H, H-11, H-5′), 3.49–3.44 (m, 1H, H-3′), 3.39–3.33 (m, 2H, H-2′, H-4′), 3.32–3.25 (m, 2H, H-8,H-1′), 2.89 (dd, *J* = 11.1, 7.6 Hz, 1H, H-5), 2.74 (d, *J* = 16.7 Hz, 1H, H-6), 2.49 (d, *J* = 7.4 Hz, 1H, H-3), 2.38 (d, *J* = 17.9 Hz, 1H, H-3), 2.09 (s, 1H, H-6), 1.47–1.39 (m, 2H, H-12), 1.34 (s, 3H, H-10), 1.33–1.28 (m, 2H, H-13), 0.87 (t, *J* = 7.3 Hz, 3H, H-14). ^13^C NMR (101 MHz, Acetone-*d*_6_) δ 205.30 (C-4), 104.11 (C-9), 98.88 (C-1′), 88.19 (C-1), 86.95 (C-2), 78.17 (C-3′), 77.49 (C-5′), 74.50 (C-2′), 71.72 (C-11), 68.59 (C-4′), 65.33 (C-7), 62.93 (C-6′), 60.19 (C-8), 49.52 (C-3), 47.19 (C-5), 32.32 (C-12), 26.65 (C-6), 20.77 (C-10), 19.82 (C-13), 14.10 (C-14). ESI-HRMS: *m/z* calcd for C_20_H_32_O_10_Na [M + Na]^+^ 455.1888, found 455.1990.

**2,3,4,6-Tetra-*O*-acetyl-4-*O*-sec-butylpaeoniflorin (28):** white solid, 68 mg (37.0%, petroleum: ethyl acetate, 4:1), mp 110.9 °C, *R_f_* 0.40 (petroleum: ethyl acetate, 1:1), 1H NMR (400 MHz, Chloroform-*d*) δ 8.03 (d, *J* = 8.1 Hz, 2H, H-2″, H-6″), 7.60 (t, *J* = 7.4 Hz, 1H, H-4″), 7.48 (t, *J* = 7.7 Hz, 2H, H-3″, H-5″), 5.42 (s, 1H, H-9), 5.11–4.95 (m, 3H, H-2′, H-3′, H-4′), 4.74 (d, *J* = 7.7 Hz, 1H, H-1′), 4.59 (d, *J* = 11.9 Hz, 1H, H-8), 4.49 (d, *J* = 11.9 Hz, 1H, H-8), 4.19–4.08 (m, 2H, H-6′), 3.85 (dq, *J* = 12.1, 6.1 Hz, 1H, H-11), 3.59 (dq, *J* = 8.1, 5.2, 4.2 Hz, 1H, H-5′), 2.73 (t, *J* = 5.9 Hz, 1H, H-5), 2.32 (dt, *J* = 12.0, 6.4 Hz, 1H, H-6), 2.07, 2.02, 2.01, 1.97 (14H, 4COCH_3_, H-3), 1.80 (dd, *J* = 10.6, 4.0 Hz, 1H, H-6), 1.57–1.48 (m, 1H, H-12), 1.43 (dt, *J* = 14.4, 7.3 Hz, 1H, H-12), 1.34 (s, 3H, H-10), 1.19–1.14 (m, 3H, H-13), 0.86 (q, *J* = 7.1 Hz, 3H, H-14). ^13^C NMR (101 MHz, Chloroform-*d*) δ 170.56, 170.34, 169.52, 169.42 (4CH_3_CO), 166.51 (C-7″), 133.57 (C-4″), 129.74 (C-2″, C-6″), 128.77 (C-1″, C-3″, C-5″), 107.91, 107.82 (C-4), 101.24, 101.08 (C-9), 96.48 (C-1′), 88.42 (C-1), 85.71 (C-2), 73.11 (C-3′), 72.42, 72.27 (C-11), 71.86 (C-5′), 71.43, 71.41 (C-2′), 69.67, 69.50 (C-7), 68.55 (C-4′), 62.14 (C-6′), 60.37 (C-8), 42.14 (C-3), 41.48 (C-5), 30.70, 30.65 (C-12), 22.42 (C-6), 21.84, 21.67 (C-13), 20.83, 20.72, 20.69, 20.68 (4CH_3_CO), 19.29 (C-10), 10.23, 10.04 (C-14). ESI-HRMS: *m/z* calcd for C_35_H_44_O_15_Na [M + Na]^+^ 727.2573, found 727.2539.

**2,3,4,6-Tetra-*O*-acetyl-4-oxo-9-*O*-sec-butylpaeoniflorin (29):** white solid, 50.7 mg (27.6%, petroleum: ethyl acetate = 3:1). mp 123.7 °C, *R_f_* 0.37 (petroleum: ethyl acetate, 1:1), ^1^H NMR (400 MHz, Chloroform-*d*) δ 8.01 (d, *J* = 8.0 Hz, 2H, H-2″, H-6″), 7.58 (t, *J* = 7.4 Hz, 1H, H-4″), 7.44 (t, *J* = 7.6 Hz, 2H, H-3″, H-5″), 5.21 (d, *J* = 10.7 Hz, 1H, H-9), 5.16 (dt, *J* = 9.4, 4.7 Hz, 1H, H-2′), 5.03 (qd, *J* = 9.4, 2.1 Hz, 2H, H-3′, H-4′), 4.77 (d, *J* = 7.8 Hz, 1H, H-1′), 4.55–4.46 (m, 2H, H-8), 4.20–4.09 (m, 2H, H-6′), 3.69 (dq, *J* = 23.0, 7.1, 6.6 Hz, 2H, H-11, H-5′), 3.03 (d, *J* = 7.0 Hz, 1H, H-5), 2.68 (d, *J* = 12.3 Hz, 3H, H-3, H-6), 2.08, 2.07, 2.05, 2.02, 1.99 (m, 13H, 4COCH_3_, H-3), 1.56–1.40 (m, 2H, H-12), 1.37 (s, 3H, H-10), 1.11 (d, *J* = 6.2 Hz, 1H, H-13), 0.97 (d, *J* = 6.1 Hz, 2H, H-13), 0.83 (t, *J* = 7.5 Hz, 2H, H-14), 0.72 (t, *J* = 7.4 Hz, 1H, H-14). ^13^C NMR (101 MHz, Chloroform-*d*) δ 205.16, 205.08 (C-4), 170.50, 170.34, 169.52, 169.45 (4CH_3_CO), 166.53, 166.49 (C-7″), 133.60 (C-4″), 129.84 (C-2″, C-6″), 129.50 (C-1″), 128.66 (C-3″, C-5″), 103.70, 102.24 (C-9), 96.31 (C-1′), 88.06, 87.94 (C-1), 85.64, 85.59 (C-2), 76.19, 74.89 (C-11), 73.03 (C-2′), 72.06 (C-5′), 71.55 (C-3′), 68.45 (C-4′), 63.00, 62.93 (C-7), 62.47, 62.45 (C-8), 62.13(C-6′), 49.06, 49.03 (C-6), 46.93 (C-5), 29.38, 28.58 (C-12), 26.41, 26.31 (C-3), 20.83, 20.76, 20.74, 20.69 (4CH_3_CO), 20.58 (C-10), 19.77, 18.04 (C-13), 9.52, 9.34 (C-14). ESI-HRMS: *m/z* calcd for C_35_H_44_O_15_Na [M + Na]^+^ 727.2573, found 727.2544.

**4-*O*-sec-butylpaeoniflorin (30):** white solid, 31.8 mg (49.4%, CH_2_Cl_2_: CH_3_OH, 23:1), *R_f_* 0.36 (CH_2_Cl_2_: CH_3_OH, 7:1), mp 117.2 °C, ^1^H NMR (400 MHz, Chloroform-*d*) δ 7.93 (d, *J* = 8.5 Hz, 2H, H-2″, H-6″), 7.48 (t, *J* = 7.5 Hz, 1H, H-4″), 7.34 (t, *J* = 6.9 Hz, 2H, H-3″, H-5″), 5.45 (s, 1H, H-9), 4.65 (q, *J* = 12.1 Hz, 2H, H-8, H-1′), 4.49 (d, *J* = 7.5 Hz, 1H, H-8), 3.85–3.73 (m, 3H, H-6′, H-11), 3.61 (m, 2H, H-3′, H-4′), 3.46–3.38 (m, 1H, H-2′), 3.28 (d, *J* = 8.2 Hz, 1H, H-5′), 2.63 (d, *J* = 6.4 Hz, 1H, H-6), 2.35 (t, *J* = 7.9 Hz, 1H, H-5), 2.01–1.88 (m, 2H, H-3), 1.76 (d, *J* = 10.7 Hz, 1H, H-6), 1.53–1.37 (m, 2H, H-12), 1.31 (s, 3H, H-10), 1.11 (d, *J* = 6.1 Hz, 3H, H-13), 0.82 (q, *J* = 5.7 Hz, 3H, H-14). ^13^C NMR (101 MHz, Chloroform-*d*) δ 167.07 (C-7″), 133.42 (C-4″), 129.82 (C-2″, C-6″), 129.69 (C-1″), 128.61 (C-3″, C-5″), 107.92, 107.88 (C-4), 101.23, 101.14 (C-9), 98.89 (C-1′), 88.40, 88.37 (C-1), 85.78, 85.74 (C-2), 76.33 (C-3′), 75.73 (C-5′), 73.58 (C-2′), 72.09, 72.03 (C-11), 69.96, 69.80 (C-7), 69.65 (C-4′), 61.68 (C-6′), 60.93 (C-8), 42.64, 42.31 (C-3), 41.33, 40.89 (C-5), 30.67, 30.63 (C-12), 22.75 (C-6), 21.83, 21.57 (C-13), 19.53 (C-10), 10.19, 10.03 (C-14). ESI-HRMS: *m/z* calcd for C_27_H_36_O_11_Na [M + Na]^+^ 559.2150, found 559.2239.

**4-*O*-sec-butyldebenzoylpaeoniflorin (31):** colorless syrup, 20.4 mg (47.2%, CH_2_Cl_2_: CH_3_OH, 16:1), *R_f_* 0.06 (CH_2_Cl_2_: CH_3_OH, 7:1), ^1^H NMR (400 MHz, Acetone-*d*_6_) δ 5.20 (d, *J* = 3.1 Hz, 1H, H-9), 4.66 (d, *J* = 7.7 Hz, 1H, H-8), 3.99 (d, *J* = 12.4 Hz, 1H, H-8), 3.89 (d, *J* = 8.3 Hz, 1H,H-1′), 3.80 (d, *J* = 11.5 Hz, 1H, H-6′), 3.62 (dd, *J* = 12.1, 4.3 Hz, 1H, H-6′), 3.52 (d, *J* = 7.2 Hz, 1H, H-11), 3.45 (d, *J* = 8.4 Hz, 1H, H-5′), 3.33 (d, *J* = 5.3 Hz, 2H, H-3′, H-4′), 3.24 (t, *J* = 8.3 Hz, 1H, H-2′), 2.51–2.37 (m, 2H, H-6, H-5), 2.08 (d, *J* = 5.0 Hz, 2H, H-3), 1.82 (d, *J* = 10.3 Hz, 1H, H-6), 1.43 (m, 2H, H-12), 1.29 (s, 3H, H-10), 1.10 (dd, *J* = 6.1, 2.9 Hz, 3H, H-13), 0.85 (td, *J* = 7.5, 3.2 Hz, 3H, H-14). ^13^C NMR (101 MHz, Acetone-*d*_6_) δ 128.71, 108.66 (C-4), 102.05, 101.80 (C-9), 99.49 (C-1′), 88.94 (C-1), 86.08 (C-2), 78.08 (C-3′), 77.44 (C-5′), 74.71 (C-2′), 72.41, 72.33 (C-11), 71.80, 71.72, 71.63 (C-7), 62.95 (C-4′), 59.15 (C-6′), 59.04 (C-8), 43.46, 43.05 (C-3), 42.29, 41.48 (C-5), 31.33, 31.25 (C-12), 23.18 (C-6), 22.19, 22.02 (C-13), 19.69 (C-10), 10.29, 10.14 (C-14). ESI-HRMS: *m/z* calcd for C_20_H_32_O_10_Na [M + Na]^+^ 455.47, found 455.19.

**4-oxo-9-*O*-sec-butylpaeoniflorin (32):** colorless syrup, 27.5 mg (46.6%, CH_2_Cl_2_: CH_3_OH, 22:1), *R_f_* 0.37 (CH_2_Cl_2_: CH_3_OH, 7:1), ^1^H NMR (400 MHz, Chloroform-*d*) δ 7.92 (d, *J* = 8.0 Hz, 2H, H-2″, H-6″), 7.50 (t, *J* = 7.5 Hz, 1H, H-4″), 7.34 (t, *J* = 7.7 Hz, 2H, H-3″, H-5″), 5.22 (d, *J* = 13.5 Hz, 1H, H-9), 4.77 (t, *J* = 12.3 Hz, 1H, H-8), 4.56 (d, *J* = 8.0 Hz, 2H, H-8, H-1′), 3.85 (t, *J* = 13.4 Hz, 2H, H-6′), 3.64 (m, 3H, H-3′, H-4′, H-11), 3.49 (s, 1H, H-2′), 3.36 (s, 1H, H-5′), 2.96 (d, *J* = 7.1 Hz, 1H, H-6), 2.79 (t,*J* = 9.4 Hz, 1H, H-5), 2.68–2.55 (m, 2H, H-3), 1.99 (d, *J* = 11.8 Hz, 1H, H-6), 1.47–1.32 (m, 5H, H-10, H-12), 1.06 (d, *J* = 6.2 Hz, 1H, H-13), 0.86 (d, *J* = 6.1 Hz, 2H, H-13), 0.78 (t, *J* = 7.4 Hz, 2H, H-14), 0.63 (t, *J* = 7.4 Hz, 1H, H-14). ^13^C NMR (101 MHz, Chloroform-*d*) δ 205.47 (C-4), 167.39 (C-7″), 133.74 (C-4″), 129.91 (C-2″, C-6″), 129.31 (C-1″), 128.63 (C-3″, C-5″), 104.19, 102.66 (C-9), 98.72 (C-1′), 88.01, 87.94 (C-1), 85.66, 85.57 (C-2), 76.50, 76.17 (C-11), 75.93 (C-3′), 74.75 (C-5′), 73.67 (C-2′), 69.70 (C-4′), 63.15(C-7), 63.11 (C-8), 61.68 (C-6′), 48.97 (C-6), 46.99 (C-5), 29.83, 29.33 (C-12), 28.59 (C-3), 20.80 (C-10), 19.74, 17.95 (C-13), 9.43, 9.23 (C-14). ESI-HRMS: *m/z* calcd for C_27_H_36_O_11_Na [M + Na]^+^ 558.2150, found 559.2246.

**4-oxo-9-*O*-sec-butyldebenzoylpaeoniflorin (33):** white solid, 17.5 mg (36.8%, CH_2_Cl_2_: CH_3_OH, 15:1), mp 98.2 °C, *R_f_* 0.05 (CH_2_Cl_2_: CH_3_OH, 7:1), ^1^H NMR (400 MHz, Acetone-*d*_6_) δ 5.33 (d, *J* = 16.3 Hz, 1H, H-9), 4.76 (d, *J* = 9.5 Hz, 1H, H-8), 4.02 (dd, *J* = 12.5, 5.9 Hz, 1H, H-6′), 3.81 (d, *J* = 9.3 Hz, 1H, H-11), 3.73–3.59 (m, 3H, H-6′, H-5′, H-4′), 3.46 (t, *J* = 8.8 Hz, 1H, H-3′), 3.32 (m, 3H, H-2′, H-1′, H-8), 2.89 (t, *J* = 11.0 Hz, 1H, H-5), 2.74 (dd, *J* = 17.7, 3.2 Hz, 1H, H-6), 2.43 (dd, *J* = 17.4, 4.3 Hz, 2H, H-3), 2.08 (d, *J* = 3.9 Hz, 1H, H-6), 1.41 (m, 2H, H-12), 1.34 (s, 3H, H-10), 1.05 (dd, *J* = 21.8, 6.2 Hz, 3H, H-13), 0.83 (q, *J* = 7.7 Hz, 3H, H-14). ^13^C NMR (101 MHz, Acetone-*d*_6_) δ 205.62, 205.44 (C-4), 103.33, 101.47 (C-9), 98.88 (C-1′), 88.20, 87.95 (C-1), 86.89 (C-2), 78.22, 77.50 (C-11), 76.37 (C-3′), 75.06 (C-5′), 74.53 (C-2′), 71.74 (C-4′), 65.32, 65.15 (C-7), 62.96 (C-6′), 60.23, 60.04 (C-8), 59.64 (C-3), 49.68, 49.62 (C-5), 47.34, 47.23 (C-12), 26.81, 26.71 (C-6), 20.87 (C-10), 20.56, 18.80 (C-13), 10.01, 9.64 (C-14). ESI-HRMS: *m/z* calcd for C_20_H_32_O_10_Na [M + Na]^+^ 455.1888, found 455.1910.

**2,3,4,6-Tetra-*O*-acetyl-4-*O*-benzylpaeoniflorin (34):** white solid, 25.2 mg (22.9%, petroleum: ethyl acetate, 6:1) *R_f_* 0.40 (petroleum: ethyl acetate, 1.5:1), mp 172.4 °C, ^1^H NMR (400 MHz, Chloroform-*d*) δ 8.04 (d, *J* = 7.0 Hz, 2H, H-2″, H-6″), 7.61 (t, *J* = 7.4 Hz, 1H, H-4″), 7.49 (d, *J* = 7.9 Hz, 2H, H-3″, H-5″), 7.34–7.26 (m, 5H, H-2″′, H-3″′, H-4″′, H-5′″, H-6″′), 5.50 (s, 1H, H-9), 5.12–4.97 (m, 3H, H-2′, H-3′, H-4′), 4.75 (d, *J* = 7.8 Hz, 1H, H-1′), 4.71 (s, 2H, H-11), 4.61 (d, *J* = 12.0 Hz, 1H, H-8), 4.50 (d, *J* = 11.9 Hz, 1H, H-8), 4.18 (dd, *J* = 12.2, 2.6 Hz, 1H, H-6′), 4.11 (dd, *J* = 12.2, 5.4 Hz, 1H, H-6′), 3.64–3.58 (m, 1H, H-5′), 2.85 (d, *J* = 6.8 Hz, 1H, H-5), 2.33 (dd, *J* = 10.8, 6.9 Hz, 1H, H-6), 2.16 (d, *J* = 10.6 Hz, 1H, H-3), 2.08–1.97 (m, 13H, 4COCH_3_, H-3), 1.82 (d, *J* = 10.6 Hz, 1H, H-6), 1.37 (s, 3H, H-10). ^13^C NMR (101 MHz, Chloroform-*d*) δ 170.56, 170.35, 169.53, 169.44 (4CH_3_CO), 166.53 (C-7″), 137.81 (C-1″′), 133.61 (C-4″), 129.77 (C-2″, C-6″), 129.67 (C-1″), 128.80 (C-3″, C-5″), 128.61 (C-3″′, C-5″′), 127.93 (C-4″′), 127.73 (C-2″′, C-6″′), 107.83 (C-4), 101.31 (C-9), 96.49 (C-1′), 88.46 (C-1), 85.77 (C-2), 73.09 (C-3′), 71.90 (C-5′), 71.43 (C-2′), 70.01 (C-7), 68.53 (C-4′), 66.20 (C-11), 62.12 (C-6′), 60.22 (C-8), 41.93 (C-3), 40.98 (C-5), 22.36 (C-6), 20.87, 20.72, 20.70×2 (4CH_3_CO), 19.26 (C-10). ESI-HRMS: *m/z* calcd for C_38_H_42_O_15_Na [M + Na]^+^ 761.2416, found 761.2439.

**2,3,4,6-Tetra-*O*-acetyl-4-oxo-9-*O*-benzylpaeoniflorin (35):** white solid, 55.7 mg (53.4%, petroleum: ethyl acetate, 4:1). *R_f_* 0.30 (petroleum: ethyl acetate, 1.5:1), mp 140.4 °C, ^1^H NMR (400 MHz, Chloroform-*d*) δ 7.94 (d, *J* = 7.8 Hz, 2H, H-2″, H-6″), 7.58 (t, *J* = 7.5 Hz, 1H, H-4″), 7.38 (t, *J* = 7.7 Hz, 2H, H-3″, H-5″), 7.23 (m, 5H, H-2″′, H-3″′, H-4″′, H-5′″, H-6″′), 5.25–5.16 (m, 2H, H-9, H-2′), 5.06 (q, *J* = 9.0, 8.2 Hz, 2H, H-3′, H-4′), 4.83–4.75 (m, 2H, H-1′, H-8), 4.56–4.45 (m, 3H, H-8, H-11), 4.23–4.12 (m, 2H, H-6′), 3.71–3.64 (m, 1H, H-5′), 3.11 (d, *J* = 7.4 Hz, 1H, H-5), 2.72 (s, 2H, H-6, H-3), 2.10–1.97 (m, 14H, 4COCH_3_, H-6, H-3), 1.44 (s, 3H, H-10). ^13^C NMR (101 MHz, Chloroform-*d*) δ 204.87 (C-4), 170.52, 170.36, 169.54, 169.48 (4CH_3_CO), 166.50 (C-7″), 137.04 (C-1″′), 133.56 (C-4″), 129.86 (C-1″, C-2″, C-6″), 128.66 (C-3″, C-5″), 128.43 (C-3″′, C-5″′), 127.77 (C-2″′, C-4″′, C-6″′), 104.13 (C-9), 96.33 (C-1′), 88.06 (C-1), 86.22 (C-2), 73.01 (C-2′), 72.11 (C-5′), 71.54 (C-3′), 70.26 (C-8), 68.44 (C-4′), 63.14 (C-7), 62.38 (C-11), 62.12 (C-6′), 48.97 (C-5), 46.96 (C-3), 26.31 (C-6), 20.83, 20.76, 20.71×2 (4CH_3_CO), 20.53 (C-10). ESI-HRMS: *m/z* calcd for C_38_H_42_O_15_Na [M + Na]^+^ 761.2416, found 761.2444.

**4-*O*-benzyldebenzoylpaeoniflorin (36):** white solid, 15.3 mg (32.8%, CH_2_Cl_2_: CH_3_OH, 22:1), *R_f_* 0.08 (CH_2_Cl_2_: CH_3_OH, 7:1), mp 214.7 °C, ^1^H NMR (400 MHz, Acetone-*d*_6_) δ 7.39–7.23 (m, 5H, H-2″′, H-3″′, H-4″′, H-5″′, H-6″′), 5.28 (s, 1H, H-9), 4.73–4.64 (m, 3H, H-8, H-1′), 4.03 (d, *J* = 12.3 Hz, 1H, H-11), 3.93 (d, *J* = 12.3 Hz, 1H, H-11), 3.82 (d, *J* = 11.7 Hz, 1H, H-6′), 3.63 (dd, *J* = 12.0, 4.2 Hz, 1H, H-6′), 3.46 (t, *J* = 8.4 Hz, 1H, H-3′), 3.34 (d, *J* = 4.7 Hz, 2H, H-4′, H-5′), 3.24 (t, *J* = 9.2 Hz, 1H, H-2′), 2.61 (d, *J* = 8.6 Hz, 1H, H-6), 2.44 (dd, *J* = 10.7, 6.9 Hz, 1H, H-5), 2.13 (d, *J* = 12.2 Hz, 1H, H-3), 1.95 (d, *J* = 12.2 Hz, 1H, H-3), 1.87 (d, *J* = 10.7 Hz, 1H, H-6), 1.32 (s, 3H, H-10). ^13^C NMR (101 MHz, Acetone-*d*_6_) δ 139.66 (C-1″′), 129.03 (C-3″′, C-5″′), 128.23 (C-2″′, C-6″′), 128.13 (C-4″′), 108.66 (C-4), 102.08 (C-9), 99.47 (C-1′), 88.96 (C-1), 86.21 (C-2), 78.18 (C-3′), 77.44 (C-5′), 74.80 (C-2′), 72.84 (C-7), 71.78 (C-4′), 66.02 (C-11), 62.99 (C-6′), 59.07 (C-8), 42.82 (C-3), 41.07 (C-5), 23.25 (C-6), 19.64 (C-10). ESI-HRMS: *m/z* calcd for C_23_H_30_O_10_Na [M + Na]^+^ 489.1732, found 489.1752.

**4-Oxo-9-*O*-benzylpaeoniflorin (37):** colorless syrup, 29.0 mg (46.2%, CH_2_Cl_2_: CH_3_OH, 23:1), *R_f_* 0.35 (CH_2_Cl_2_: CH_3_OH, 7:1), ^1^H NMR (400 MHz, Chloroform-*d*) δ 7.76 (d, *J* = 7.7 Hz, 2H, H-2″, H-6″), 7.40 (t, *J* = 7.5 Hz, 1H, H-4″), 7.13 (m, 7H, H-3″, H-5″, H-2″′, H-3″′, H-4″′, H-5″′, H-6″′), 5.20 (s, 1H, H-9), 4.69 (dd, *J* = 12.1, 6.8 Hz, 2H, H-11), 4.55 (d, *J* = 11.2 Hz, 2H, H-8, H-1′), 4.33 (d, *J* = 12.1 Hz, 1H, H-8), 3.83 (s, 2H, H-6′), 3.66 (s, 2H, H-3′, H-4′), 3.49 (s, 1H, H-2′), 3.38 (s, 1H, H-5′), 3.00 (d, *J* = 6.8 Hz, 1H, H-6), 2.81 (s, 1H, H-5), 2.72–2.55 (q, *J* = 22.3 Hz, 2H, H-3), 2.02 (d, *J* = 10.6 Hz, 1H, H-6), 1.41 (s, 3H, H-10). ^13^C NMR (101 MHz, Chloroform-*d*) δ 205.40 (C-4), 167.35 (C-7′′), 137.17 (C-1″′), 133.62 (C-4″), 129.84 (C-2″′, C-6″′), 129.16 (C-4″′), 128.59 (C-3″′, C-5″′), 128.32 (C-3″, C-5″), 127.56 (C-1″), 127.42 (C-2″, 6″), 104.95 (C-9), 98.69 (C-1′), 87.98 (C-1), 86.33 (C-2), 76.49 (C-2′), 75.92 (C-5′), 73.72 (C-3′), 70.34 (C-8), 69.71 (C-4′), 63.57 (C-7), 63.27 (C-11), 61.59 (C-6′), 48.90 (C-5), 47.06 (C-3), 26.62 (C-6), 20.72 (C-10). ESI-HRMS: *m/z* calcd for C_30_H_34_O_11_Na [M + Na]^+^ 593.1994, found 593.2039.

**4-Oxo-9-*O*-benzyldebenzoylpaeoniflorin (38):** white solid, 18.8 mg (36.6%, CH_2_Cl_2_: CH_3_OH, 16:1), *R_f_* 0.08 (CH_2_Cl_2_: CH_3_OH, 7:1), mp 169.4 °C, ^1^H NMR (400 MHz, Acetone-*d*_6_) δ 7.51–7.37 (m, 5H, H-2″′, H-3″′, H-4″′, H-5″′, H-6″′), 5.50 (s, 1H, H-9), 4.64 (d, *J* = 12.2 Hz, 1H, H-8), 4.20 (d, *J* = 12.5 Hz, 1H, H-6′), 3.99 (dd, *J* = 11.7, 2.6 Hz, 1H, H-11), 3.91 (d, *J* = 12.5 Hz, 1H, H-6′), 3.79 (dd, *J* = 11.7, 5.6 Hz, 1H, H-11), 3.70–3.62 (m, 2H, H-4′, H-5′), 3.50 (m, 4H, H-2′, H-3′, H-8, H-1′), 3.10 (dd, *J* = 11.1, 7.5 Hz, 1H, H-5), 2.97 (d, *J* = 18.0 Hz, 1H, H-6), 2.73 (d, *J* = 7.4 Hz, 1H, H-3), 2.64 (d, *J* = 18.0 Hz, 1H, H-6), 2.27 (d, *J* = 11.0 Hz, 1H, H-3), 1.55 (s, 3H, H-10). ^13^C NMR (101 MHz, Acetone-*d*_6_) δ 205.58 (C-4), 139.16 (C-1″′), 128.92 (C-3″′, C-5″′), 128.25 (C-2″′, C-6″′), 128.02 (C-4″′), 103.88 (C-9), 98.94 (C-1′), 88.32 (C-1), 87.42 (C-2), 78.24 (C-2′), 77.53 (C-5′), 74.58 (C-3′), 71.80 (C-8), 70.63 (C-4′), 65.46 (C-7), 63.01 (C-11), 60.18 (C-6′), 49.59 (C-5), 47.29 (C-3), 26.74 (C-6), 20.78 (C-10). ESI-HRMS: *m/z* calcd for C_23_H_30_O_10_Na [M + Na]^+^ 489.1732, found 489.1763.

**2,3,4,6-Tetra-*O*-acetyl-4-*O*-ethylbenzenepaeoniflorin (39):** white solid, 32.8 mg (30.7%, petroleum: ethyl acetate, 6:1), *R_f_* 0.42 (petroleum: ethyl acetate, 1.5:1), mp 192.3 °C, ^1^H NMR (400 MHz, Chloroform-*d*) δ 8.02 (d, *J* = 8.5 Hz, 2H, H-2″, H-6″), 7.60 (t, *J* = 7.5 Hz, 1H, H-4″), 7.46 (t, *J* = 7.8 Hz, 2H, H-3″, H-5″), 7.26–7.16 (m, 5H, H-2″′, H-3″′, H-4″′, H-5′″, H-6″′), 5.43 (s, 1H, H-9), 5.11–4.96 (m, 3H, H-2′, H-3′, H-4′), 4.73 (d, *J* = 7.8 Hz, 1H, H-1′), 4.58 (d, *J* = 12.0 Hz, 1H, H-8), 4.47 (d, *J* = 12.0 Hz, 1H, H-8), 4.16 (d, *J* = 2.6 Hz, 1H, H-6′), 4.11 (dd, *J* = 12.2, 5.3 Hz, 1H, H-6′), 3.83 (t, *J* = 6.8 Hz, 2H, H-11), 3.63–3.57 (m, 1H, H-5′), 2.88 (t, *J* = 7.7 Hz, 2H, H-12), 2.72 (d, *J* = 8.6 Hz, 1H, H-5), 2.28 (dd, *J* = 10.8, 7.0 Hz, 1H, H-6), 2.06, 2.03, 2.02, 1.98 (m, 13H, 4COCH_3_, H-3), 1.93 (d, *J* = 12.5 Hz, 1H, H-3), 1.75 (d, *J* = 10.7 Hz, 1H, H-6), 1.33 (s, 3H, H-10). ^13^C NMR (101 MHz, Chloroform-*d*) δ 170.54, 170.33, 169.51, 169.41 (4CH_3_CO), 166.49 (C-7″), 138.50 (C-1″′), 133.57 (C-4″), 129.74 (C-2″, C-6″), 129.66 (C-1″), 129.06 (C-3″, C-5″), 128.76 (C-3″′, C-5″′), 128.48 (C-2″′, C-6″′), 126.45 (C-4″′), 107.67 (C-4), 101.21 (C-9), 96.45 (C-1′), 88.44 (C-1), 85.65 (C-2), 73.07 (C-3′), 71.87 (C-5′), 71.41 (C-2′), 69.87 (C-7), 68.52 (C-4′), 64.97 (C-11), 62.10 (C-6′), 60.22 (C-8), 41.66 (C-3), 40.75 (C-5), 36.58 (C-12), 22.31 (C-6), 20.85, 20.70, 20.68×2 (4CH_3_CO), 19.21 (C-10). ESI-HRMS: *m/z* calcd for C_39_H_44_O_15_Na [M + Na]^+^ 775.2573, found 775.2615.

**2,3,4,6-Tetra-*O*-acetyl-4-oxo-9-*O*-ethylbenzenepaeoniflorin (40):** colorless syrup, 50.8 mg (47.6%, petroleum: ethyl acetate, 4:1), *R_f_* 0.34 (petroleum: ethyl acetate, 1.5:1), ^1^H NMR (400 MHz, Chloroform-*d*) δ 7.96 (d, *J* = 6.9 Hz, 2H, H-2″, H-6″), 7.62–7.55 (m, 1H, H-4″), 7.43 (t, *J* = 7.8 Hz, 2H, H-3″, H-5″), 7.25–7.18 (m, 2H, H-2″′, H-3″′), 7.18–7.10 (m, 3H, H-4″′, H-5′″, H-6″′), 5.16 (t, *J* = 9.4 Hz, 1H, H-2′), 5.10 (s, 1H, H-9), 5.07–4.99 (m, 2H, H-3′, H-4′), 4.77 (d, *J* = 7.8 Hz, 1H, H-1′), 4.53–4.44 (m, 2H, H-8), 4.19 (dd, *J* = 12.2, 2.6 Hz, 1H, H-6′), 4.12 (dd, *J* = 12.3, 5.5 Hz, 1H, H-6′), 3.89 (t, *J* = 7.2 Hz, 1H, H-11), 3.67–3.57 (m, 2H, H-11, H-5′), 3.06 (d, *J* = 7.4 Hz, 1H, H-5), 2.78 (t, *J* = 7.4 Hz, 2H, H-12), 2.71 (dd, *J* = 10.8, 7.5 Hz, 1H, H-6), 2.63 (d, *J* = 14.7 Hz, 1H, H-3), 2.05, 2.02, 1.99 (m, 14H, 4COCH_3_, H-6, H-3), 1.38 (s, 3H, H-10). ^13^C NMR (101 MHz, Chloroform-*d*) δ 204.94 (C-4), 170.49, 170.33, 169.52, 169.44 (4CH_3_CO), 166.47 (C-7″), 138.50 (C-1″′), 133.60 (C-4″), 129.78 (C-2″, C-6″), 129.49 (C-1″), 129.03 (C-3″, C-5″), 128.75 (C-3″′, C-5″′), 128.43(C-2″′, C-6″′), 126.36 (C-4″′), 104.77 (C-9), 96.32 (C-1′), 87.96 (C-1), 86.02 (C-2), 73.01 (C-2′), 72.08 (C-5′), 71.53 (C-3′), 69.58 (C-8), 68.43 (C-4′), 63.07 (C-7), 62.34 (C-11), 62.10 (C-6′), 48.86 (C-5), 46.98 (C-3), 35.89 (C-12), 26.34 (C-6), 20.82, 20.72, 20.69×2 (4CH_3_CO), 20.48 (C-10). ESI-HRMS: *m/z* calcd for C_39_H_44_O_15_Na [M + Na]^+^ 775.2573, found 775.2655.

**4-*O*-ethylbenzenepaeoniflorin (41):** colorless syrup, 23.6 mg (44.9%, CH_2_Cl_2_: CH_3_OH, 23:1), *R_f_* 0.36 (CH_2_Cl_2_: CH_3_OH, 7:1), ^1^H NMR (400 MHz, Chloroform-*d*) δ 7.89 (d, *J* = 7.7 Hz, 2H, H-2″, H-6″), 7.44 (t, *J* = 7.5 Hz, 1H, H-4″), 7.29 (d, *J* = 7.7 Hz, 2H, H-3″, H-5″), 7.25–7.11 (m, 5H, H-2″′, H-3″′, H-4″′, H-5″′, H-6″′), 5.46 (s, 1H, H-9), 4.63 (q, *J* = 12.1 Hz, 2H, H-8, H-1′), 4.48 (d, *J* = 7.3 Hz, 1H, H-8), 3.83–3.71 (m, 4H, H-6′, H-11), 3.57 (s, 2H, H-3′, H-4′), 3.39 (s, 1H, H-2′), 3.28 (s, 1H, H-5′), 2.84 (t, *J* = 7.4 Hz, 2H, H-12), 2.64 (d, *J* = 6.5 Hz, 1H, H-6), 2.38–2.30 (t, *J* = 11.5 Hz 1H, H-5), 1.92 (s, 2H, H-3), 1.75 (d, *J* = 10.6 Hz, 1H, H-6), 1.31 (s, 3H, H-10). ^13^C NMR (101 MHz, Chloroform-*d*) δ 167.10 (C-7′′), 138.50 (C-1″′), 133.48 (C-4″), 129.81 (C-2″, C-6″), 129.58 (C-1″), 129.05 (C-3″, C-5″), 128.64 (C-3″′, C-5″′), 128.49 (C-2″′, C-6″′), 126.43 (C-4″′), 107.75 (C-4), 101.28 (C-9), 98.92 (C-1′), 88.48 (C-1), 85.71 (C-2), 76.41 (C-3′), 75.72 (C-5′), 73.52 (C-2′), 70.08 (C-7), 69.52 (C-4′), 64.80 (C-11), 61.65 (C-6′), 60.84 (C-8), 41.79 (C-3), 40.24 (C-5), 36.57 (C-12), 22.76 (C-6), 19.51 (C-10). ESI-HRMS: *m/z* calcd for C_31_H_36_O_11_Na [M + Na]^+^ 607.2150, found 607.2206.

**4-*O*-ethylbenzenedebenzoylpaeoniflorin (42):** white solid, 14.7 mg (34.0%, CH_2_Cl_2_: CH_3_OH, 16:1), *R_f_* 0.07 (CH_2_Cl_2_: CH_3_OH, 7:1), mp 168.7 °C, ^1^H NMR (400 MHz, Acetone-*d*_6_) δ 7.30–7.16 (m, 5H, H-2″′, H-3″′, H-4″′, H-5″′, H-6″′), 5.22 (s, 1H, H-9), 4.65 (d, *J* = 7.7 Hz, 1H, H-8), 3.99 (dd, *J* = 12.4, 4.0 Hz, 1H, H-11), 3.89 (dd, *J* = 12.3, 6.4 Hz, 1H, H-11), 3.84–3.76 (m, 3H, H-8, H-1′, H-6′), 3.62 (d, *J* = 10.9 Hz, 1H, H-6′), 3.46–3.42 (m, 1H, H-3′), 3.35–3.30 (m, 2H, H-4′, H-5′), 3.23 (t, *J* = 7.4 Hz, 1H, H-2′), 2.83 (t, *J* = 7.2 Hz, 2H, H-12), 2.50 (d, *J* = 8.7 Hz, 1H, H-6), 2.39 (dd, *J* = 10.7, 6.9 Hz, 1H, H-5), 2.01 (d, *J* = 12.2 Hz, 1H, H-3), 1.85 (dd, *J* = 12.3, 1.9 Hz, 1H, H-3), 1.79 (d, *J* = 10.6 Hz, 1H, H-6), 1.28 (s, 3H, H-10). ^13^C NMR (101 MHz, Acetone-*d*_6_) δ 139.88 (C-1″′), 129.79 (C-3″′, C-5″′), 129.07 (C-2″′, C-6″′), 126.94 (C-4″′), 108.45 (C-4), 101.98 (C-9), 99.44 (C-1′), 88.90 (C-1), 86.09 (C-2), 78.16 (C-3′), 77.43 (C-5′), 74.41 (C-2′), 72.69 (C-7), 71.76 (C-4′), 64.97 (C-11), 62.98 (C-6′), 58.46 (C-8), 42.56 (C-3), 40.93 (C-5), 37.17 (C-12), 23.18 (C-6), 19.61 (C-10). ESI-HRMS: *m/z* calcd for C_24_H_32_O_10_Na [M + Na]^+^ 503.51, found 503.19.

**4-Oxo-9-*O*-ethylbenzenepaeoniflorin (43):** white solid, 30.5 mg (47.4%, CH_2_Cl_2_: CH_3_OH, 22:1), *R_f_* 0.37 (CH_2_Cl_2_: CH_3_OH, 7:1), mp 171.8 °C, ^1^H NMR (400 MHz, Chloroform-*d*) δ 7.82 (d, *J* = 7.8 Hz, 2H, H-2″, H-6″), 7.45 (t, *J* = 7.5 Hz, 1H, H-4″), 7.27 (d, *J* = 15.3 Hz, 2H, H-3″, H-5″), 7.20–7.02 (m, 5H, H-2″′, H-3″′, H-4″′, H-5″′, H-6″′), 5.10 (s, 1H, H-9), 4.71 (d, *J* = 11.7 Hz, 1H, H-8), 4.52 (d, *J* = 11.0 Hz, 2H, H-8, H-1′), 3.86–3.75 (m, 3H, H-6′, H-11), 3.65 (s, 2H, H-3′, H-4′), 3.50–3.43 (m, 2H, H-2′, H-11), 3.35 (s, 1H, H-5′), 2.97 (d, *J* = 6.9 Hz, 1H, H-6), 2.78 (t, *J* = 9.0 Hz, 1H, H-5), 2.69 (t, *J* = 7.4 Hz, 2H H-12), 2.66–2.46 (q, *J* = 27.9 Hz, 2H, H-3), 1.97 (d, *J* = 10.9 Hz, 1H, H-6), 1.36 (s, 3H, H-10). ^13^C NMR (101 MHz, Chloroform-*d*) δ 205.36 (C-4), 167.30 (C-7′′), 138.48 (C-1″′), 133.67 (C-4″), 129.78 (C-2″, C-6″), 129.29 (C-1″), 129.00 (C-3″, C-5″), 128.70 (C-3″′, C-5″′), 128.41 (C-2″′, C-6″′), 126.34 (C-4″′), 105.11 (C-9), 98.71 (C-1′), 88.00 (C-1), 86.00 (C-2), 76.51 (C-2′), 75.91 (C-5′), 73.65 (C-3′), 69.58 (C-8), 69.47 (C-4′), 63.45 (C-7), 63.13 (C-11), 61.58 (C-6′), 48.80 (C-5), 47.01 (C-3), 46.12, 35.84 (C-12), 26.60 (C-6), 20.68 (C-10). ESI-HRMS: *m/z* calcd for C_31_H_36_O_11_Na [M + Na]^+^ 607.2150, found 607.2201.

**4-Oxo-9-*O*-ethylbenzenedebenzoylpaeoniflorin (44):** colorless syrup, 17.4 mg (32.9%, CH_2_Cl_2_: CH_3_OH, 16:1), *R_f_* 0.06 (CH_2_Cl_2_: CH_3_OH, 7:1), ^1^H NMR (400 MHz, Acetone-*d*_6_) δ 7.36–7.12 (m, 5H, H-2″′, H-3″′, H-4″′, H-5″′, H-6″′), 5.22 (s, 1H, H-9), 4.78 (d, *J* = 7.7 Hz, 1H, H-8), 4.01 (d, *J* = 12.5 Hz, 1H, H-6′), 3.89–3.81 (m, 2H, H-11, H-6′), 3.71–3.57 (m, 3H, H-11, H-4′, H-5′), 3.50–3.46 (m, 1H, H-3′), 3.40–3.27 (m, 3H, H-8, H-1′, H-2′), 2.92 (dd, *J* = 11.1, 7.6 Hz, 1H, H-5), 2.78 (q, *J* = 7.4 Hz, 3H, H-6, H-12), 2.53 (d, *J* = 7.5 Hz, 1H, H-3), 2.42 (d, *J* = 17.9 Hz, 1H, H-3), 2.09 (d, *J* = 9.6 Hz, 1H, H-6), 1.36 (s, 3H, H-10). ^13^C NMR (101 MHz, Acetone-*d*_6_) δ 205.45 (C-4), 140.02 (C-1″′), 129.89 (C-3″′, C-5″′), 129.01 (C-2″′, C-6″′), 126.86 (C-4″′), 103.96 (C-9), 98.89 (C-1′), 88.17 (C-1), 87.16 (C-2), 78.27 (C-2′), 77.53 (C-5′), 74.56 (C-3′), 71.78 (C-8), 69.85 (C-4′), 65.30 (C-7), 63.00 (C-11), 60.16 (C-6′), 49.46 (C-5), 47.30 (C-3), 36.48 (C-12), 26.65 (C-6), 20.75 (C-10). ESI-HRMS: *m/z* calcd for C_24_H_32_O_10_Na [M + Na]^+^ 503.1888, found 503.1914.

**4-*O*-t-butylbenzenepaeoniflorin (45):** white solid, 24.1 mg (14.5%, CH_2_Cl_2_: CH_3_OH, 25:1), *R_f_* 0.46 (CH_2_Cl_2_: CH_3_OH, 7:1), mp 81.2 °C, ^1^H NMR (400 MHz, Chloroform-*d*) δ 7.97 (d, *J* = 7.1 Hz, 2H, H-2″, H-6″), 7.51 (t, *J* = 7.6 Hz, 1H, H-4″), 7.37 (t, *J* = 7.7 Hz, 2H, H-3″, H-5″), 5.51 (s, 1H, H-9), 4.73 (d, *J* = 12.0 Hz, 1H, H-8), 4.61 (d, *J* = 12.3 Hz, 1H, H-8), 4.43 (d, *J* = 7.5 Hz, 1H, H-1′), 3.62–3.31 (m, 6H, H-6′, H-5′, H-4′, H-3′, H-2′), 2.59 (d, *J* = 6.3 Hz, 1H, H-6), 2.38–2.31 (m, 1H, H-5), 2.09 (d, *J* = 12.4 Hz, 1H, H-3), 1.88 (dd, *J* = 22.4, 11.5 Hz, 2H, H-3, H-6), 1.33 (s, 3H, H-12), 1.25 (s, 3H, H-14), 1.14 (d, *J* = 9.9 Hz, 6H, H-10, H-13). ^13^C NMR (101 MHz, Chloroform-*d*) δ 167.14 (C-7″), 133.60 (C-4″), 129.90 (C-2″, C-6″), 129.54 (C-1″), 128.70 (C-3″, C-5″), 105.27 (C-4), 100.90 (C-9), 98.52 (C-1′), 88.38 (C-1), 86.00 (C-2), 76.45 (C-3′), 74.11 (C-5′), 73.65 (C-2′), 73.39 (C-4′), 72.33 (C-8), 70.43 (C-11), 62.85 (C-7), 60.45 (C-6′), 42.95 (C-6), 29.71 (C-12, C-3), 27.43 (C-10, C-13), 22.70 (C-5), 19.27 (C-14). ESI-HRMS: *m/z* calcd for C_27_H_36_O_11_Na [M + Na]^+^ 559.2150, found 559.2203.

**4-Oxo-9-*O*-t-butylpaeoniflorin (46):** colorless syrup, 21.3 mg (12.8%, CH_2_Cl_2_: CH_3_OH, 21:1), *R_f_* 0.26 (CH_2_Cl_2_: CH_3_OH, 7:1), ^1^H NMR (400 MHz, Chloroform-*d*) δ 7.93 (d, *J* = 7.3 Hz, 2H, H-2″, H-6″), 7.51 (t, *J* = 7.4 Hz, 1H, H-4″), 7.36 (t, *J* = 7.7 Hz, 2H, H-3″, H-5″), 5.39 (s, 1H, H-9), 4.80 (d, *J* = 11.8 Hz, 1H, H-8), 4.59–4.49 (m, 2H, H-8, H-1′), 3.84 (s, 2H, H-6′), 3.65 (s, 2H, H-3′, H-4′), 3.48 (s, 1H, H-2′), 3.36 (s, 1H, H-5′), 2.95 (d, *J* = 7.1 Hz, 1H, H-6), 2.81–2.74 (m, 1H, H-5), 2.63 (s, 2H, H-3), 1.98 (d, *J* = 10.9 Hz, 1H, H-6), 1.38 (s, 3H, H-14), 1.25 (s, 2H, H-12), 1.07 (s, 7H, H-10, H-13, H-12). ^13^C NMR (101 MHz, Chloroform-*d*) δ 205.88 (C-4), 167.23 (C-7″), 133.71 (C-4″), 129.87 (C-2″, C-6″), 129.36 (C-1″), 128.67 (C-3″, C-5″), 99.98 (C-9),98.67 (C-1′), 87.58 (C-1), 85.43 (C-2), 76.50 (C-2′), 75.91 (C-5′), 73.72 (C-3′), 69.70 (C-4′), 63.50 (C-8), 63.44 (C-7), 61.63 (C-6′), 49.11 (C-3), 46.86 (C-6), 29.84 (C-12), 28.77 (C-10, C-13), 28.56 (C-5), 20.92 (C-14). ESI-HRMS: *m/z* calcd for C_27_H_36_O_11_Na [M + Na]^+^ 559.2150, found 559.2217.

## 4. Conclusions

In summary, Sc(CF_3_SO_3_)_3_ served as the catalyst to initiate the dehydration and rearrangement reactions of paeoniflorin with alcohols. Following this, a sequence of chemical modifications was applied to paeoniflorin, encompassing acetylation, deacetylation, and debenzoylation processes. This comprehensive approach led to the synthesis of 46 paeoniflorin derivatives, and their activities were evaluated. Notably, compounds **3**, **8**, **18**, **20**, **21**, **29**, **34**, and **40** showed more significant inhibitory effects than paeoniflorin on the activated secretion of IL-1*β*. Moreover, compounds **29** and **31** showed superior inhibitory effects on the production of NO in macrophages compared to paeoniflorin, indicating that isobutyl may be the dominant group in inhibiting NO production. Computational research revealed potential binding interactions between the synthesized compounds and target proteins. Overall, the results of this study suggest that paeoniflorin derivatives have potential as anti-inflammatory agents.

## Figures and Tables

**Table 1:** Molecular docking results.

Ligand	Receptor	Docking Score (kcal/mol)	Interactions	Distance (Å)
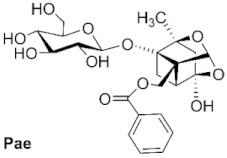	3HR4	−5.74	GLY594 ^a^	1.86; 1.89
			ARG633 ^a^	2.34
			GLU661 ^a^	1.99
			GLN665 ^a^	2.37
			LYS549 ^a^	2.24
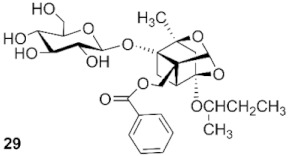	3HR4	−6.24	GLU661 ^a^	1.53; 1.99
			LYS549 ^a^	2.13
			THR592 ^a^	2.36
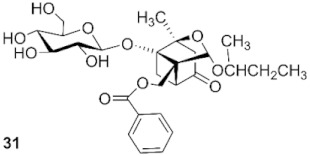	3HR4	−7.49	GLU661 ^a^	1.90
			LYS549 ^a^	1.97
			THR592 ^a^	2.32
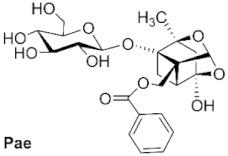	5R8Q	−5.58	GLU25 ^a^	2.00
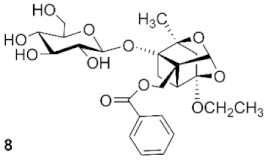	5R8Q	−9.15	GLU25 ^a^	1.96
			LEU26 ^a^	2.79
			TYR24 ^b^	5.20
			LYS74 ^a^	2.06
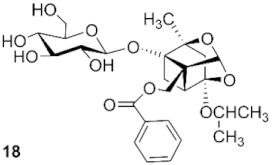	5R8Q	−9.54	GLU25 ^a^	1.86; 1.80
			TYR24 ^b^	4.4
			LYS74 ^a^	1.76

^a^ H-bond; ^b^ Pi-Pi stacking.

## Data Availability

The details of the data supporting the report results in this research, were included in the paper and Supplementary Materials.

## Supplementary Materials

The following supporting information can be downloaded at https://www.mdpi.com/article/10.3390/molecules28196922/s1. Figure S1–S90: ^1^H NMR and ^13^C NMR spectra of the synthesized paeoniflorin derivatives (compounds **1**–**46**); Table S1–S2: Lewis acid and molar ratio screening results; Table S3–S5: single crystal structure and data.

## Sample Availability

Samples of the compounds are available from the authors.
