# Supplementary material for: New Monoterpene Glycoside Paeoniflorin Derivatives as NO and IL-1β Inhibitors: Synthesis and Biological Evaluation
Source: Molecules. 2023 Oct 3;28(19):6922. doi: 10.3390/molecules28196922 (PMC10574144; doi:10.3390/molecules28196922)
Supplement: Supplementary file 1 [file molecules-28-06922-s001.zip › molecules-2578230-supplementary.pdf]

Supporting Information

New Monoterpene Glycoside Paeoniflorin Derivatives as NO and IL-1*β* Inhibitors: Synthesis and Biological Evaluation

Yongjie Chen [a,b,†], Guoqing Zhang [a,†], Dongyi Cao [a,c], Fei Wang [a], Fan Zhang [d], Huawu Shao [a] and Wei Jiao [a,*]

a *Natural Products Research Centre, Chengdu Institute of Biology, Chinese Academy of Sciences, Chengdu 610041, China*

b *Nanchong Central Hospital, Nanchong, 637000, China*

c *University of Chinese Academy of Sciences, Beijing 100049, China*

d *School of Pharmacy, North Sichuan Medical College, Nanchong, 637100, China*

** Correspondence: jiaowei@cib.ac.cn*

† These authors contributed equally to this work.

1. Reaction condition optimization 2
2. ^{1}H NMR and ^{13}C NMR spectra of the synthesized compounds 4
3. Single crystal structure and data 50

1. Reaction condition optimization

Among the screening results of Lewis acids, $Sc(CF_3SO_3)_3$ has the fastest reaction speed and the highest yield. $Sc(CF_3SO_3)_3$ is chosen as the catalyst for the experimental reaction. The screening results of Lewis acid are shown in **Table S1.**

Table S1. Lewis acid screening results

Entry	Solven	Temp. (°C)	Lewis acid	Time (h)	Yield (%)
1	MeOH	65	$NiCl_2$	6	No reaction
2	MeOH	65	Br_2Cu	6	25
3	MeOH	65	$FeCl_3$	5	72
4	MeOH	65	$BiCl_3$	2	86
5	MeOH	65	$BiBr_3$	5	70
6	MeOH	65	$PbCl_2$	2	84
7	MeOH	65	InI_3	8	10
8	MeOH	65	$Yb(CF_3SO_3)_3$	8	15
9	MeOH	65	$Cu(CF_3SO_3)_2$	8	12
**10**	**MeOH**	**65**	$\mathbf{Sc(CF_3SO_3)_3}$	**1**	**90**
11	MeOH	65	$Zn(CF_3SO_3)_2$	8	No reaction
12	MeOH	65	$La(CF_3SO_3)_3$	8	12

The above results are obtained when the molar ratio of paeoniflorin to $Sc(CF_3SO_3)_3$ is 1: 0.5. it is necessary to continue to optimize the molar ratio of paeoniflorin to $Sc(CF_3SO_3)_3$, and select the molar ratios of 1: 0.5, 1: 1, and 1: 1.5 for comparison. The reaction completion time is shortened when the molar ratio is raised to 1: 1. Then the molar ratio is increased to 1:1.5, and the reaction completion time has no significant change compared with the reaction completion time when the molar ratio is 1:1. It was finally determined that the molar ratio of paeoniflorin to Lewis acid was 1:1. The reaction was heated to reflux at the boiling point of the solvent. The screening results of the molar ratio of paeoniflorin to $Sc(CF_3SO_3)_3$ are shown in **Table S2**.

Table S2. Molar ratio screening results

Entry	Proportion	Time (h)	Yield (%)
1	1: 0.5	1.0	90
2	1: 1	0.5	95
3	1: 1.5	0.5	93

2. ^{1}H NMR and ^{13}C NMR spectra of the synthesized compounds

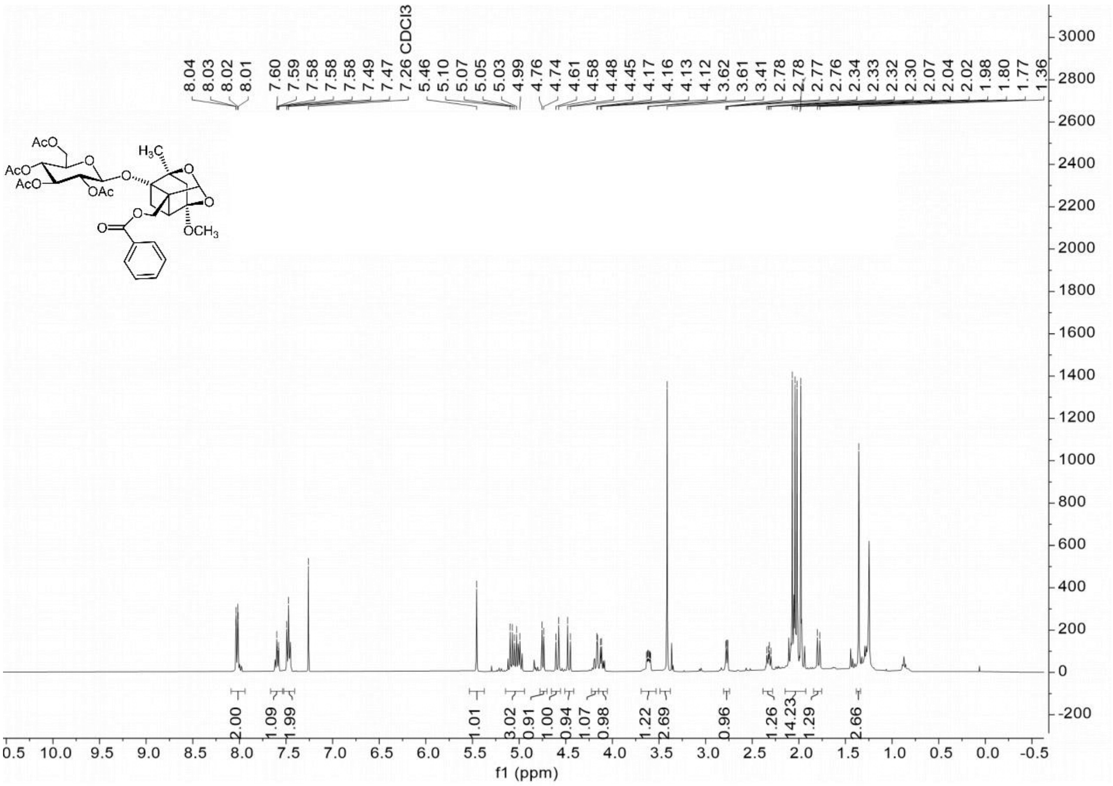

Figure S1. The ^{1}H NMR of compound **1**.

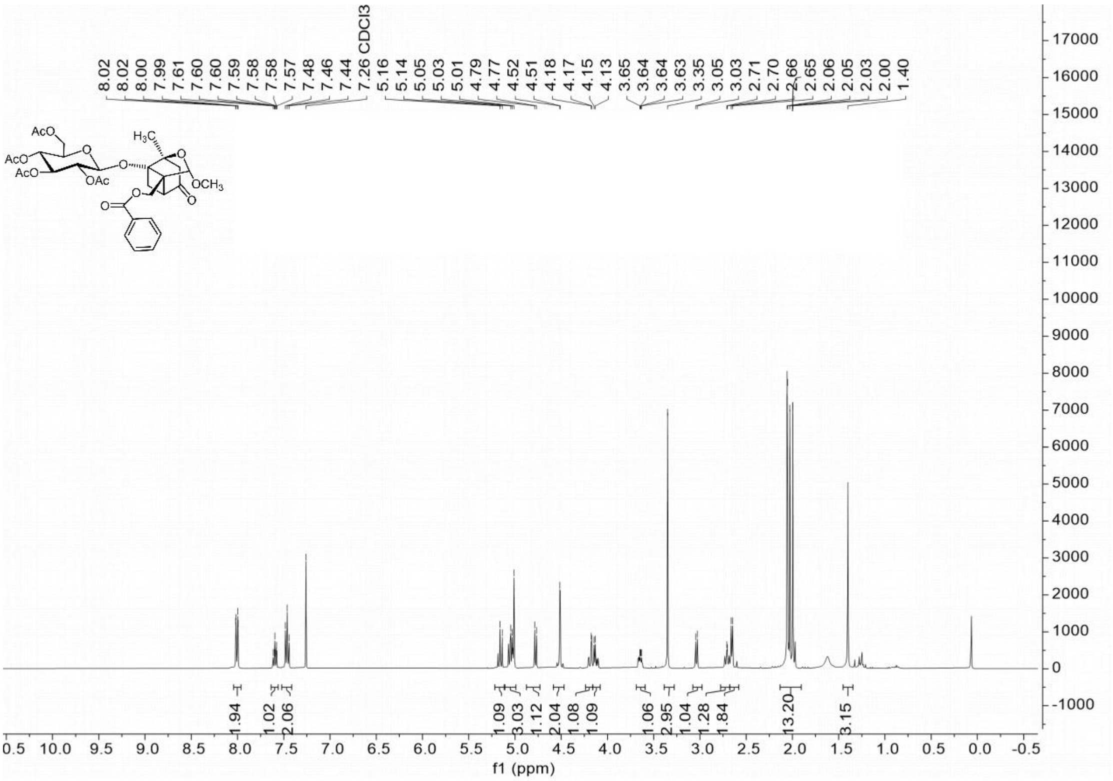

Figure S2. The ^{1}H NMR of compound **2**.

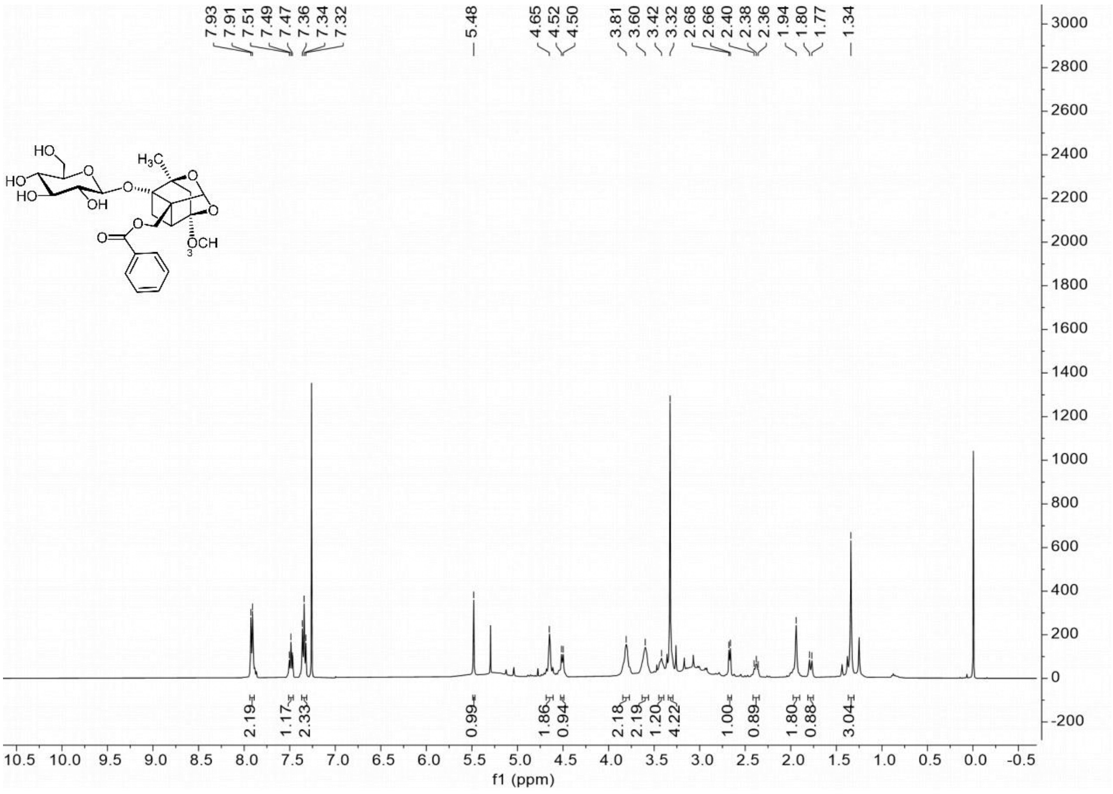

Figure S3. The ^{1}H NMR of compound **3**.

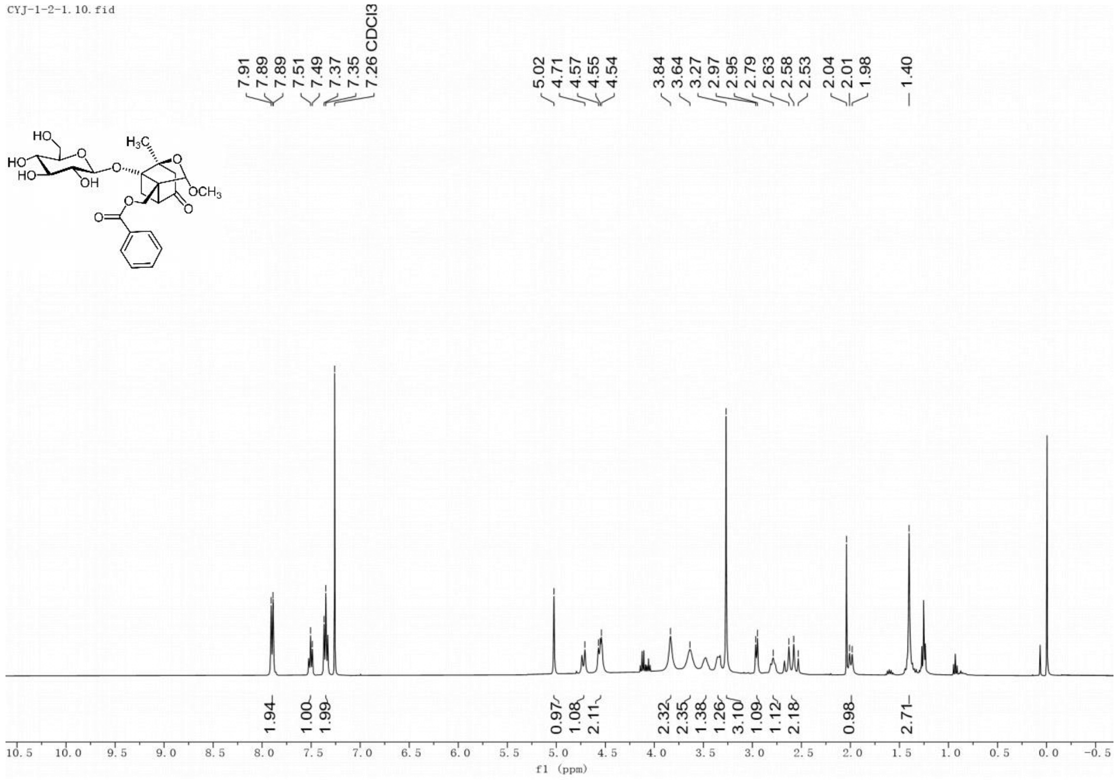

Figure S4. The ^{1}H NMR of compound **4**.

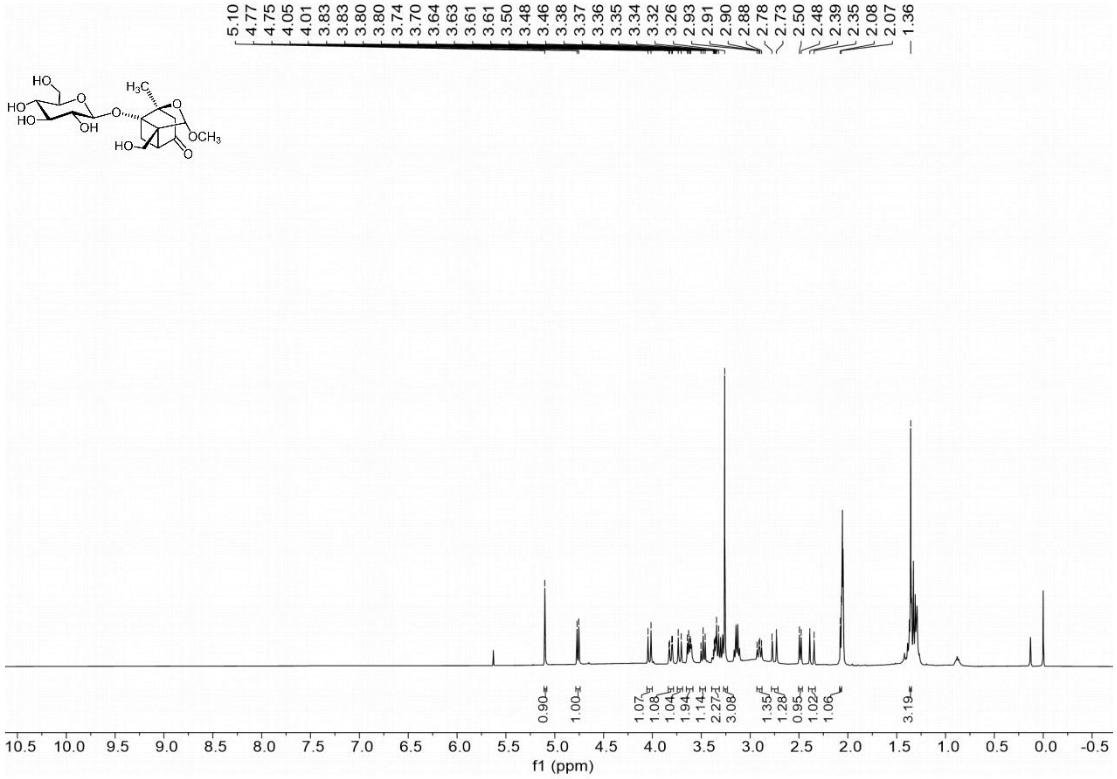

Figure S5. The ^{1}H NMR of compound **5**.

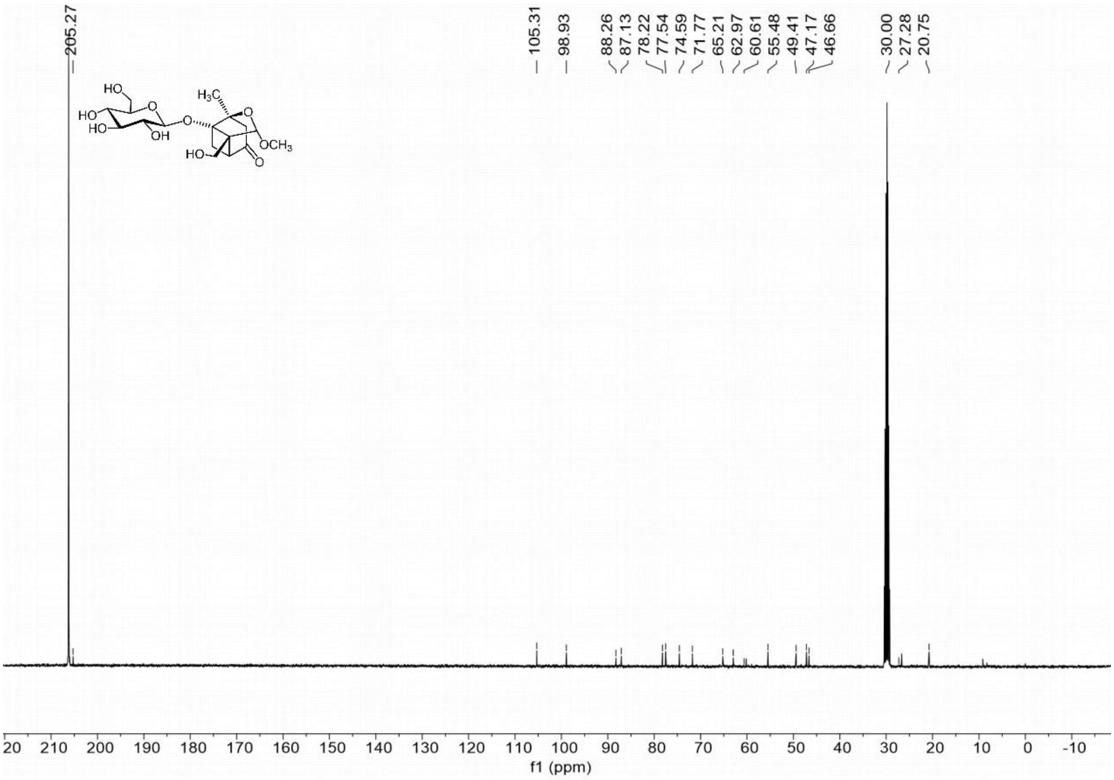

Figure S6. The ^{13}C NMR of compound **5**.

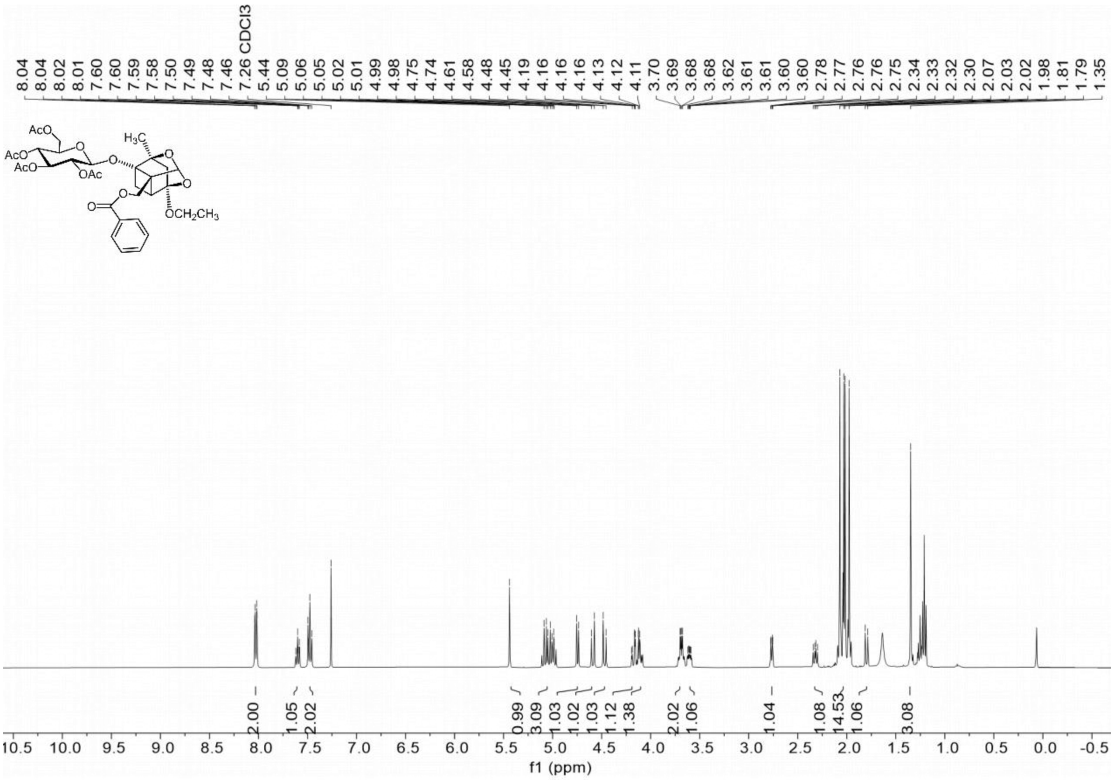

Figure S7. The ^{1}H NMR of compound **6**.

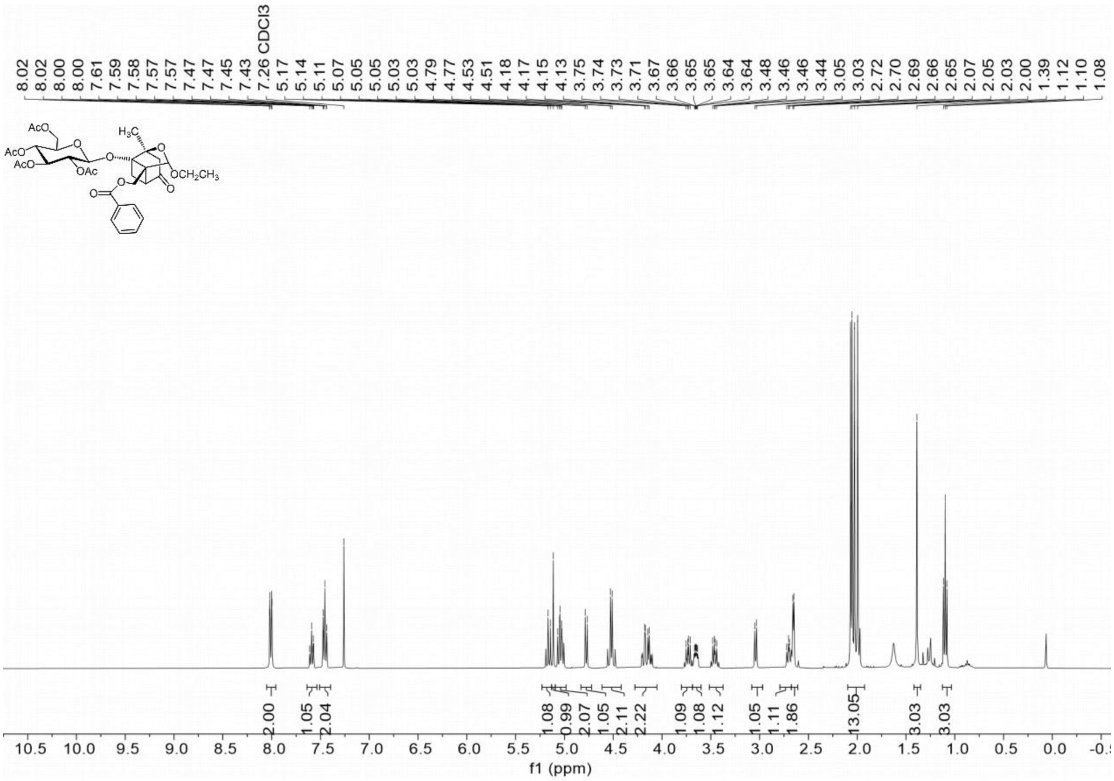

Figure S8. The ^{1}H NMR of compound **7**.

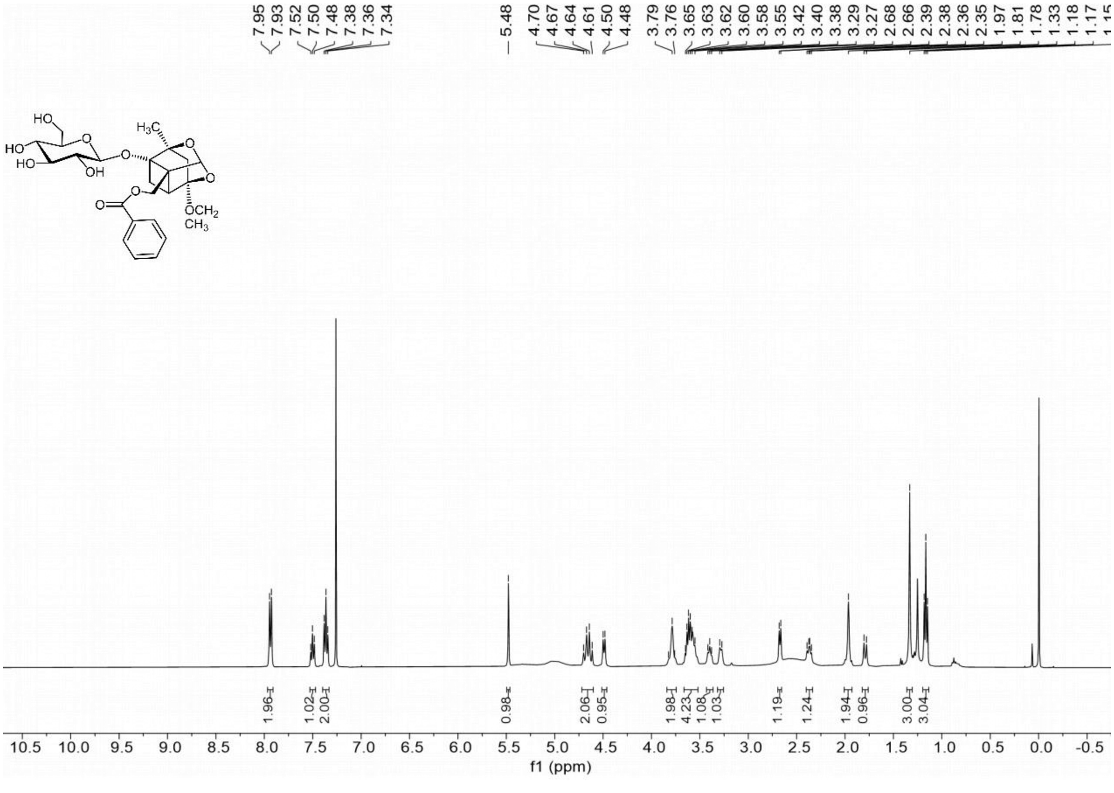

Figure S9. The ^{1}H NMR of compound **8**.

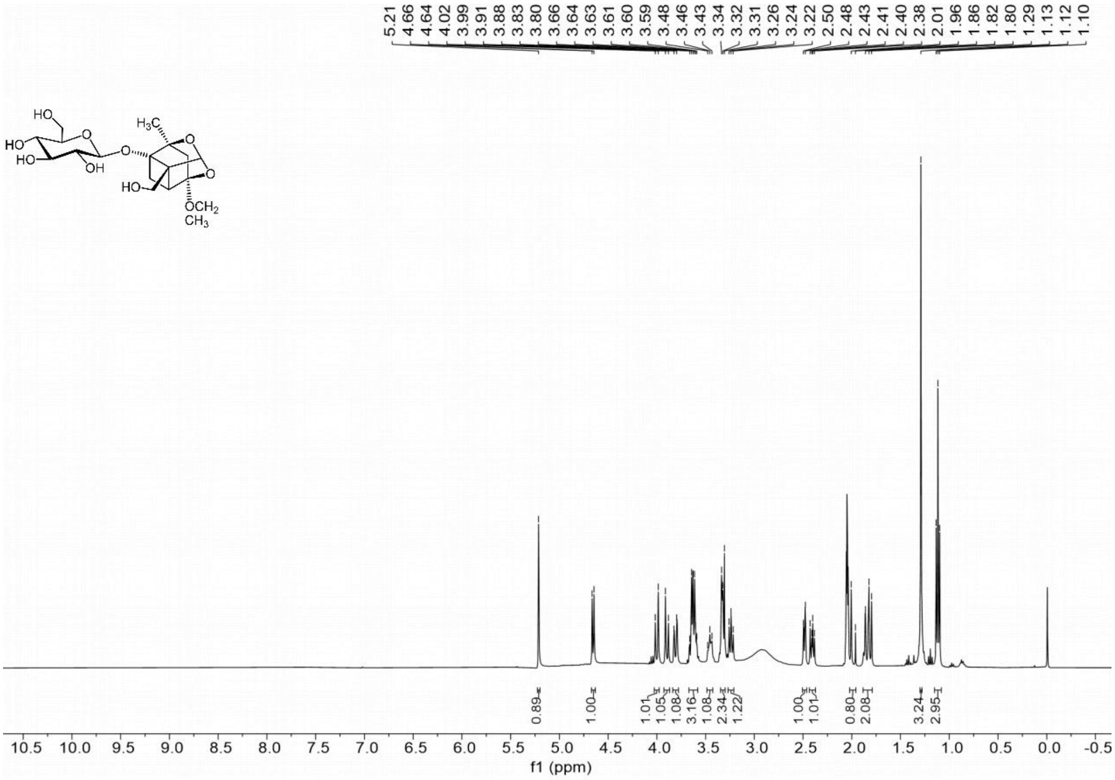

Figure S10. The ^{1}H NMR of compound **9**.

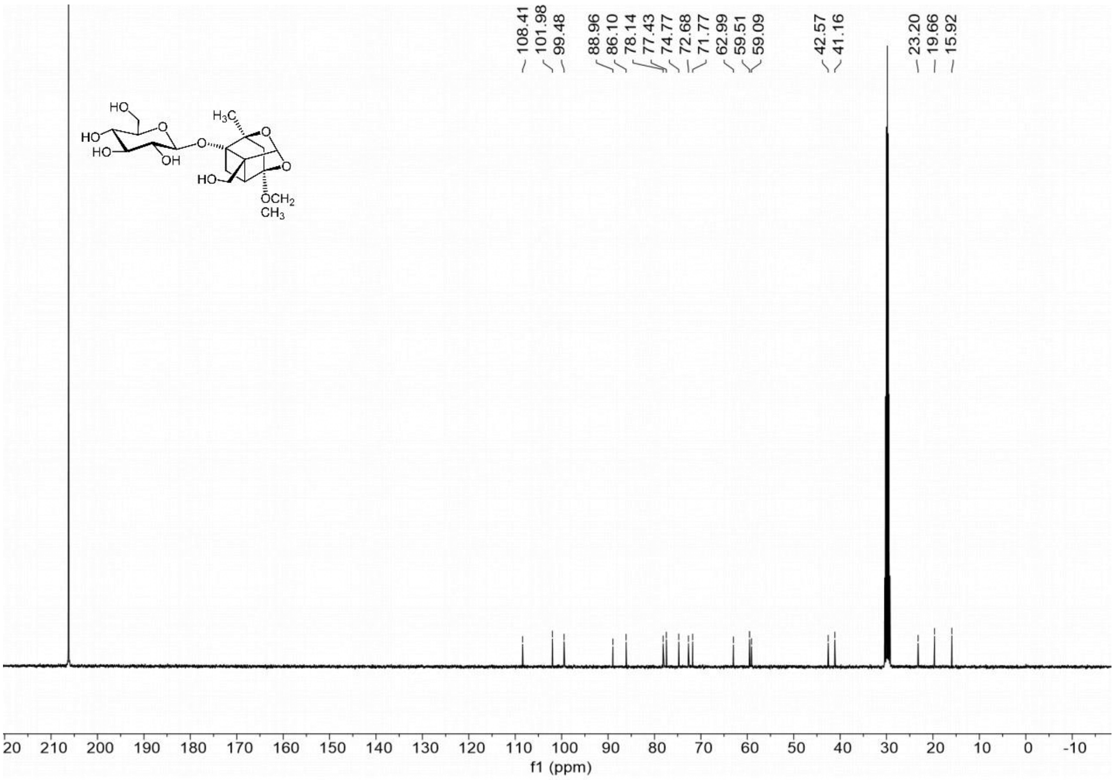

Figure S11. The ^{13}C NMR of compound **9**.

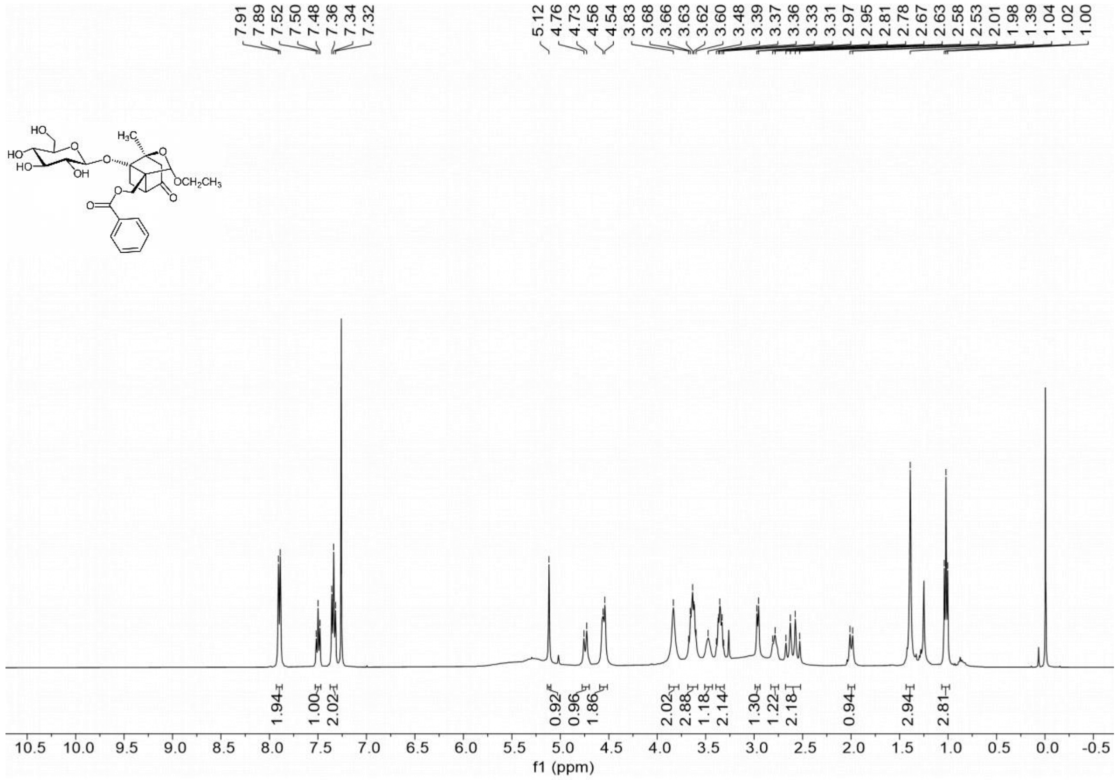

Figure S12. The ^{1}H NMR of compound **10**.

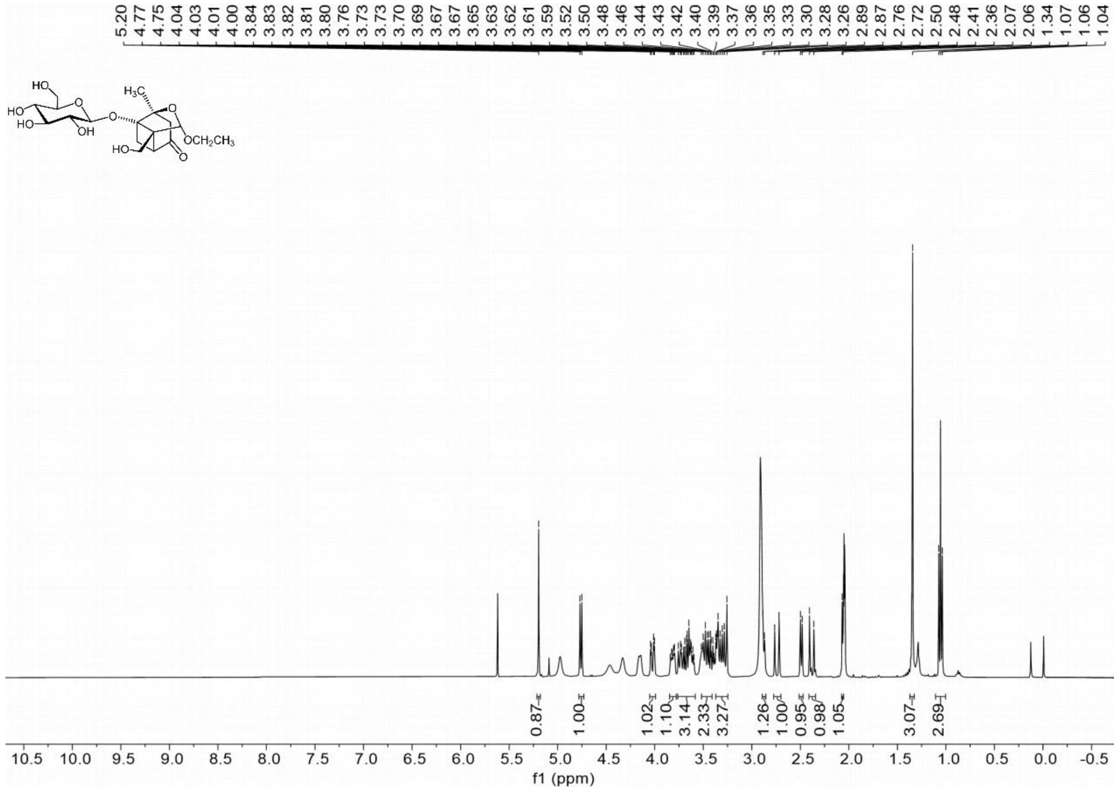

Figure S13. The ^{1}H NMR of compound **11**.

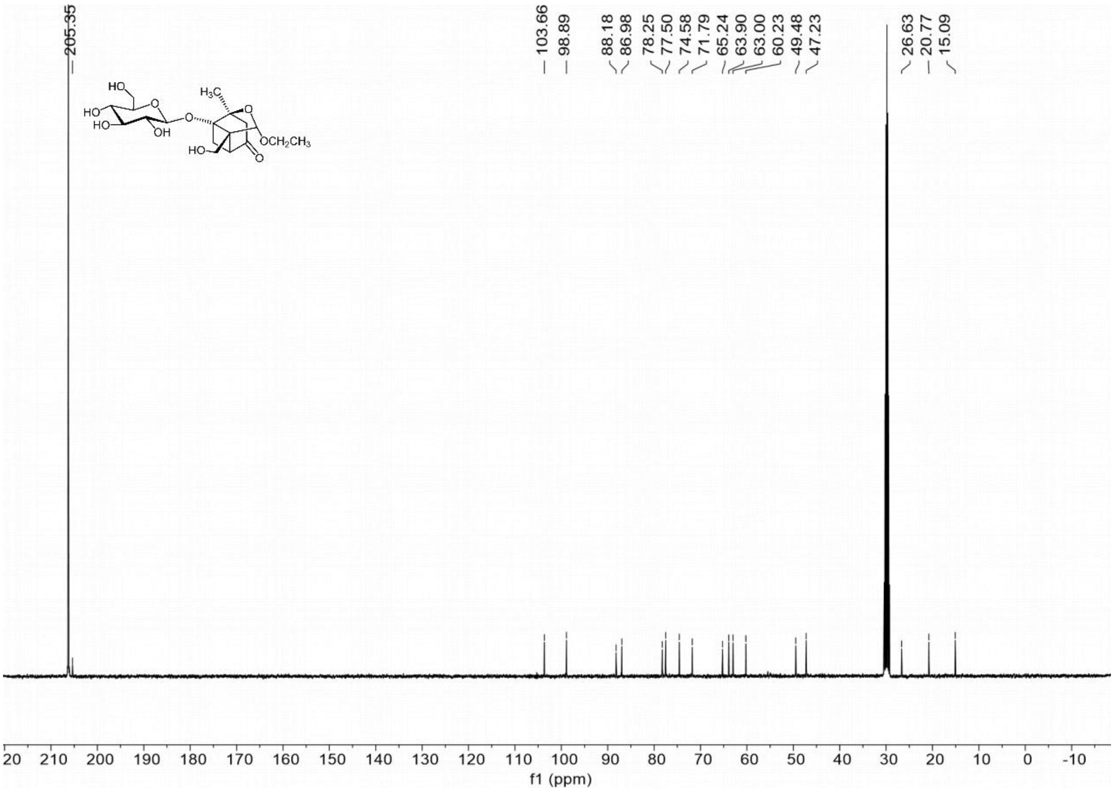

Figure S14. The ^{13}C NMR of compound **11**.

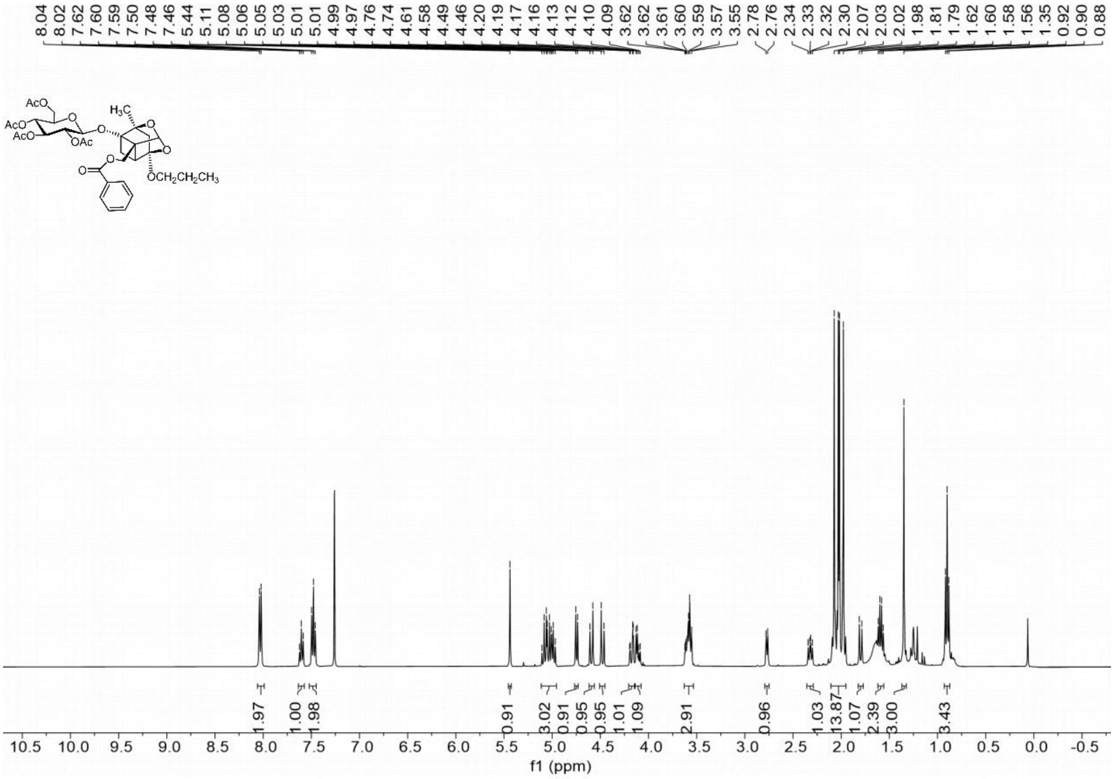

Figure S15. The ^{1}H NMR of compound **12**.

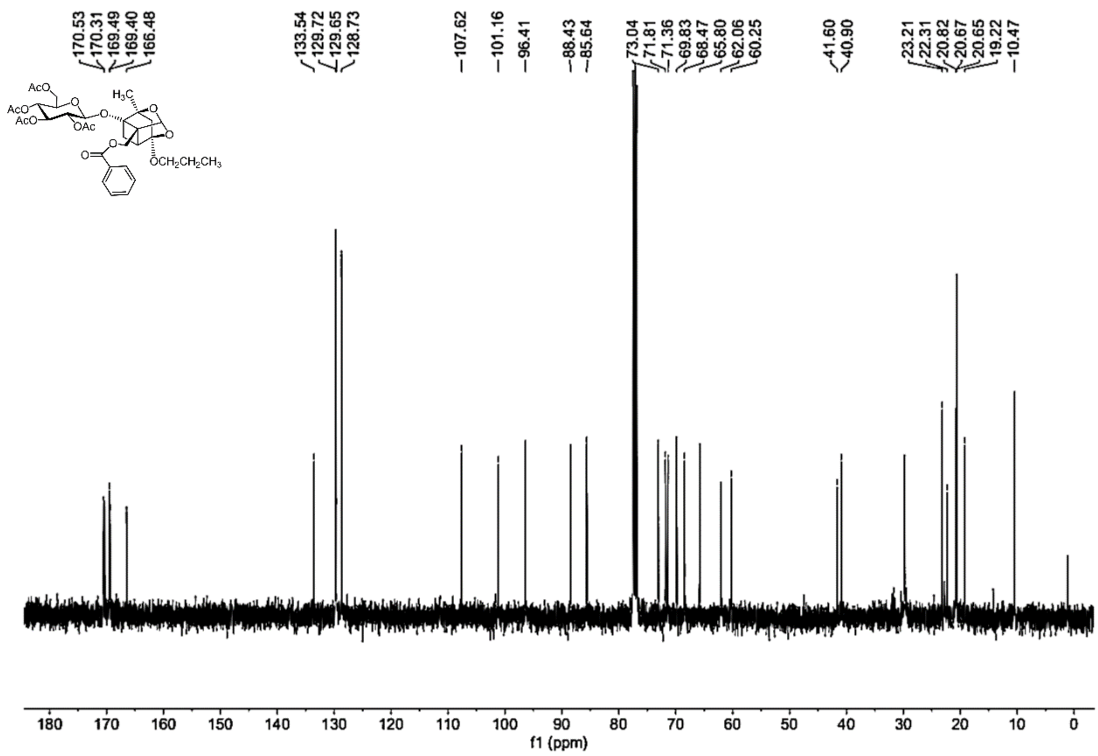

Figure S16. The ^{13}C NMR of compound **12**.

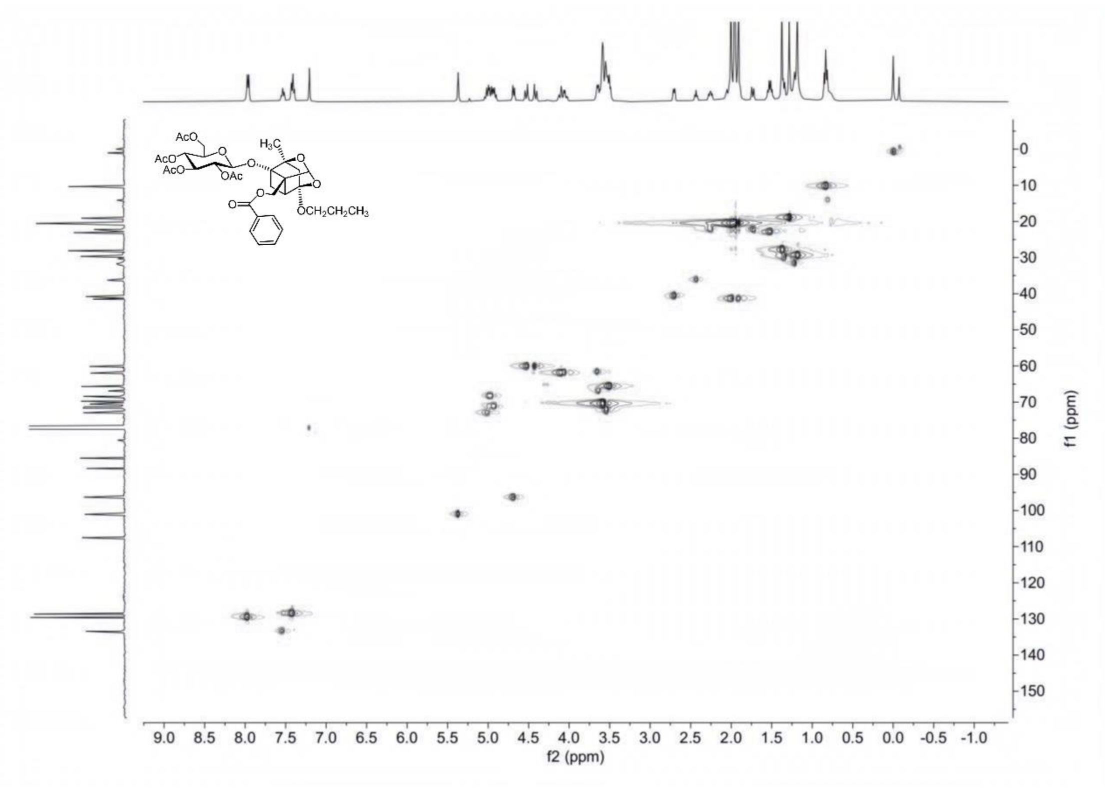

Figure S17. The HSQC-NMR of compound **12**.

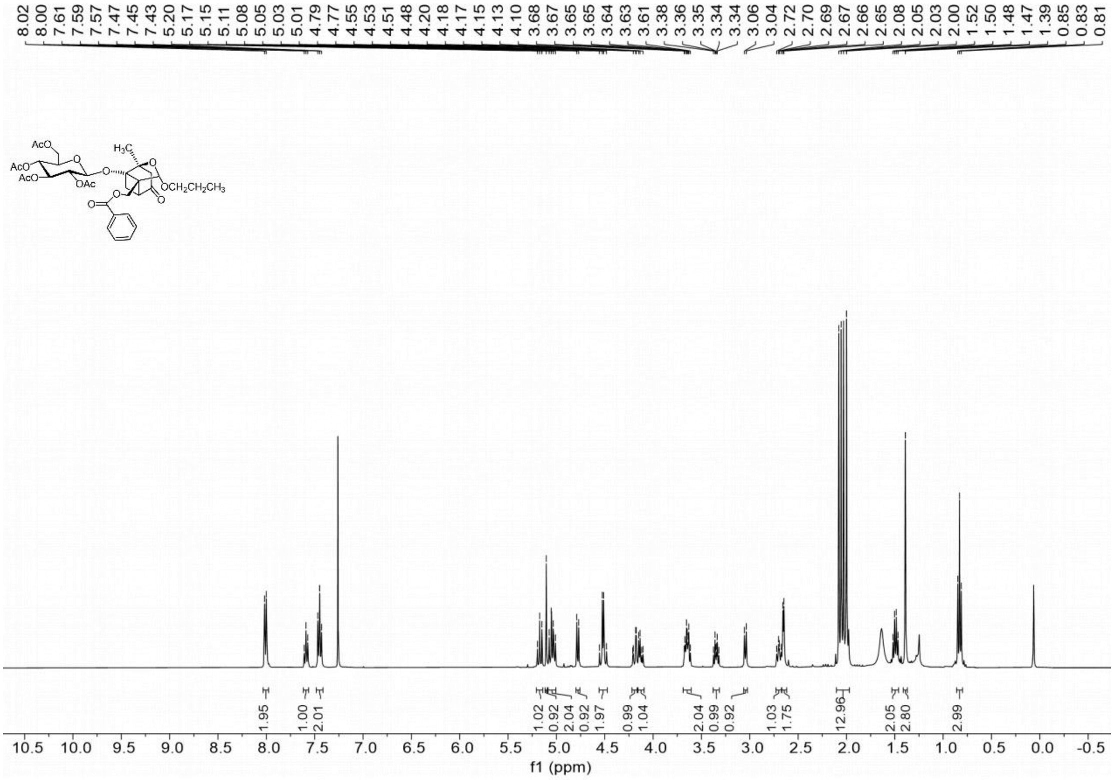

Figure S18. The ^{1}H NMR of compound **13**.

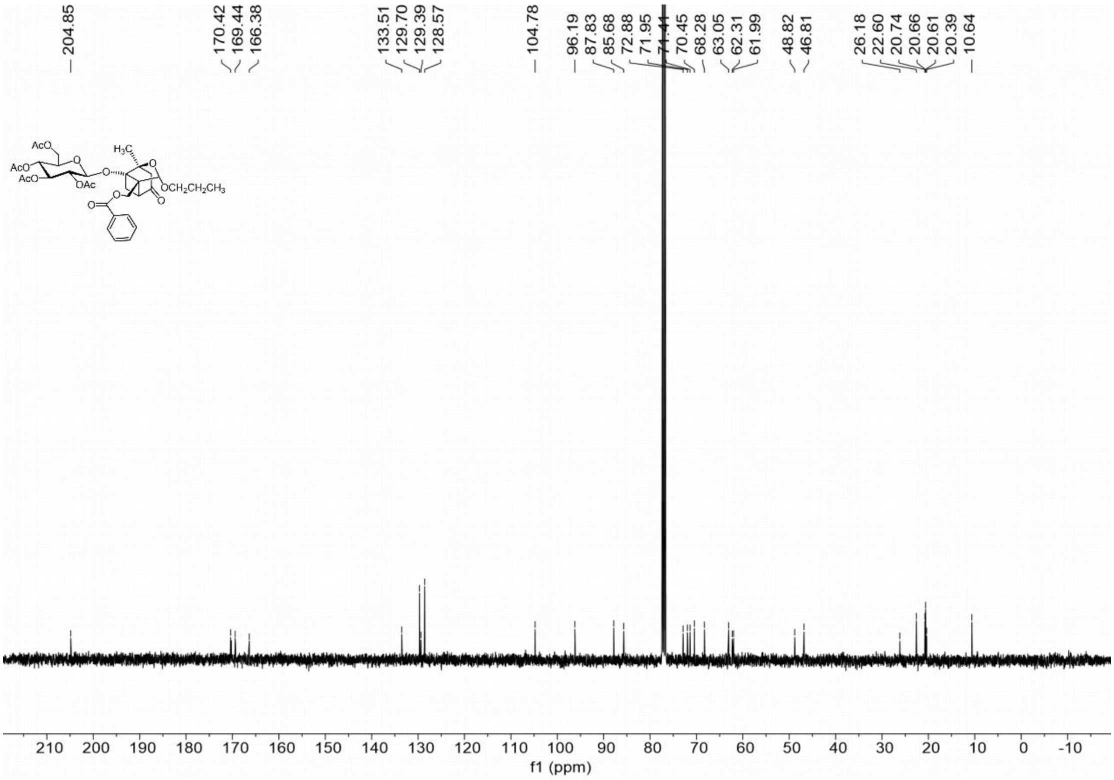

Figure S19. The ^{13}C NMR of compound **13**.

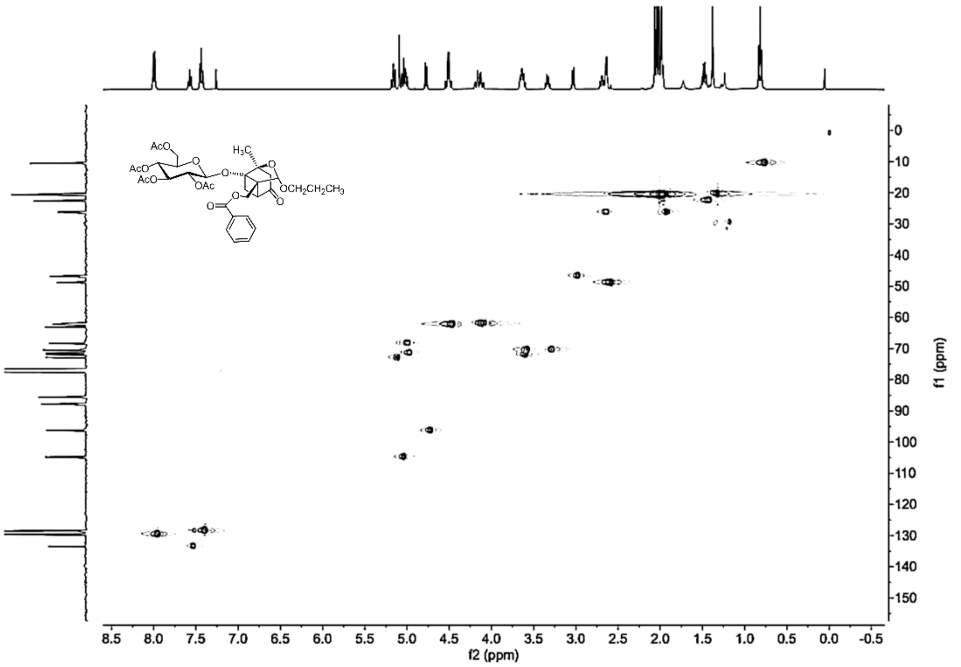

Figure S20. The HSQC-NMR of compound **13**.

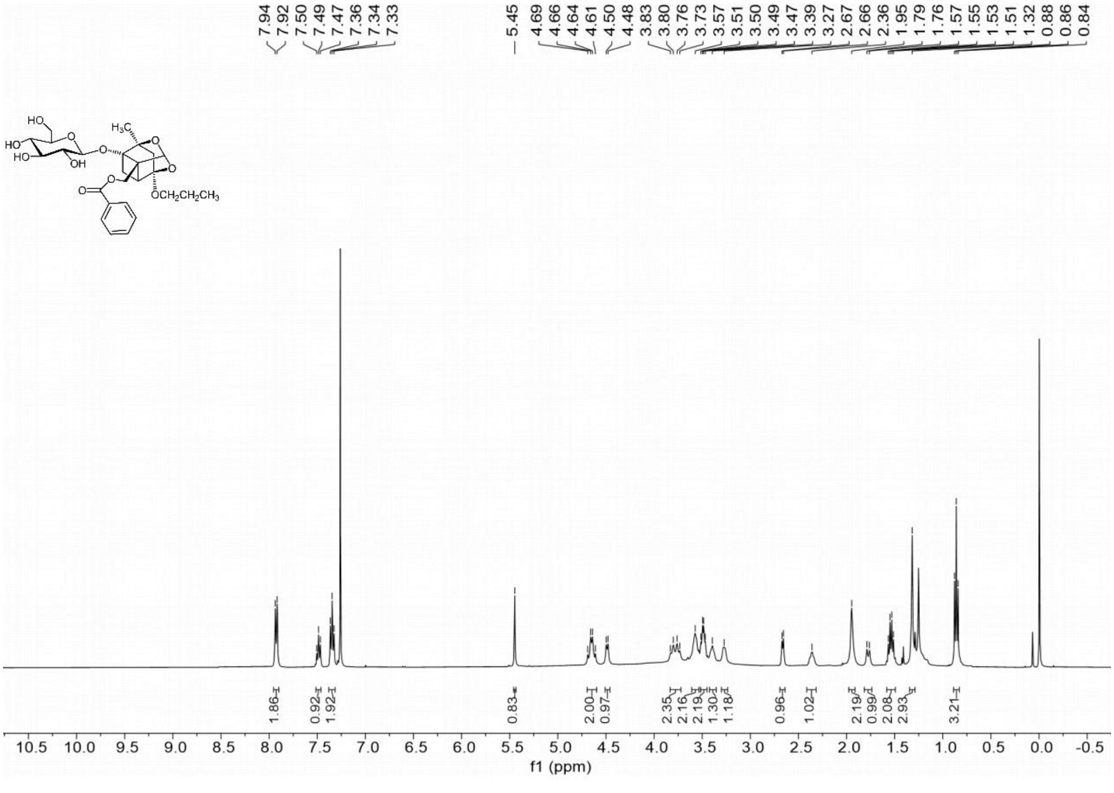

Figure S21. The ^{1}H NMR of compound **14**.

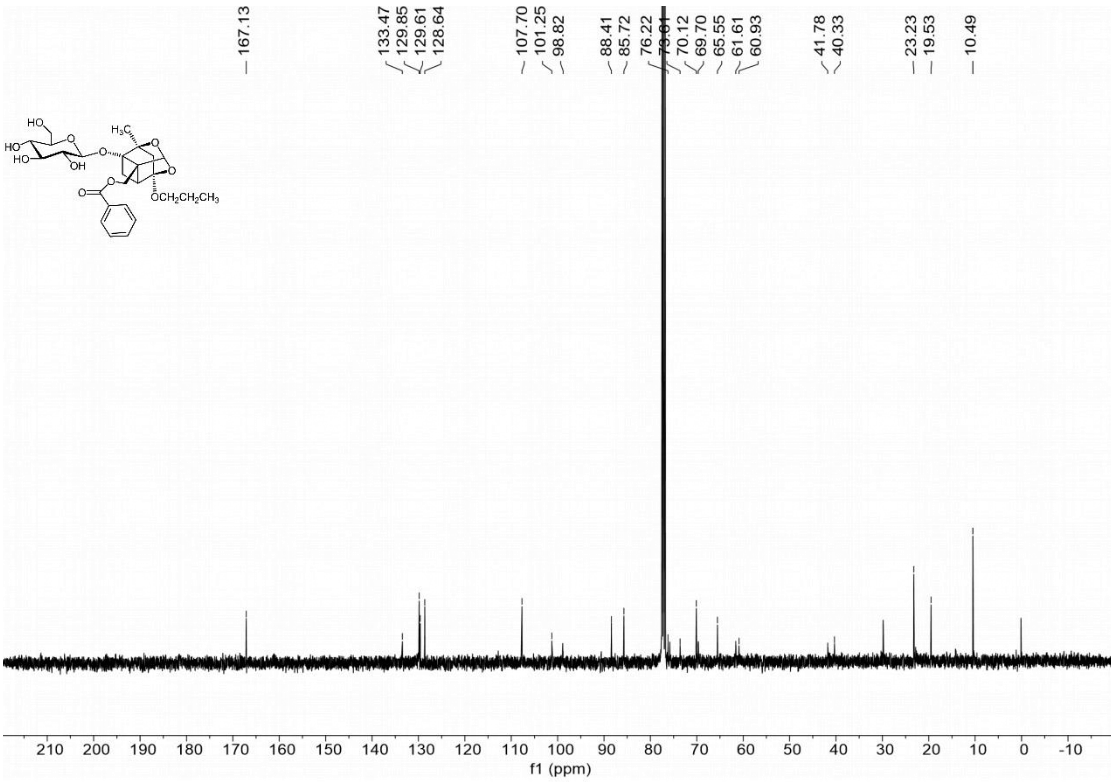

Figure S22. The ^{13}C NMR of compound **14**.

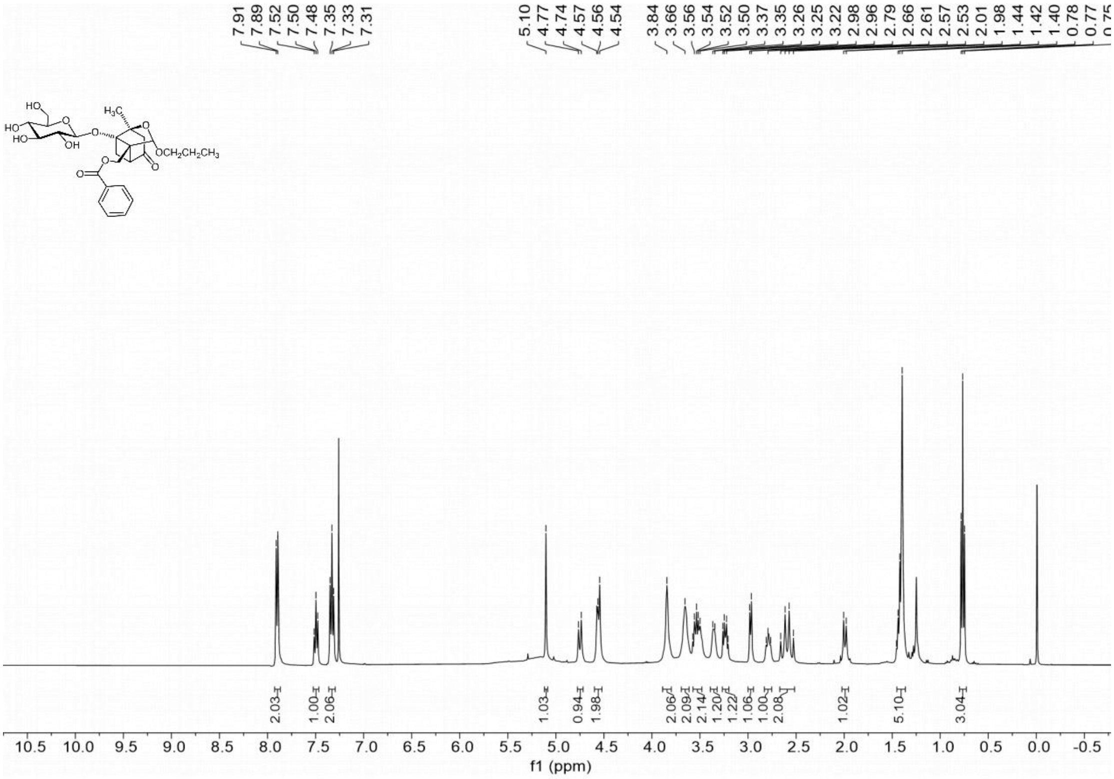

Figure S23. The ^{1}H NMR of compound **15**

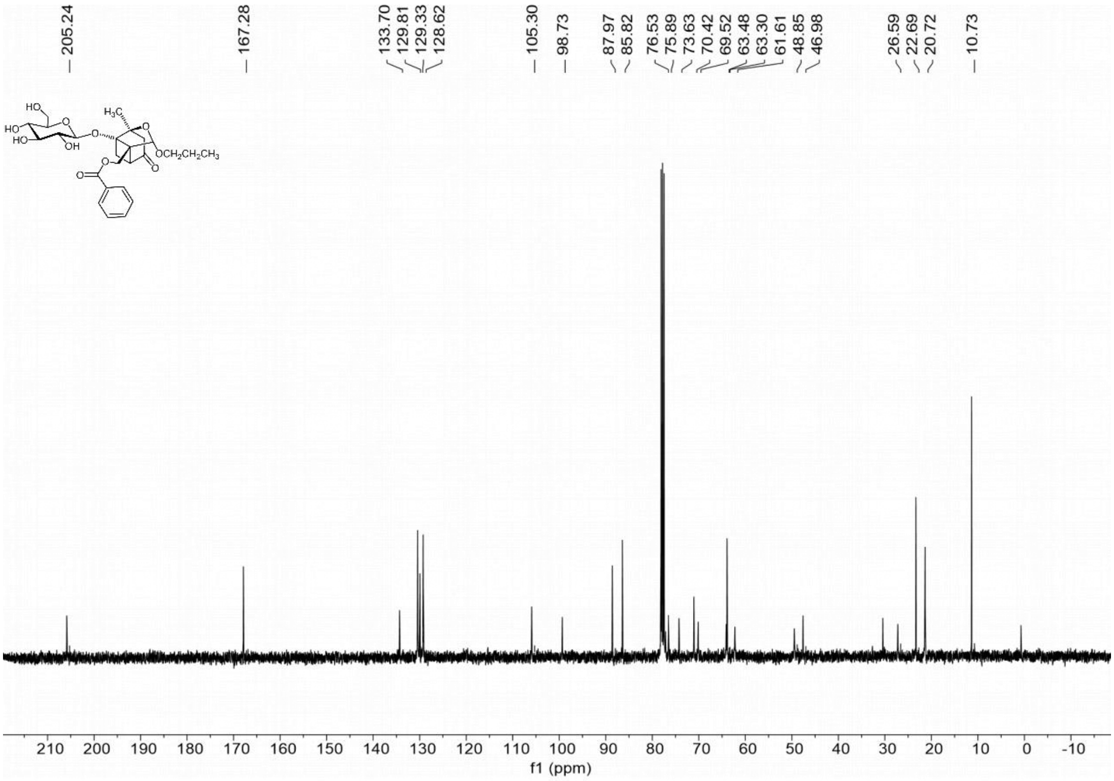

Figure S24. The ^{13}C NMR of compound **15**.

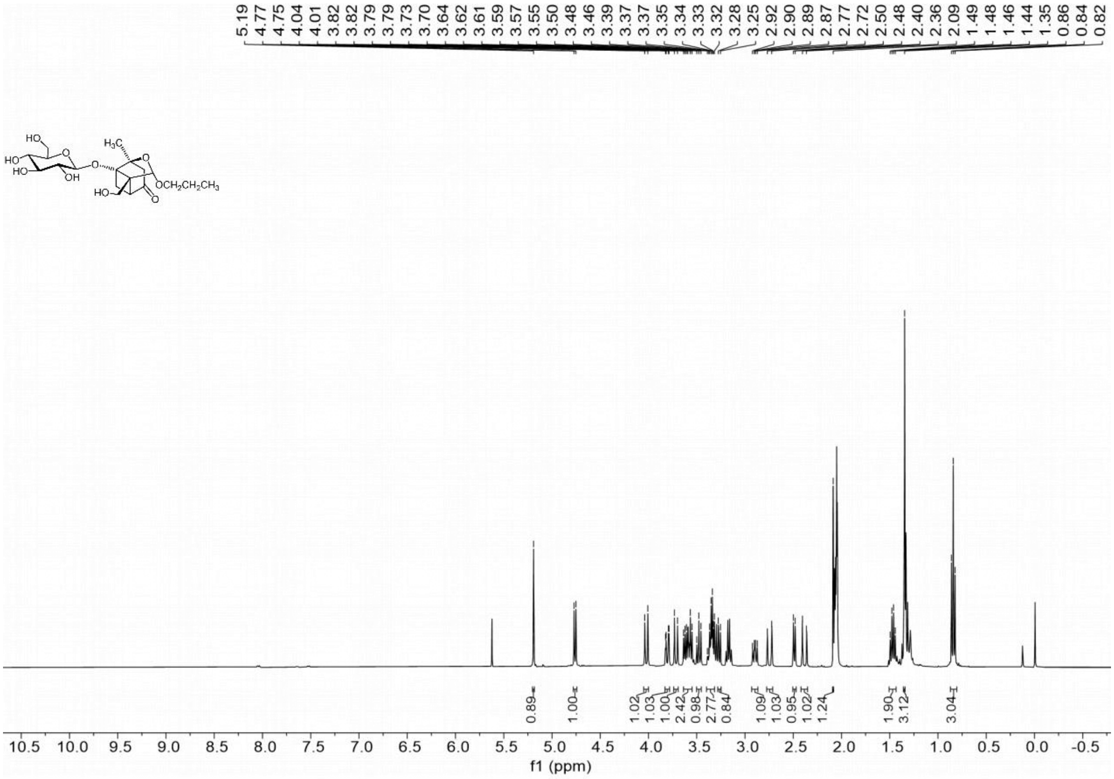

Figure S25. The ^{1}H NMR of compound **16**.

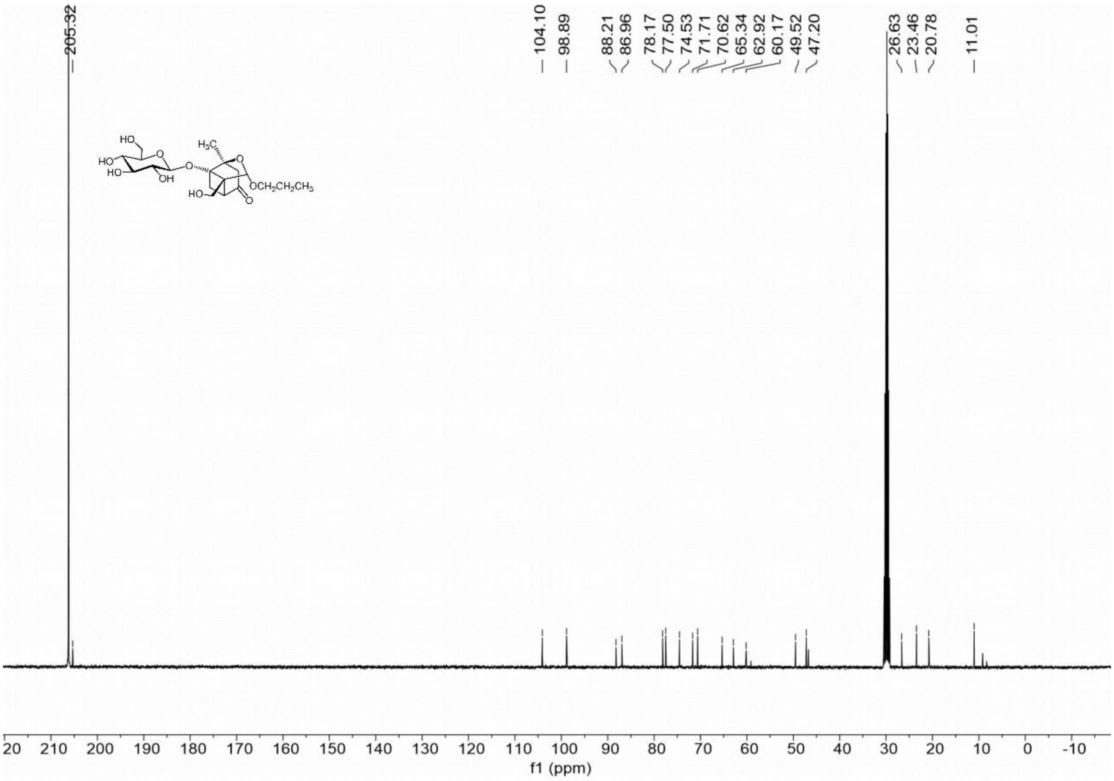

Figure S26. The ^{13}C NMR of compound **16**.

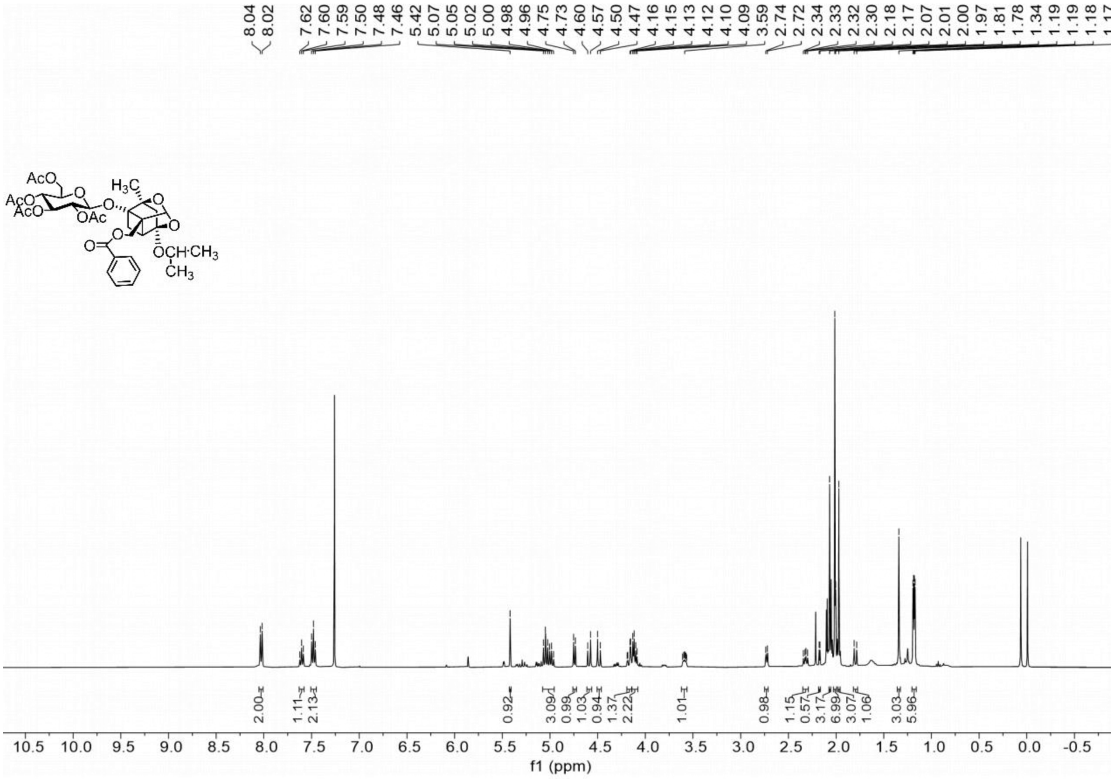

Figure S27. The ^{1}H NMR of compound **17**.

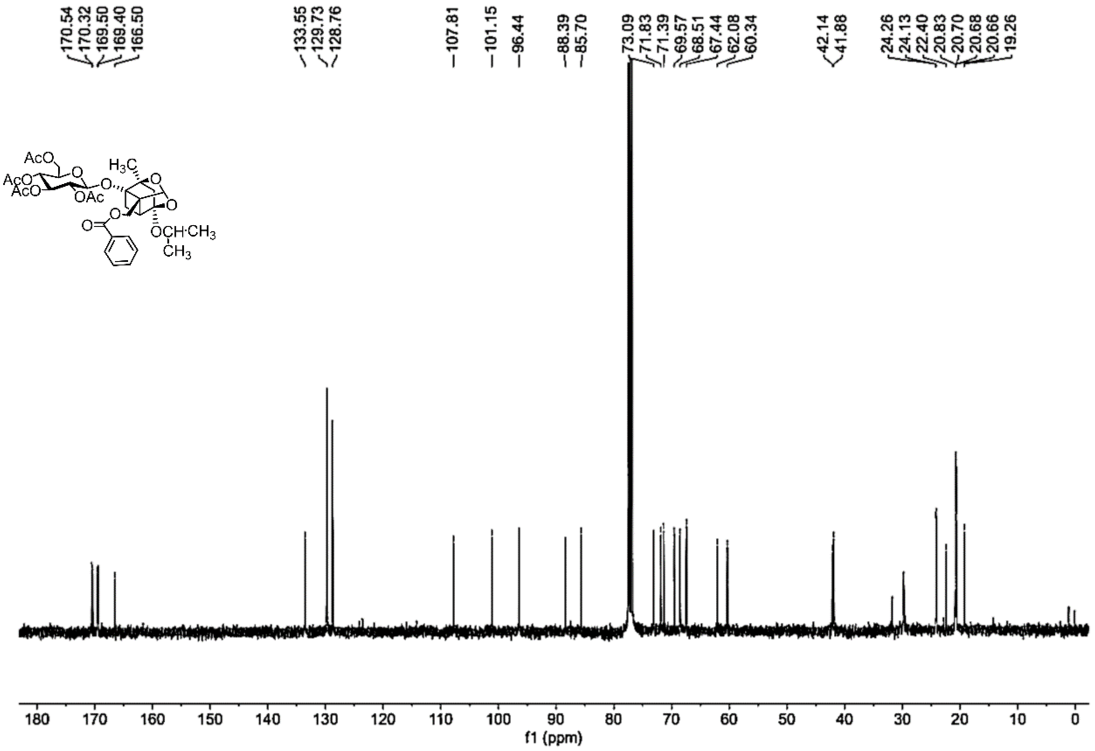

Figure S28. The ^{13}C NMR of compound **17**.

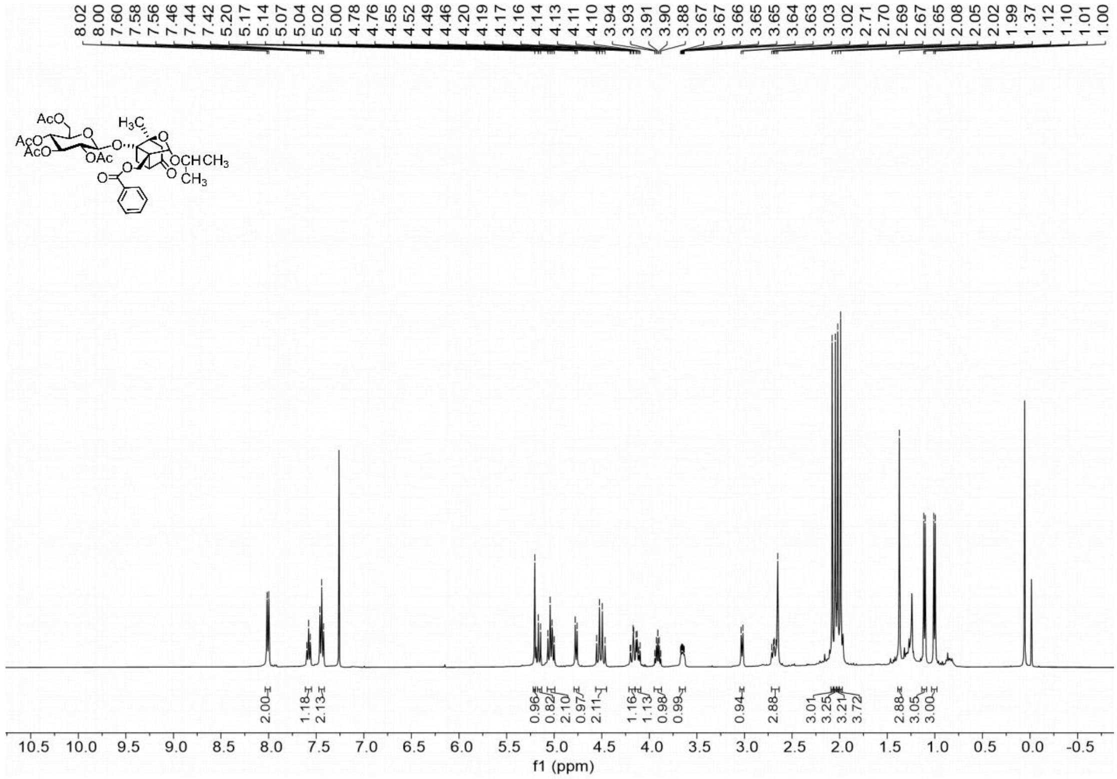

Figure S29. The ^{1}H NMR of compound **18**.

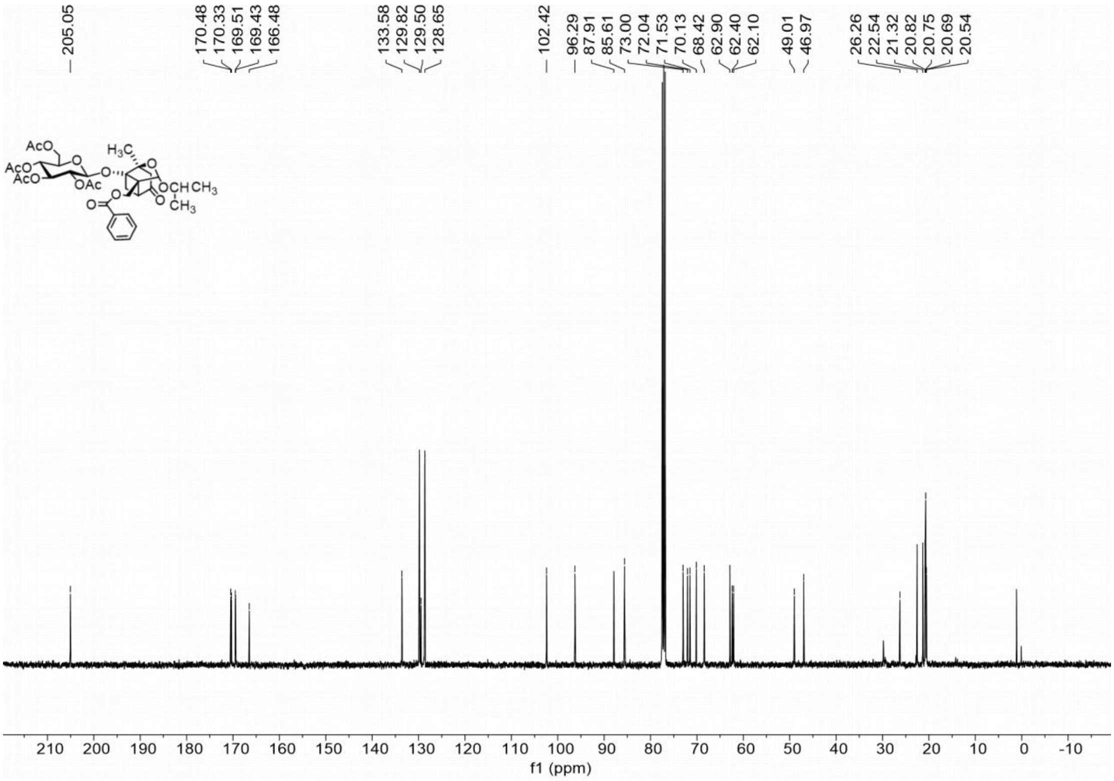

Figure S30. The ^{13}C NMR of compound **18**.

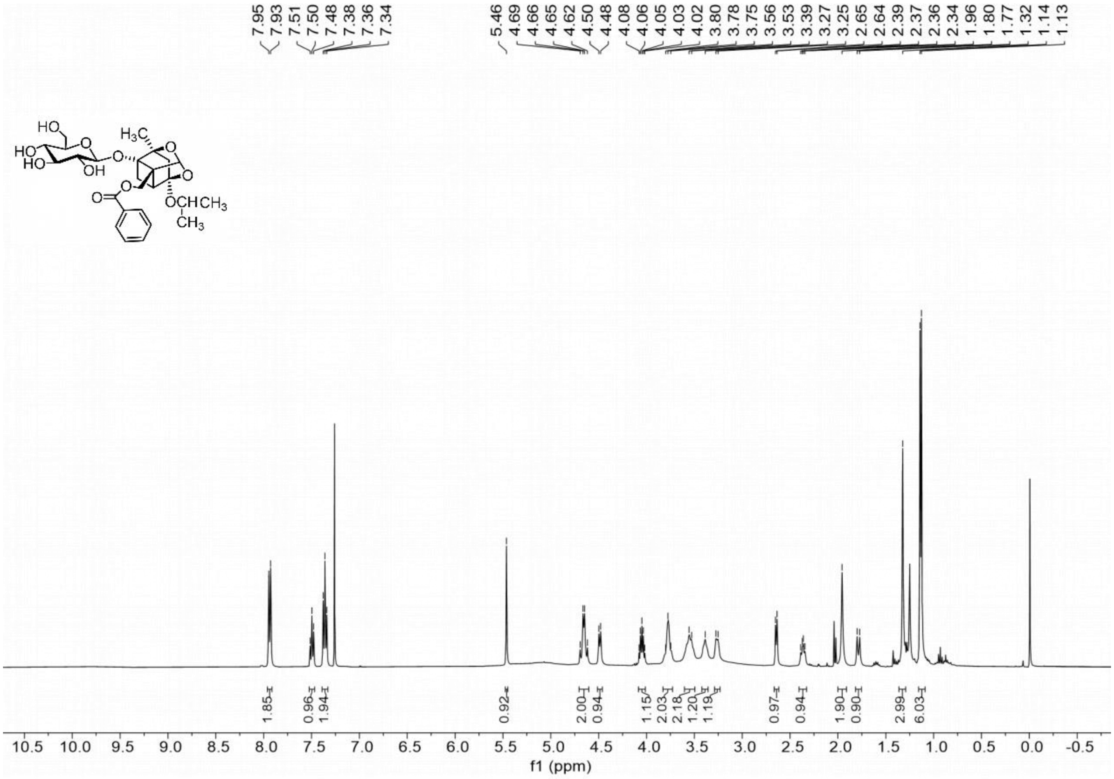

Figure S31. The ^{1}H NMR of compound **19**.

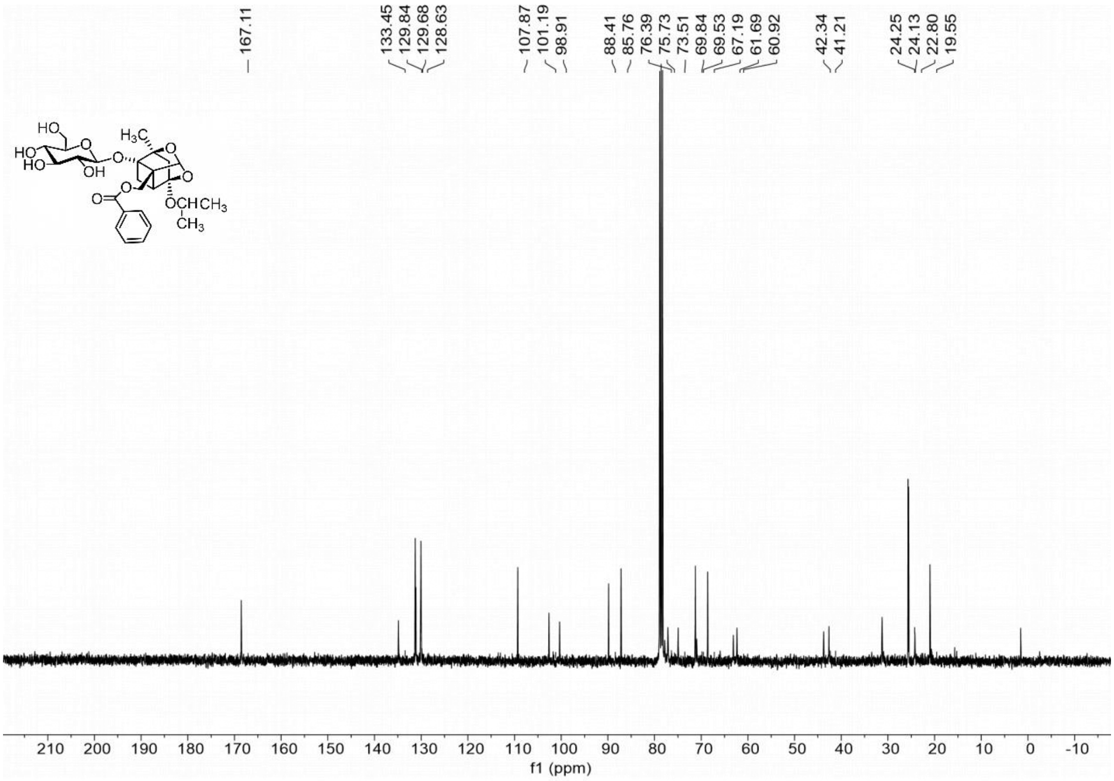

Figure S32. The ^{13}C NMR of compound **19**.

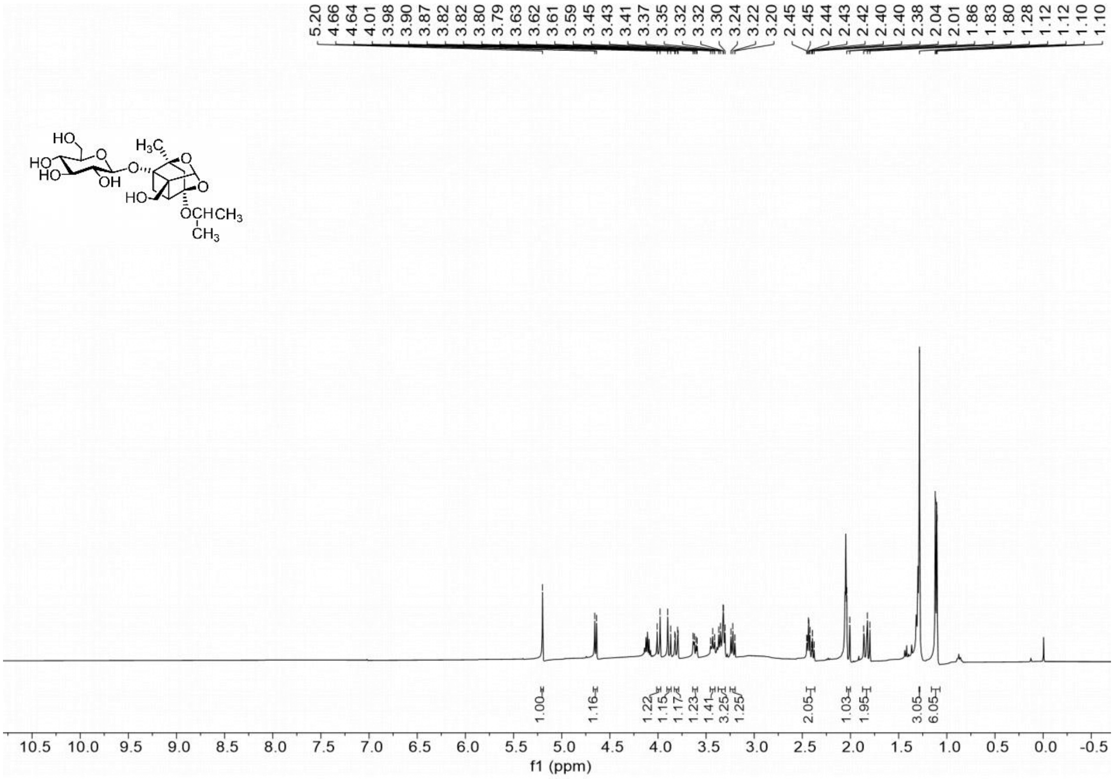

Figure S33. The ^{1}H NMR of compound **20**.

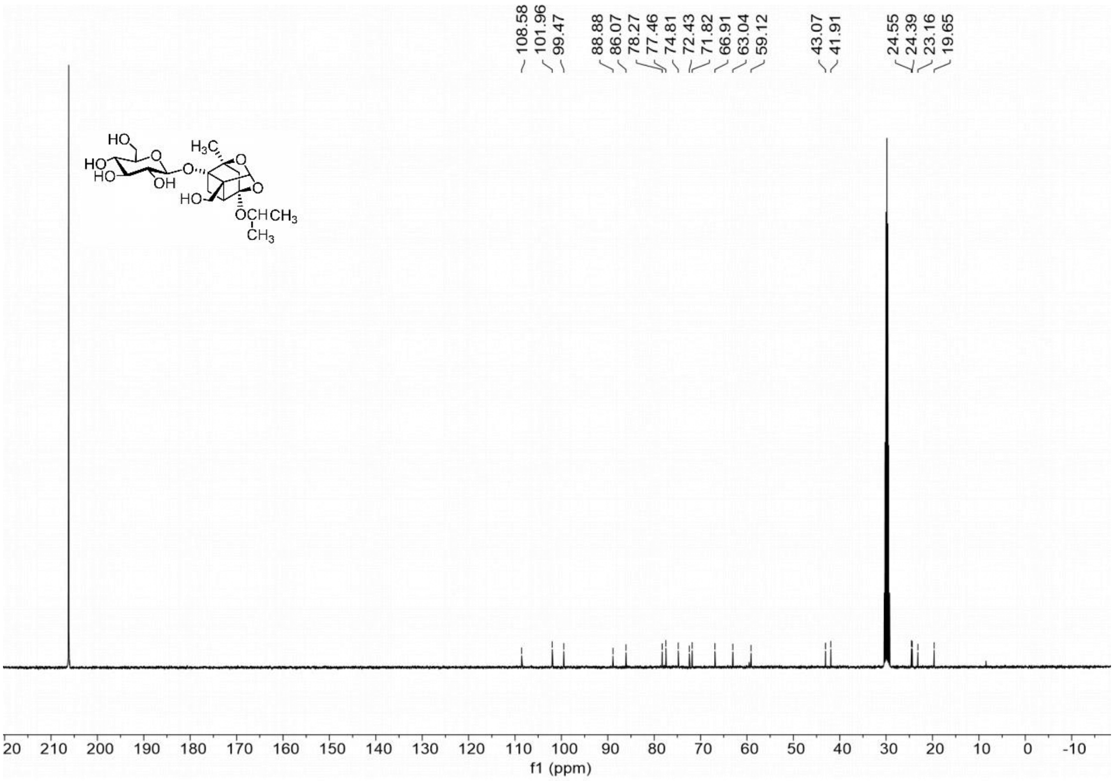

Figure S34. The ^{13}C NMR of compound **20**.

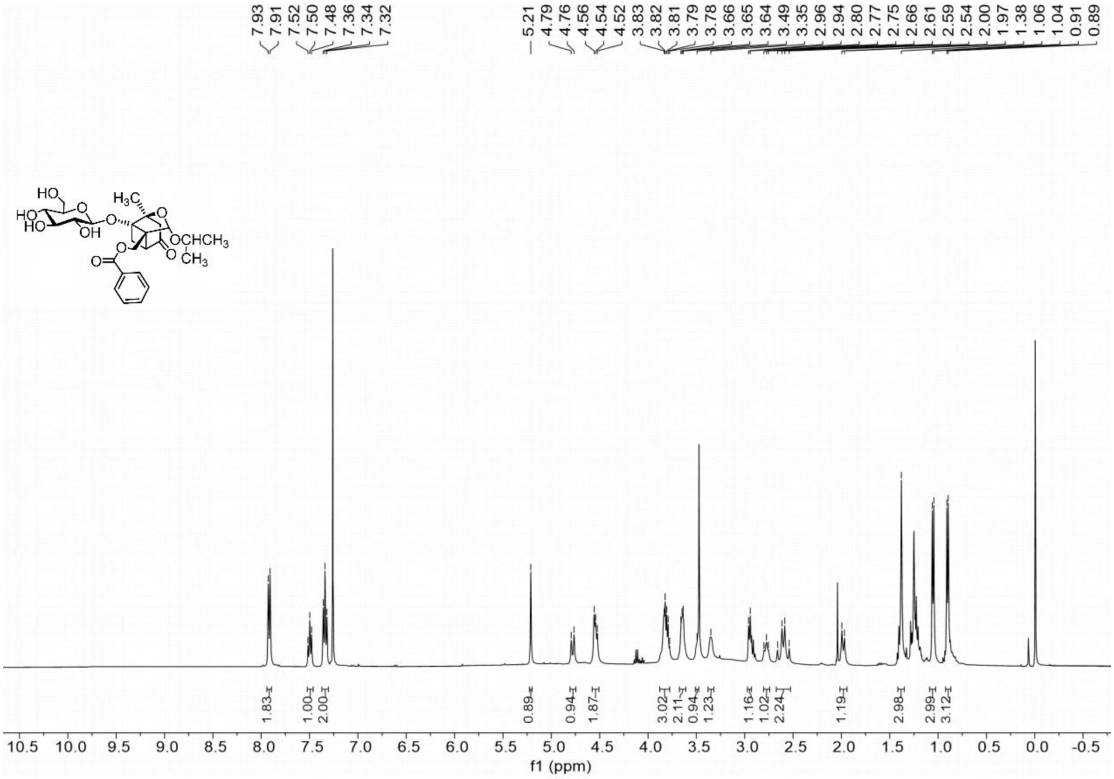

Figure S35. The ^{1}H NMR of compound **21**.

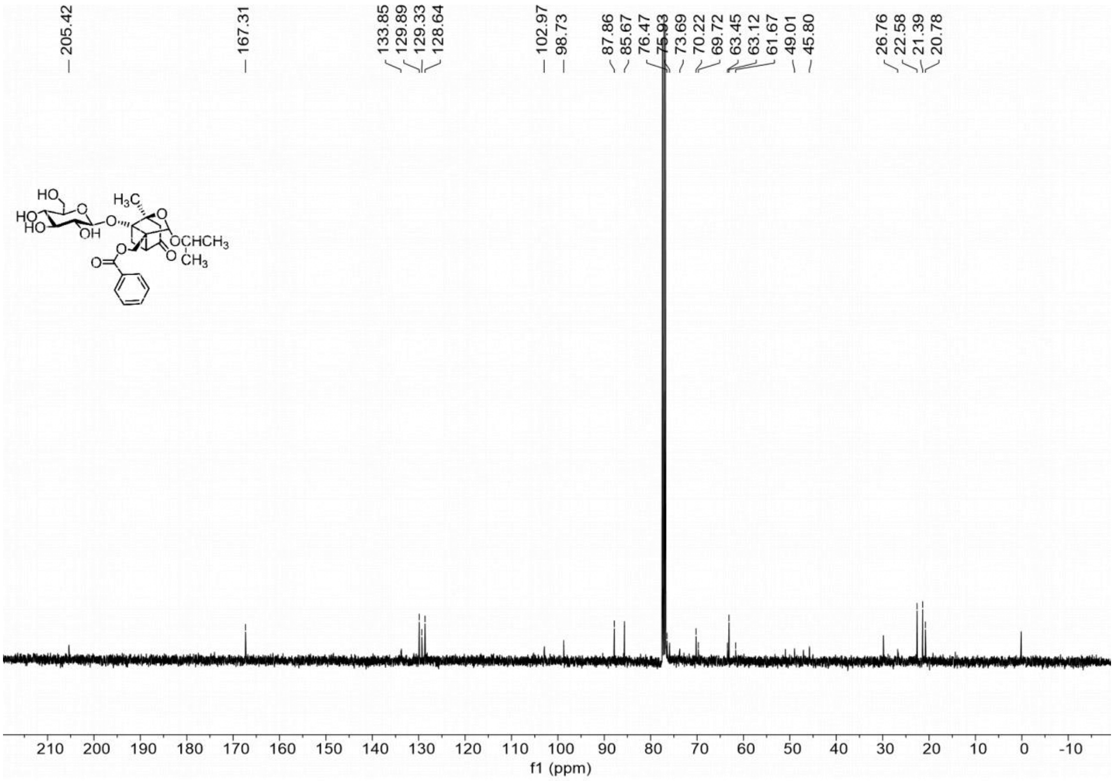

Figure S36. The ^{13}C NMR of compound **21**.

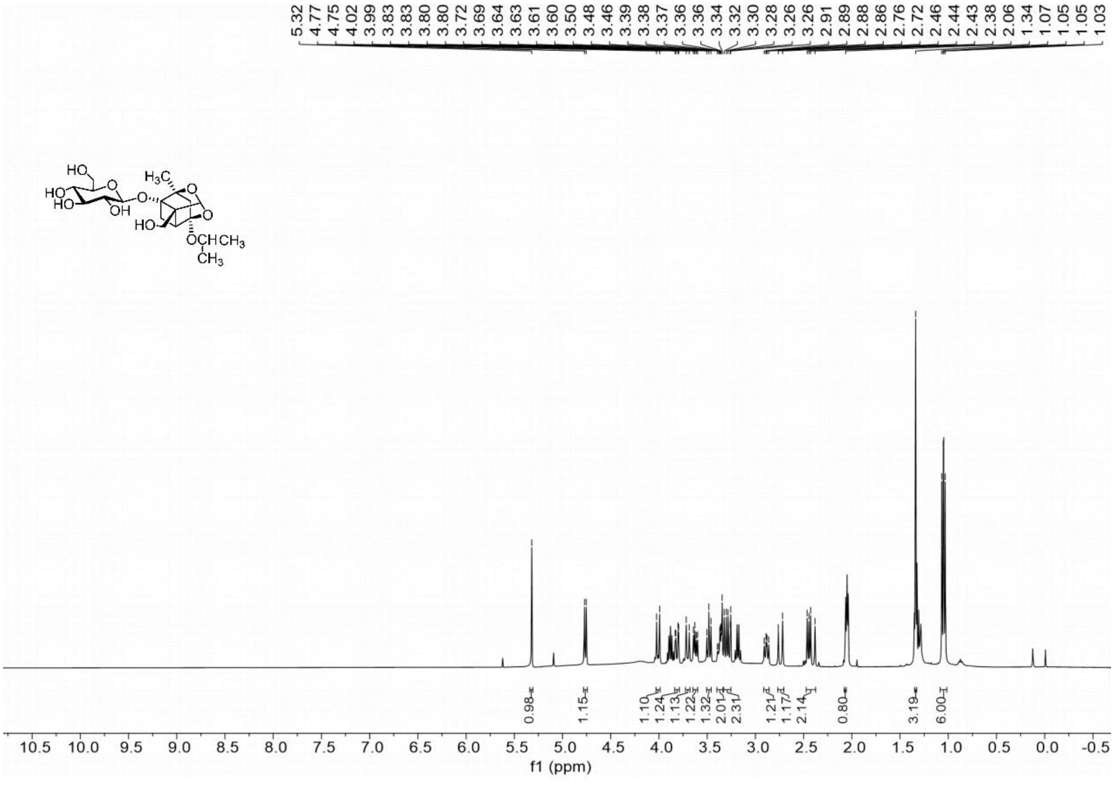

Figure S37. The ^{1}H NMR of compound **22**.

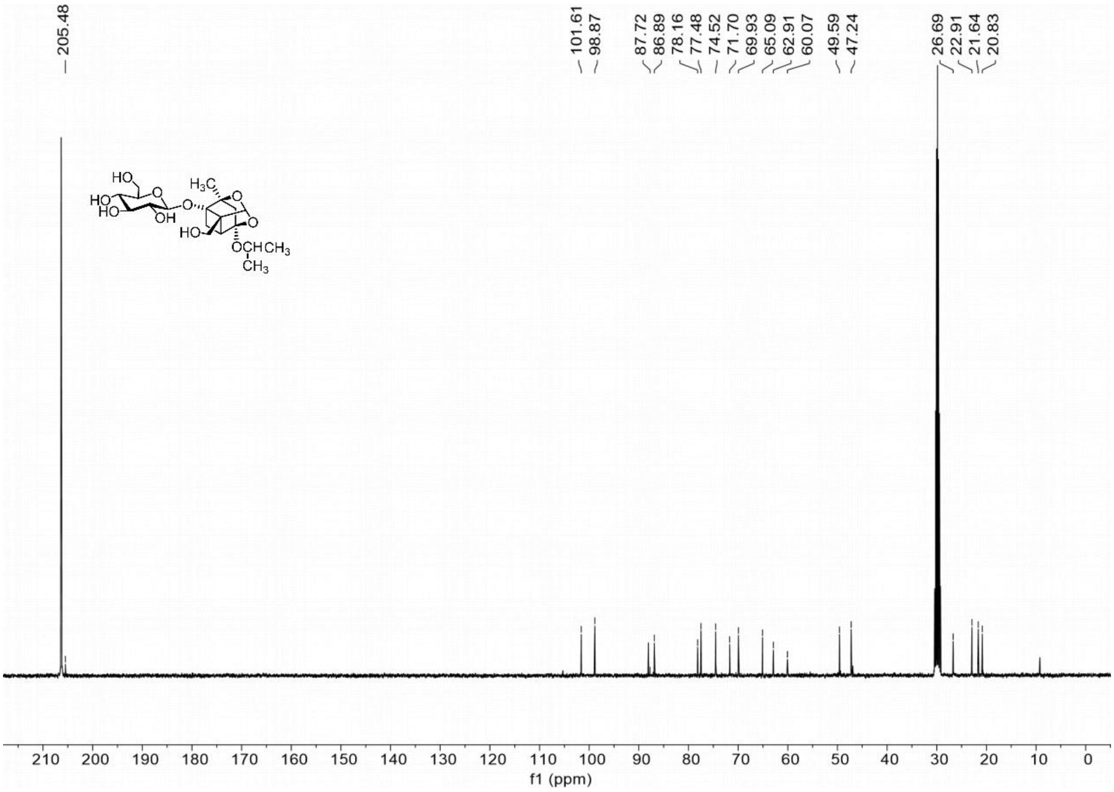

Figure S38. The ^{13}C NMR of compound **22**.

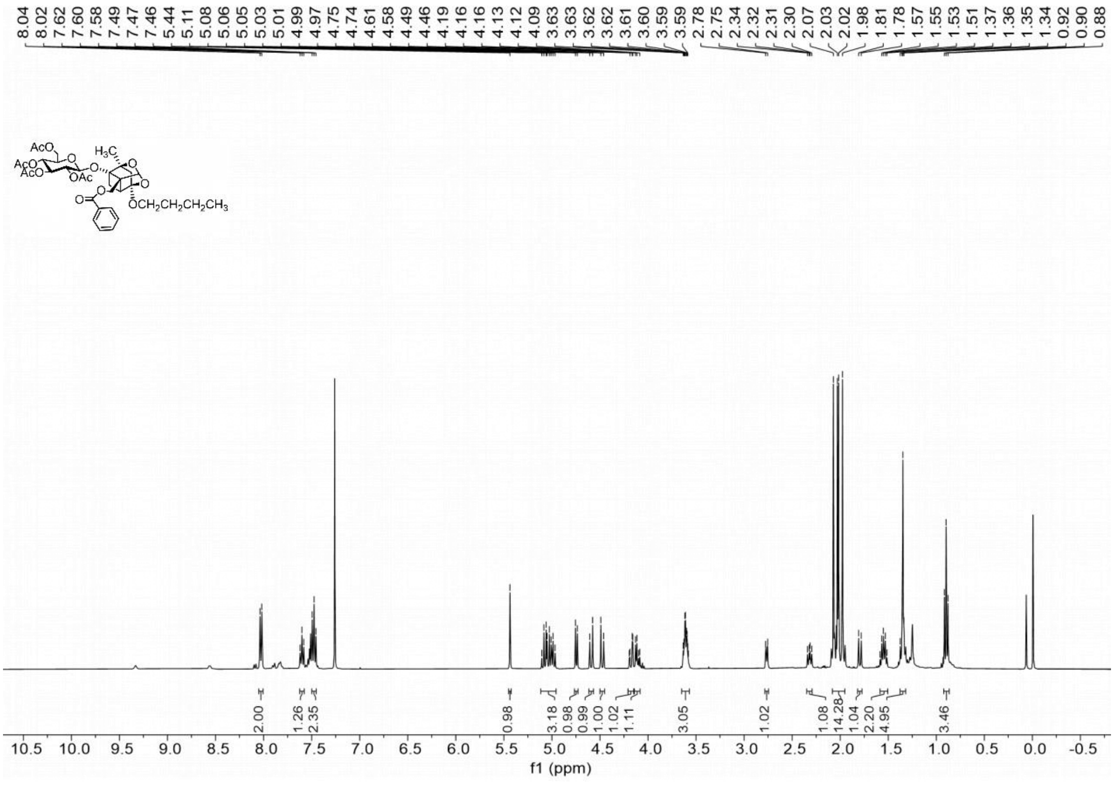

Figure S39. The ^{1}H NMR of compound **23**.

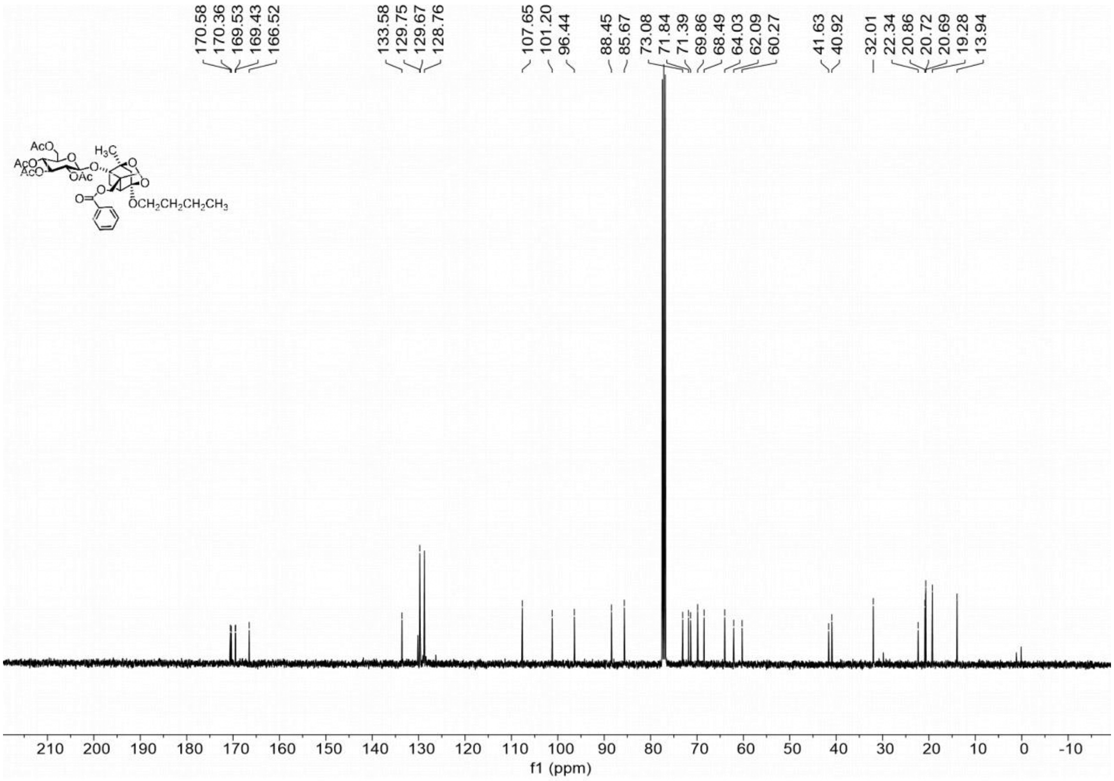

Figure S40. The ^{13}C NMR of compound **23**.

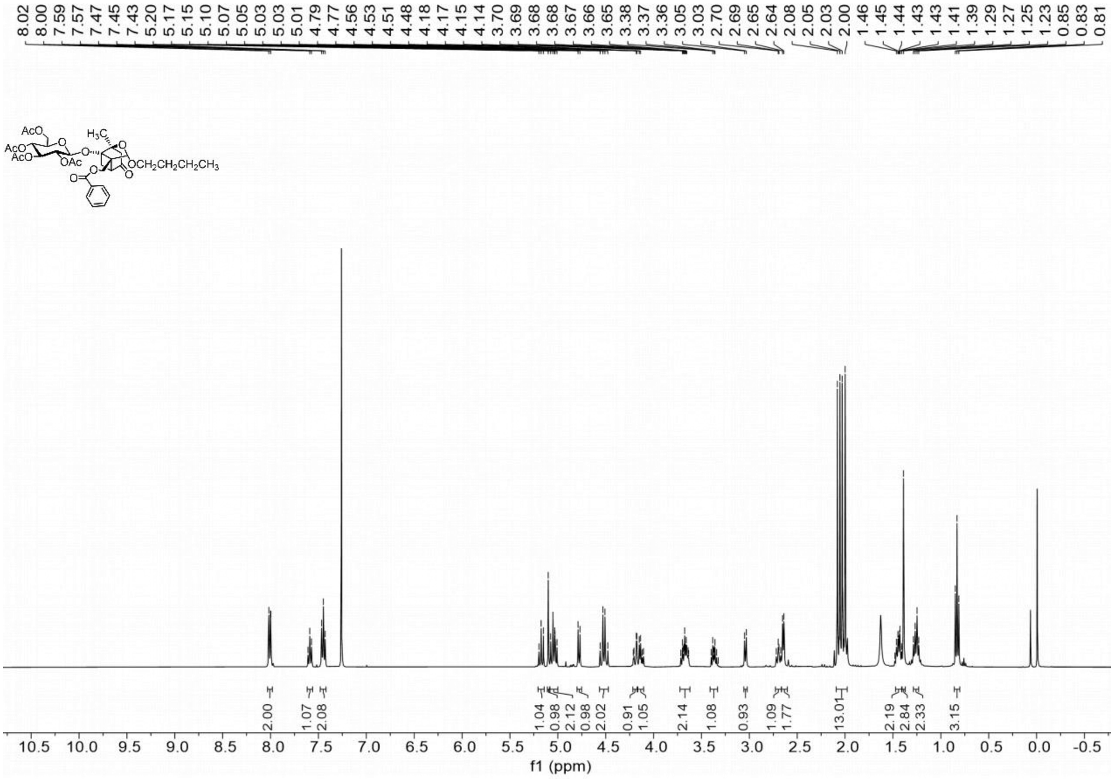

Figure S41. The ^{1}H NMR of compound **24**.

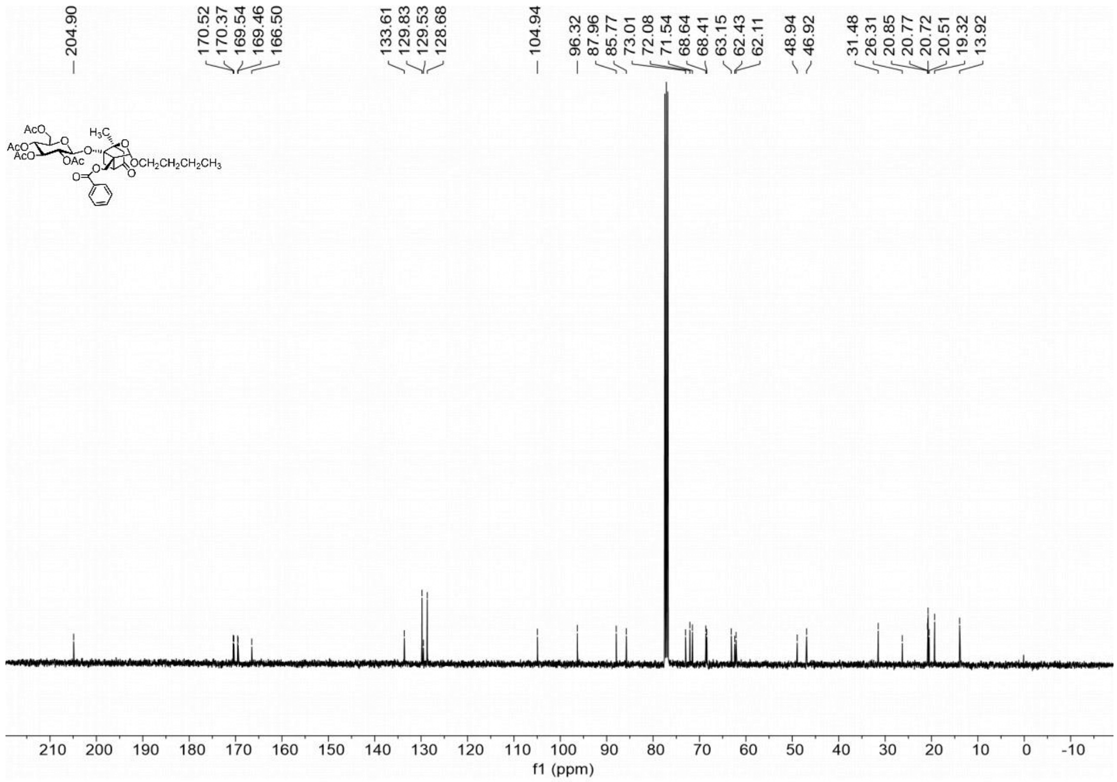

Figure S42. The ^{13}C NMR of compound **24**.

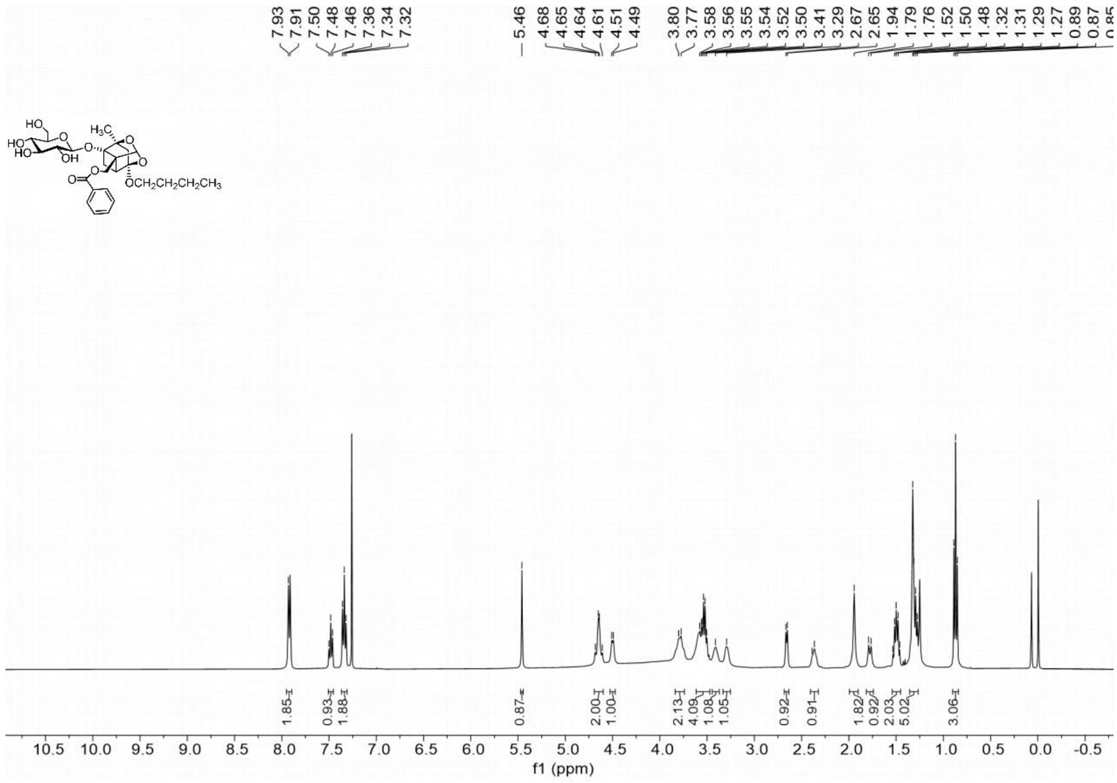

Figure S43. The ^{1}H NMR of compound **25**.

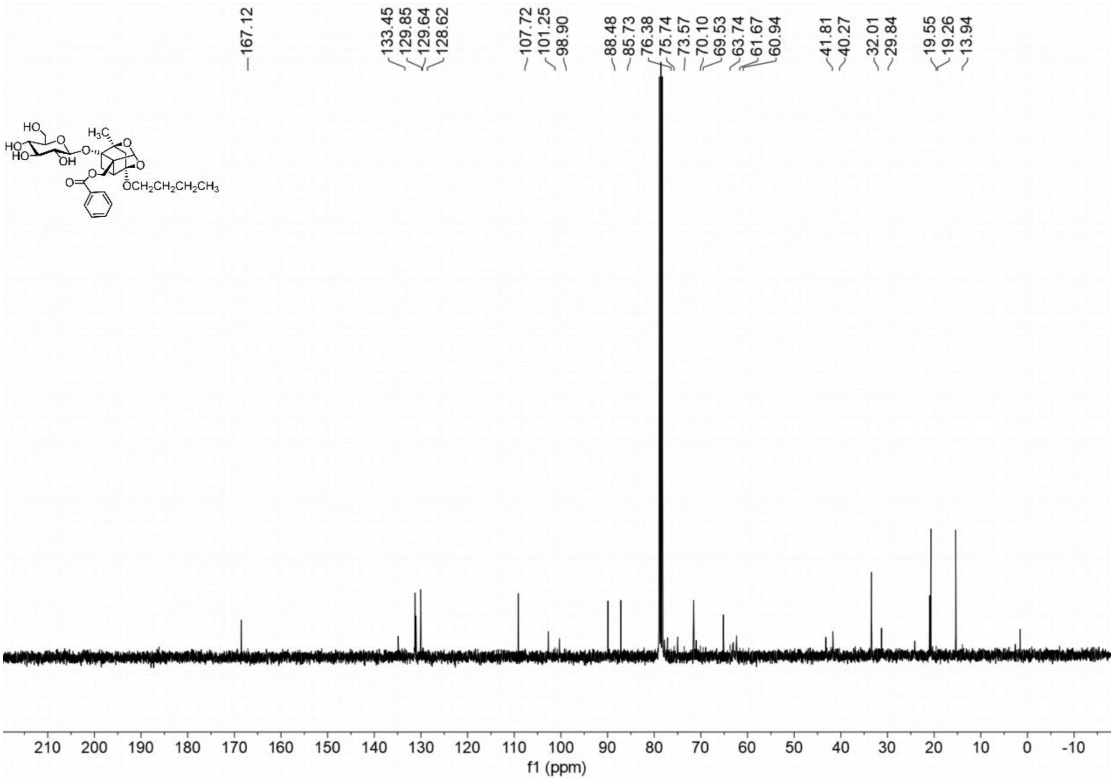

Figure S44. The ^{13}C NMR of compound **25**.

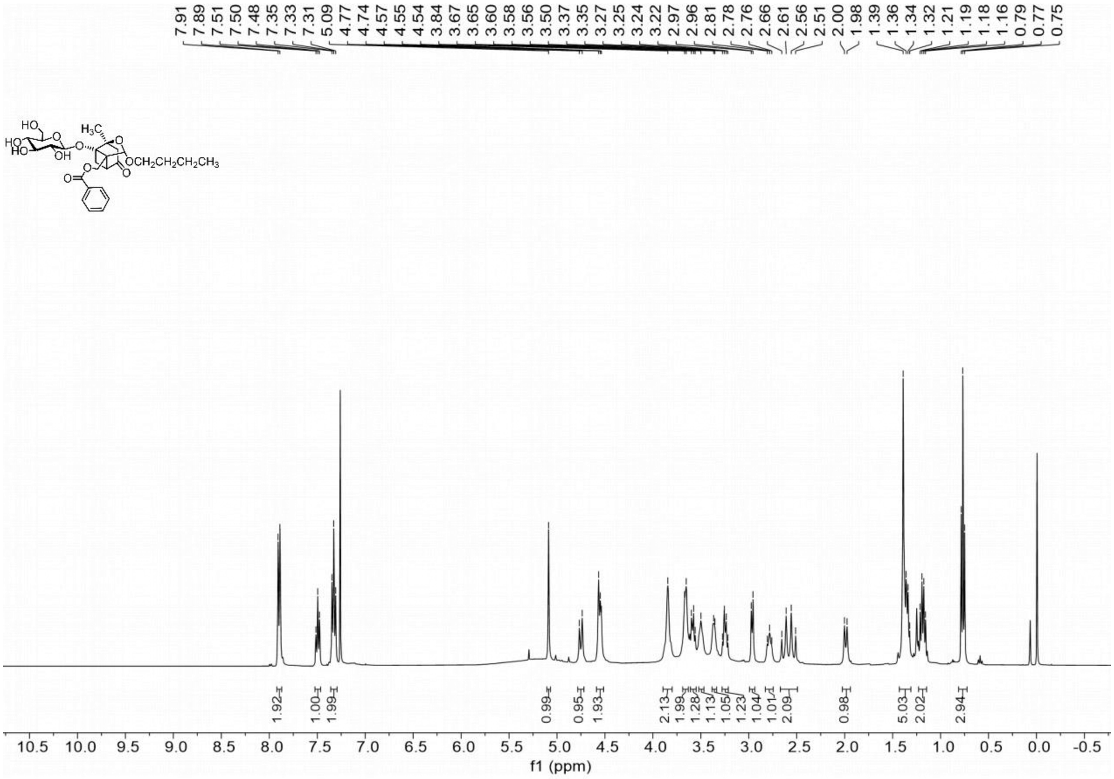

Figure S45. The ^{1}H NMR of compound **26**.

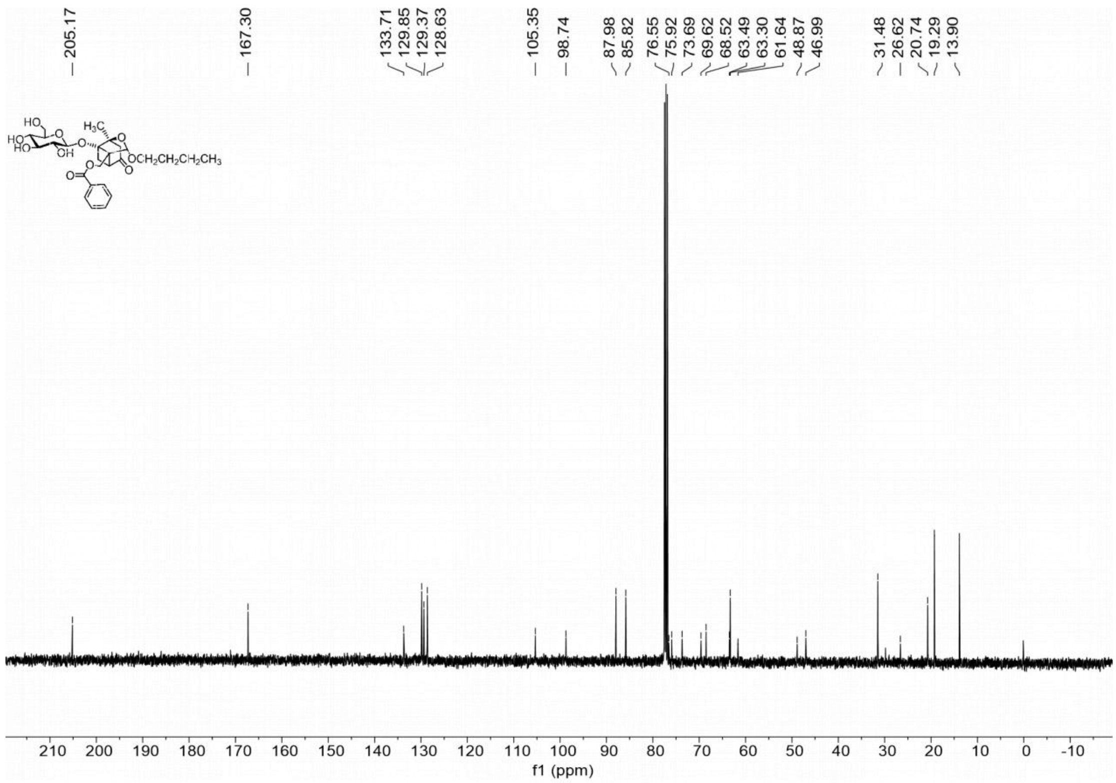

Figure S46. The ^{13}C NMR of compound **26**.

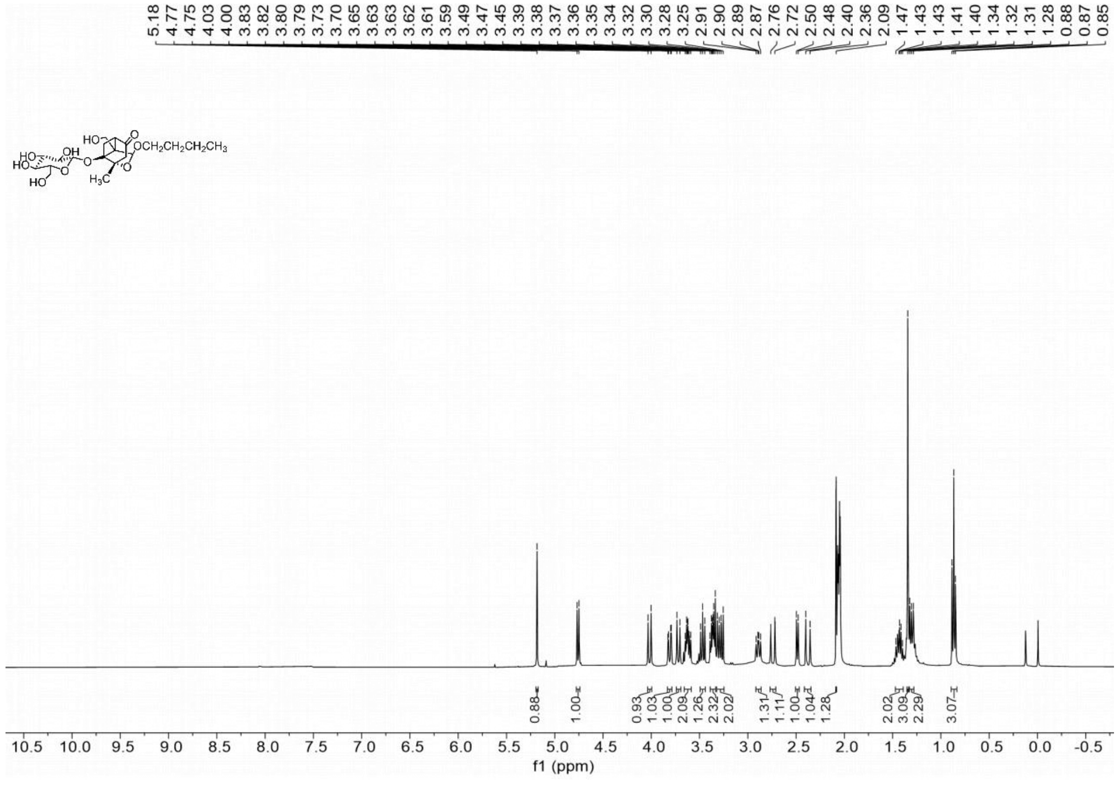

Figure S47. The ^{1}H NMR of compound **27**.

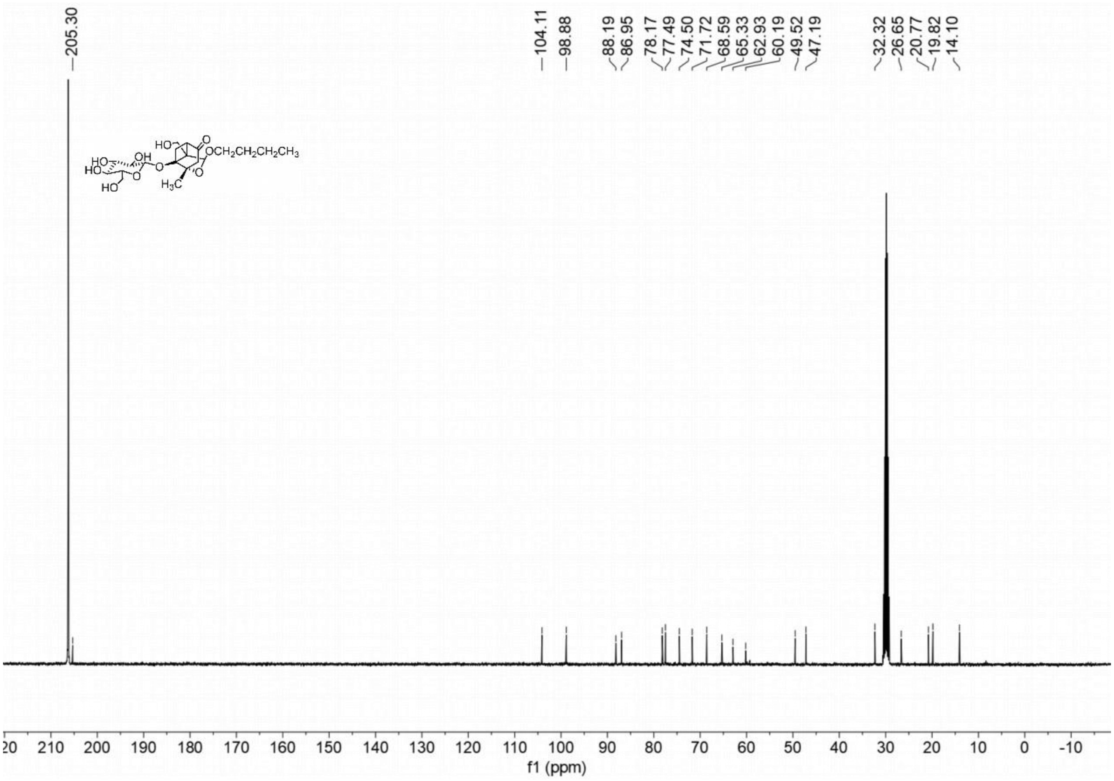

Figure S48. The ^{13}C NMR of compound **27**.

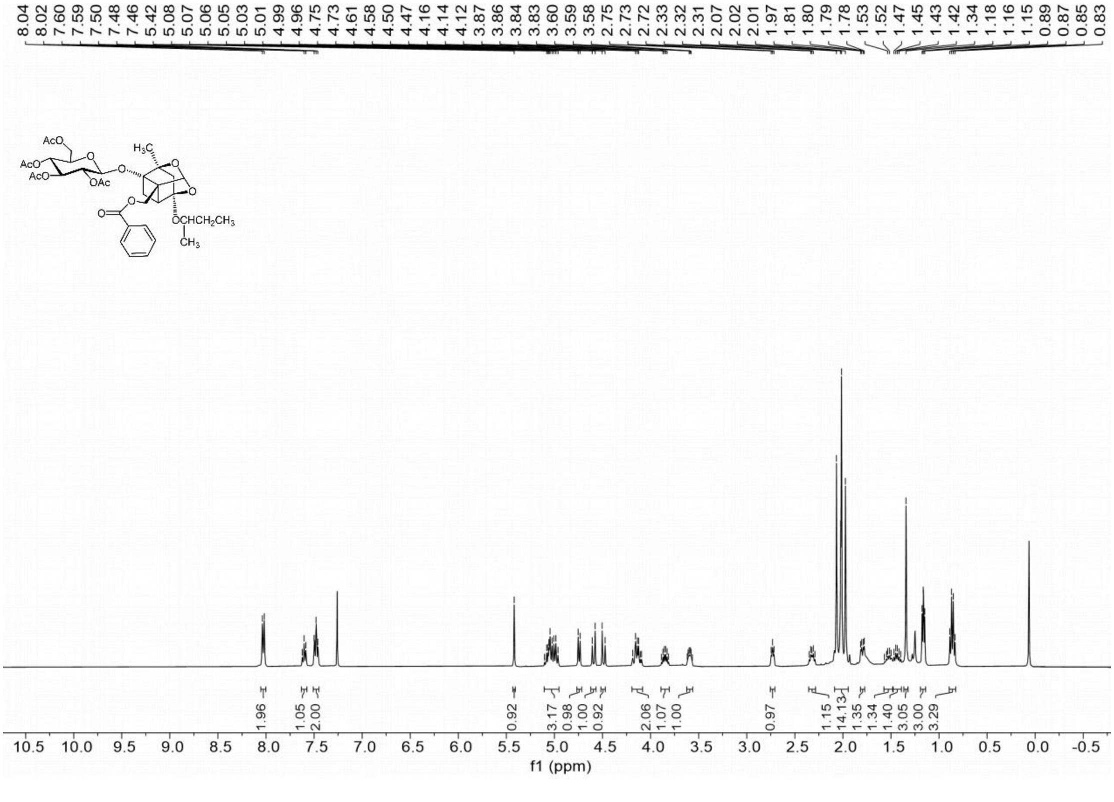

Figure S49. The ^{1}H NMR of compound **28**.

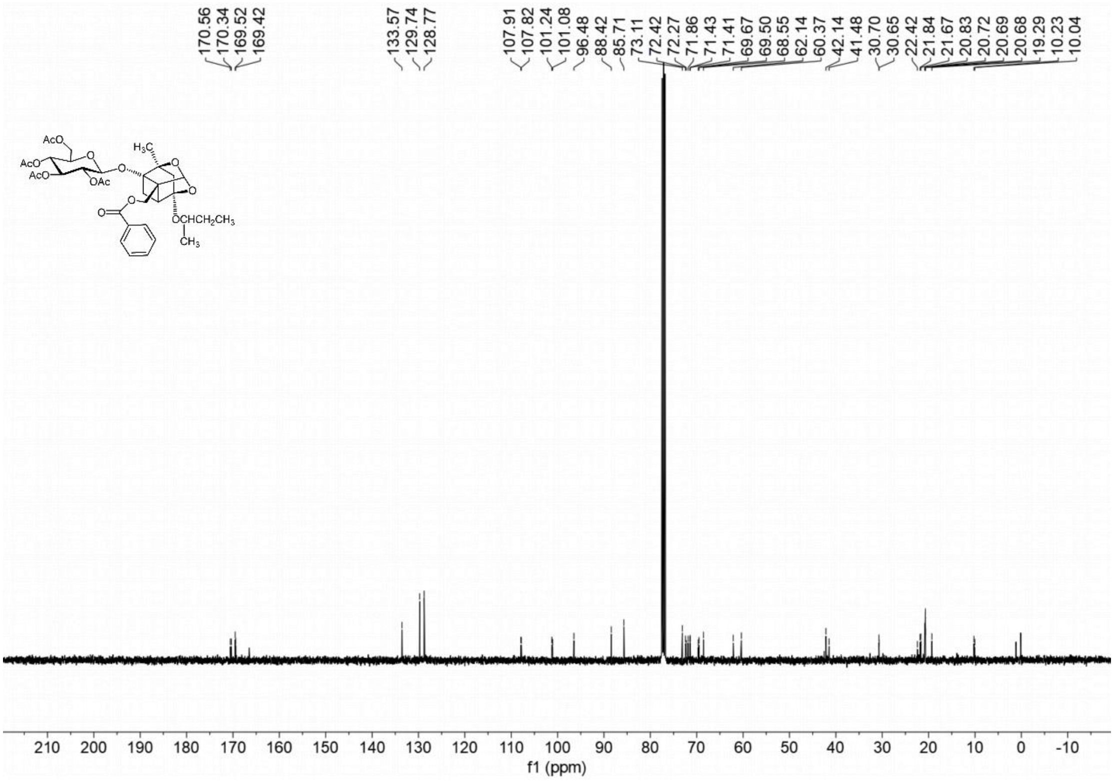

Figure S50. The ^{13}C NMR of compound **28**.

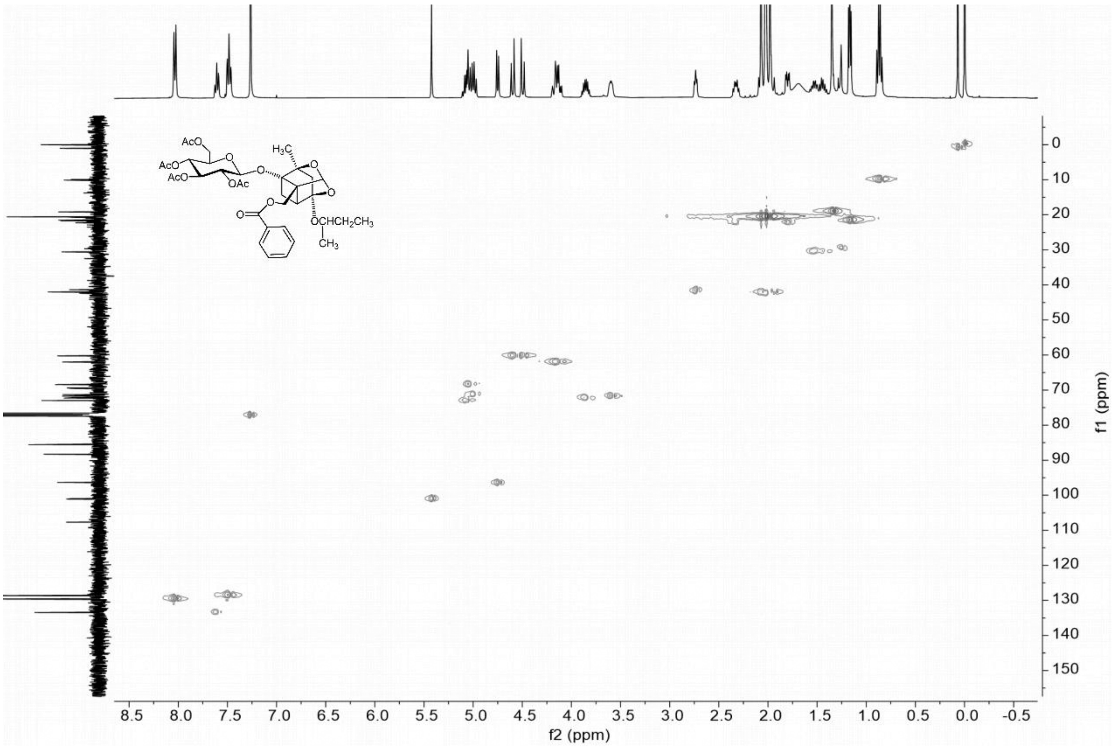

Figure S51. The HSQC-NMR of compound **28**.

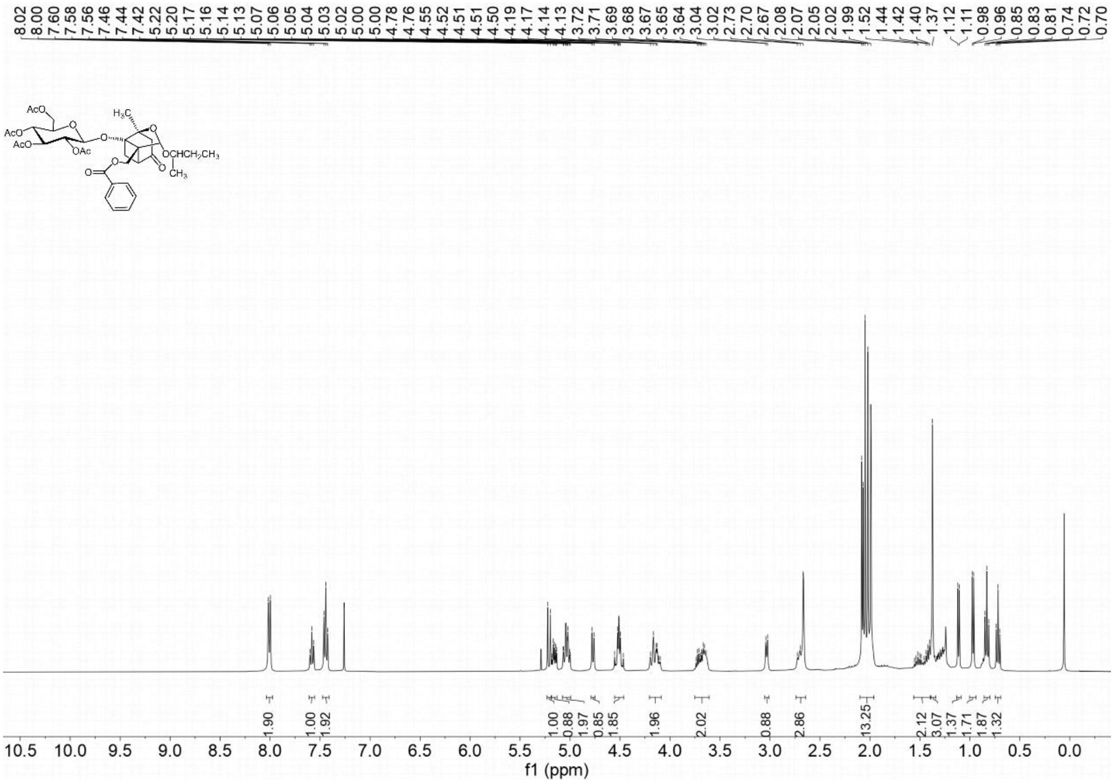

Figure S52. The ^{1}H NMR of compound **29**.

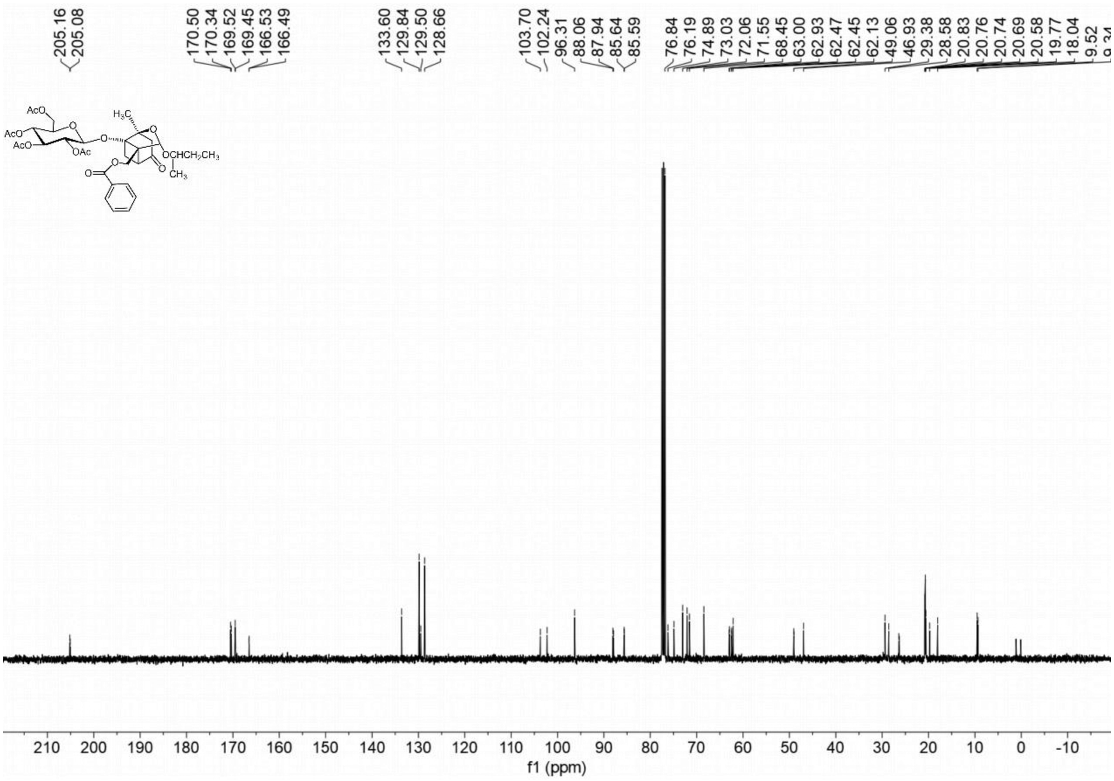

Figure S53. The ^{13}C NMR of compound **29**.

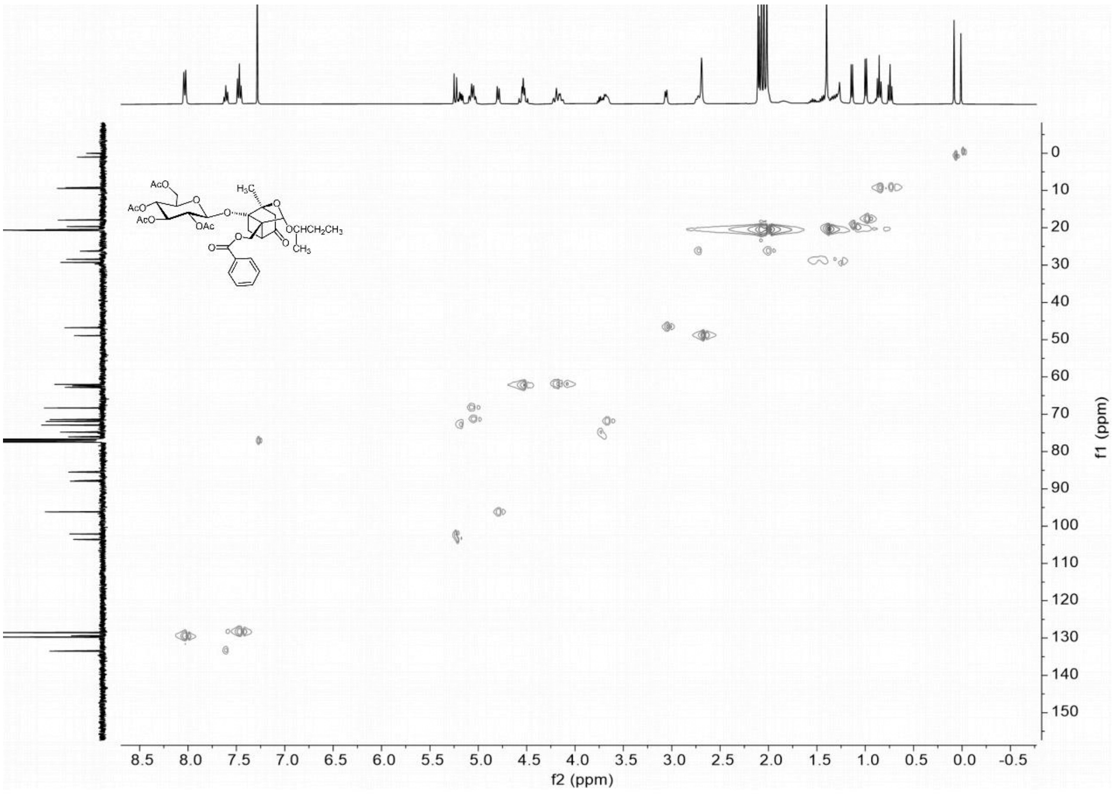

Figure S54. The HSQC-NMR of compound **29**.

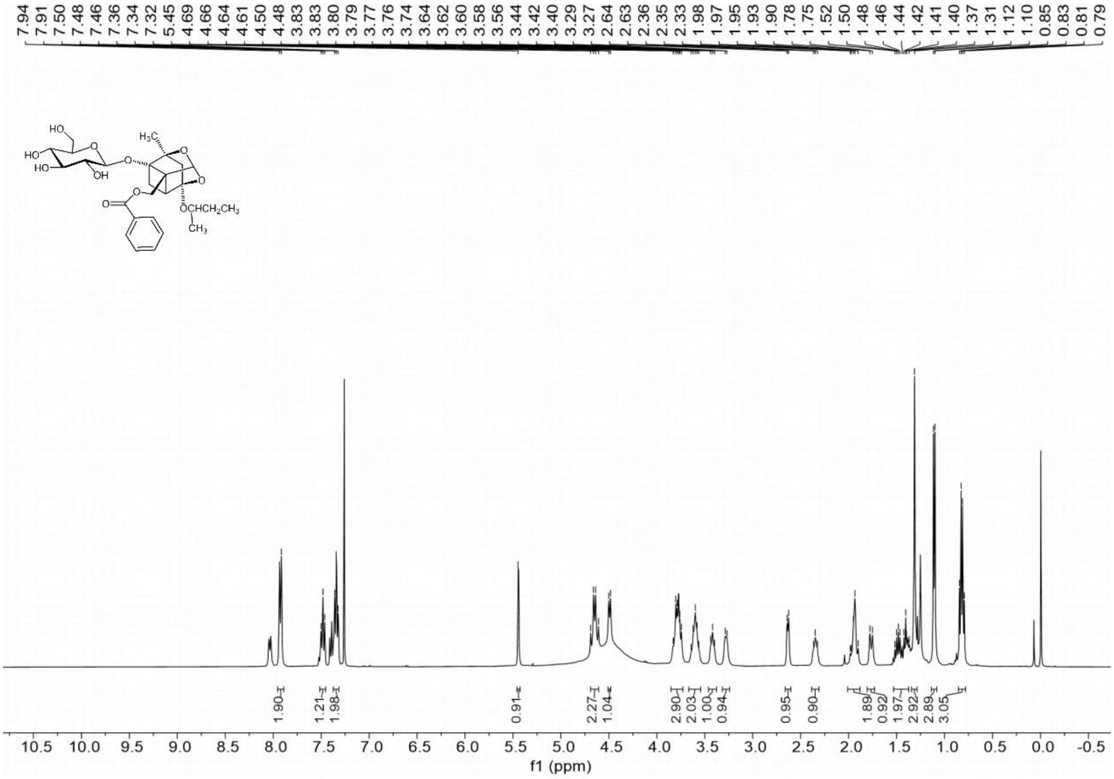

Figure S55. The ^{1}H NMR of compound **30**.

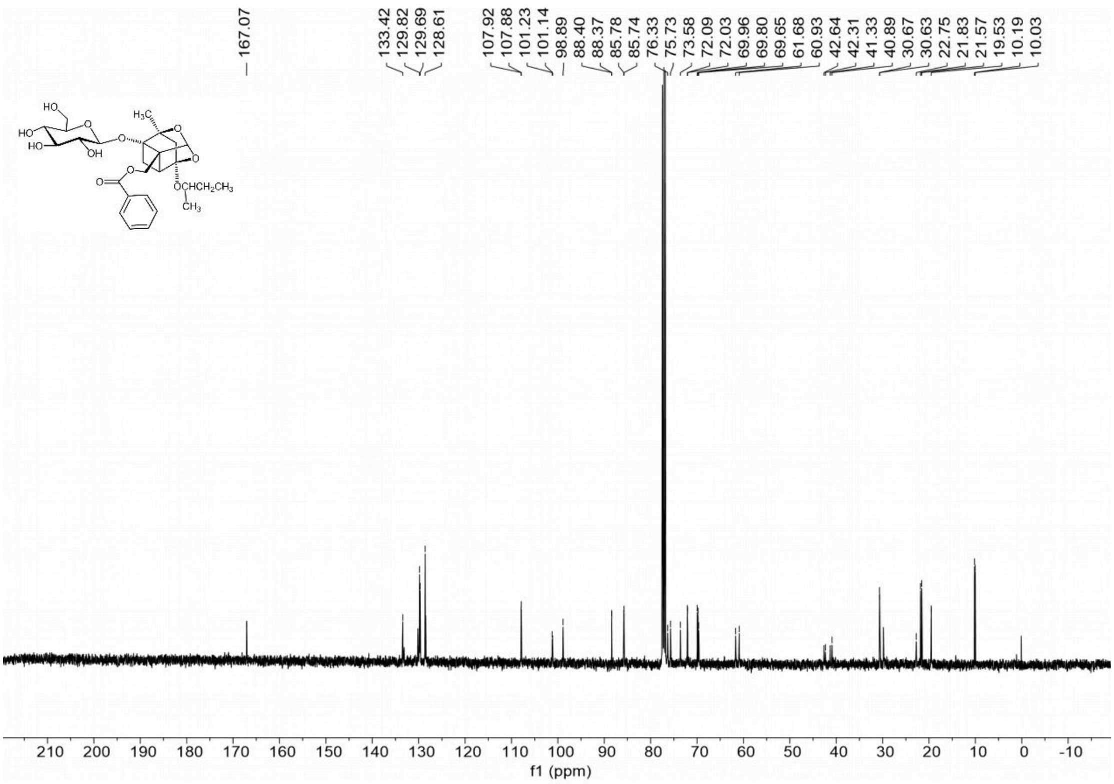

Figure S56. The ^{13}C NMR of compound **30**.

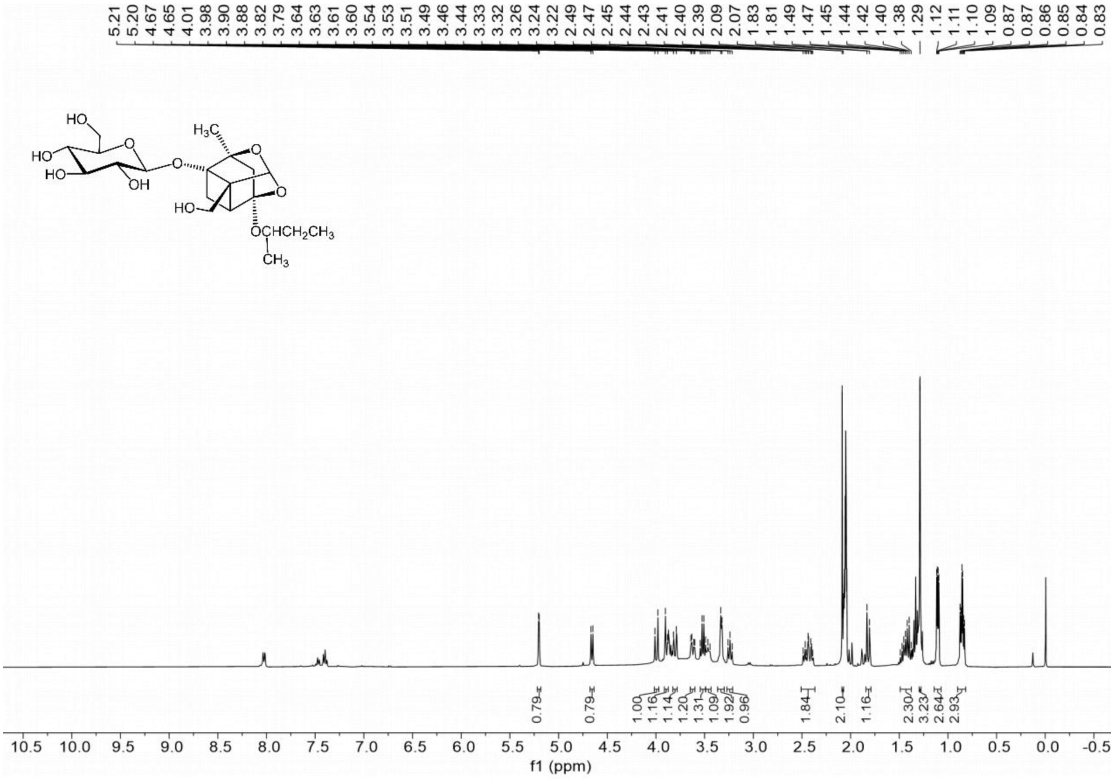

Figure S57. The ^1H NMR of compound **31**.

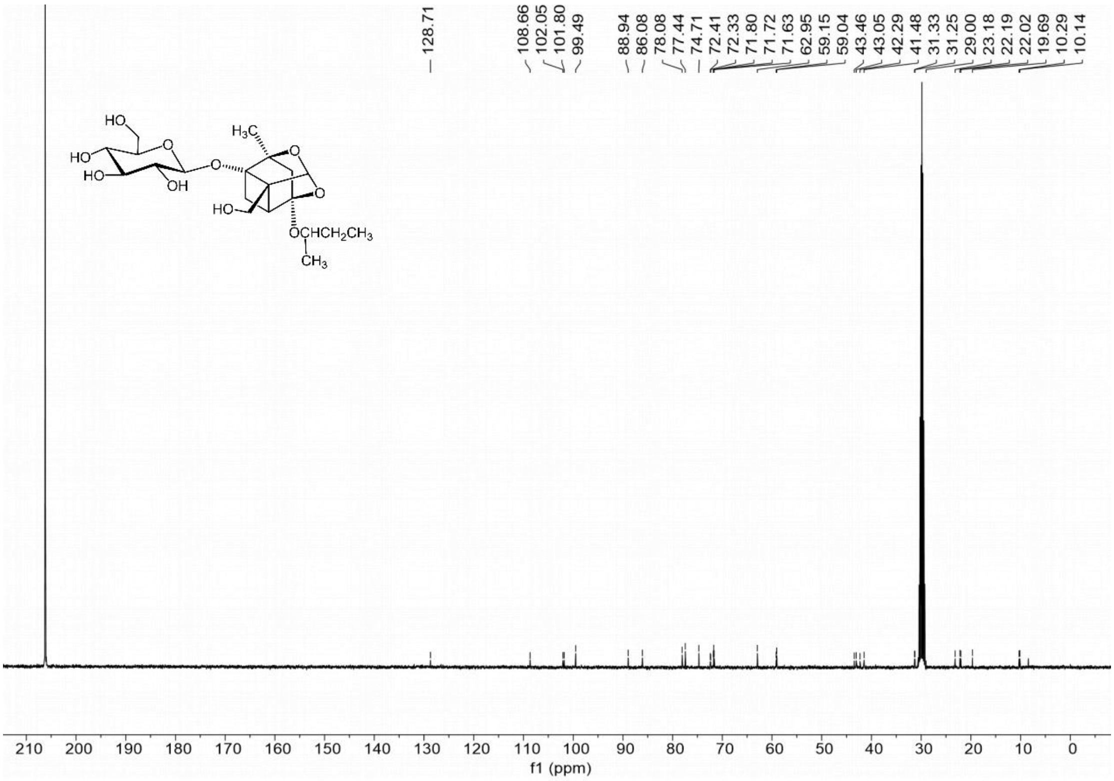

Figure S58. The ^{13}C NMR of compound **31**.

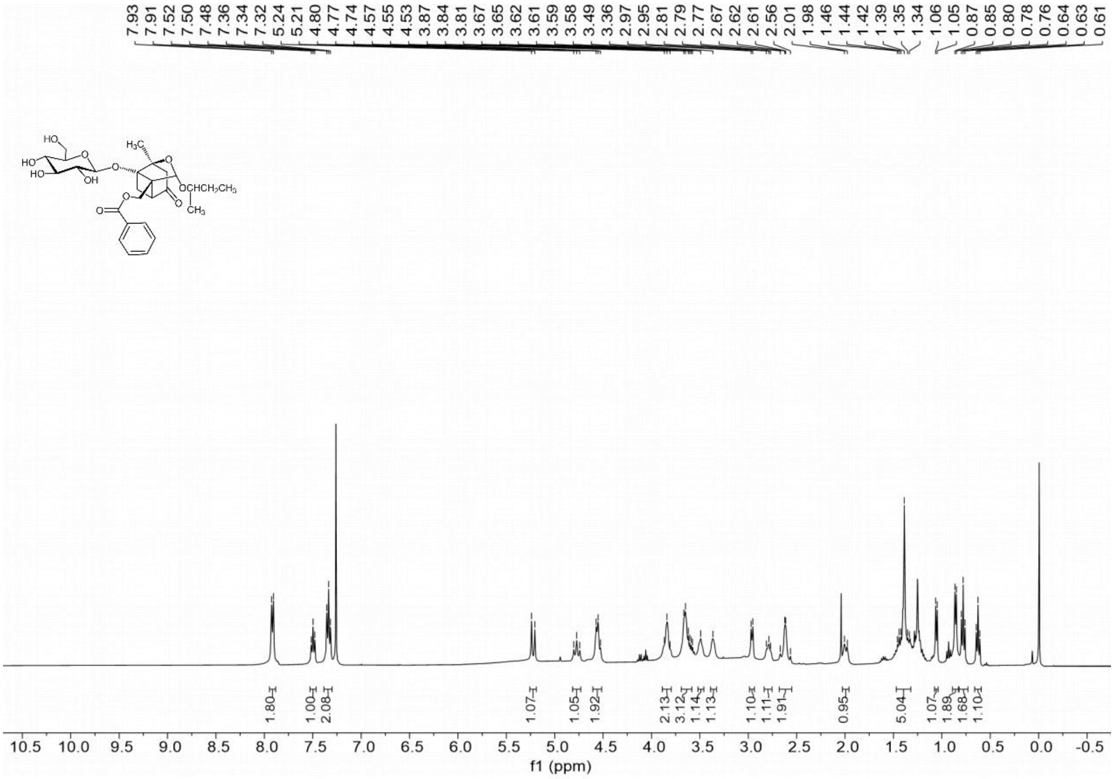

Figure S59. The ^{1}H NMR of compound **32**.

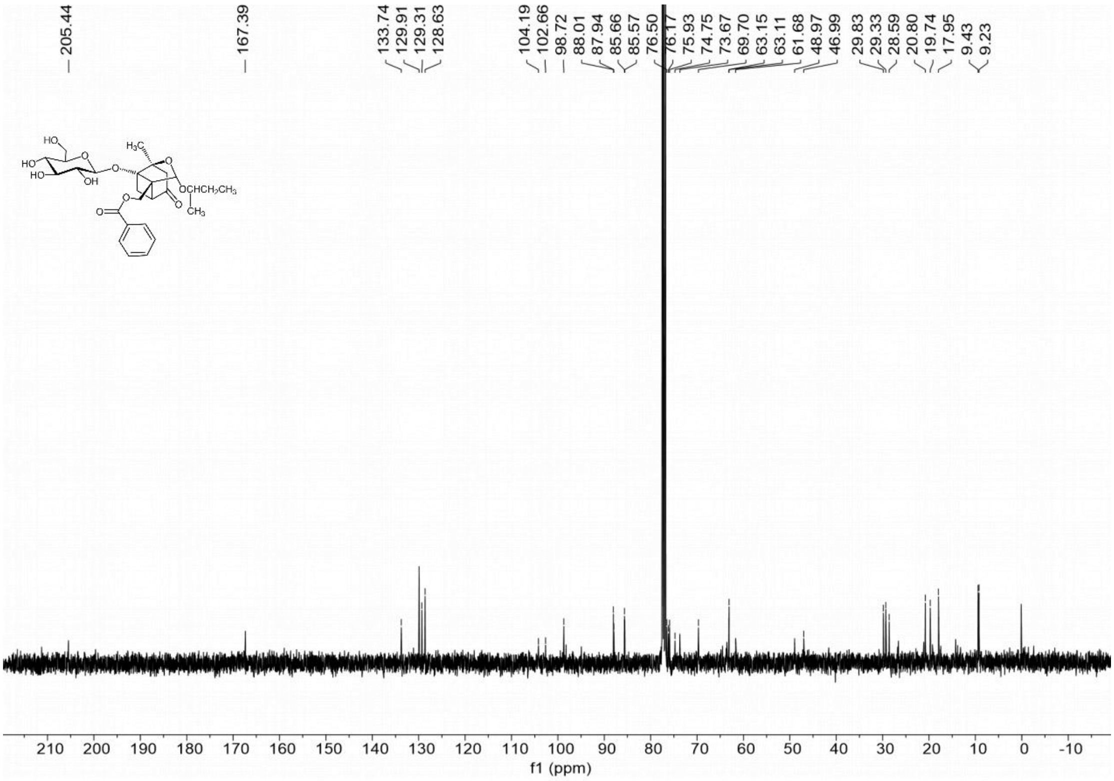

Figure S60. The ^{13}C NMR of compound **32**.

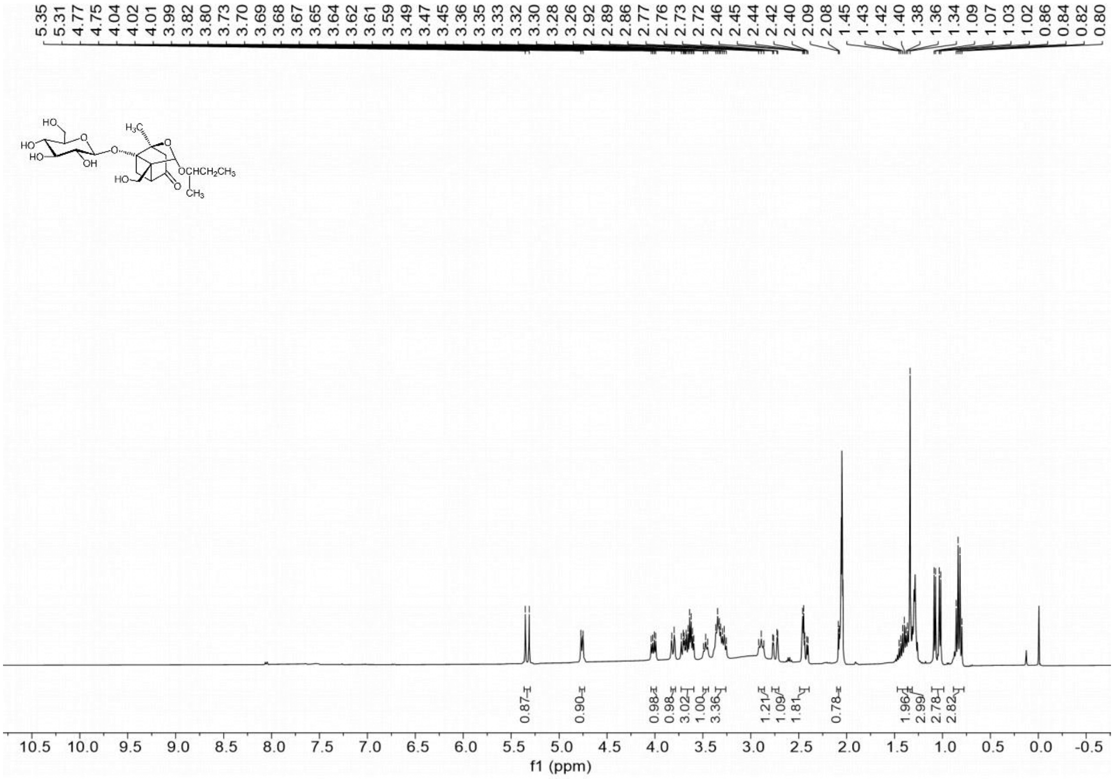

Figure S61. The ^{1}H NMR of compound **33**.

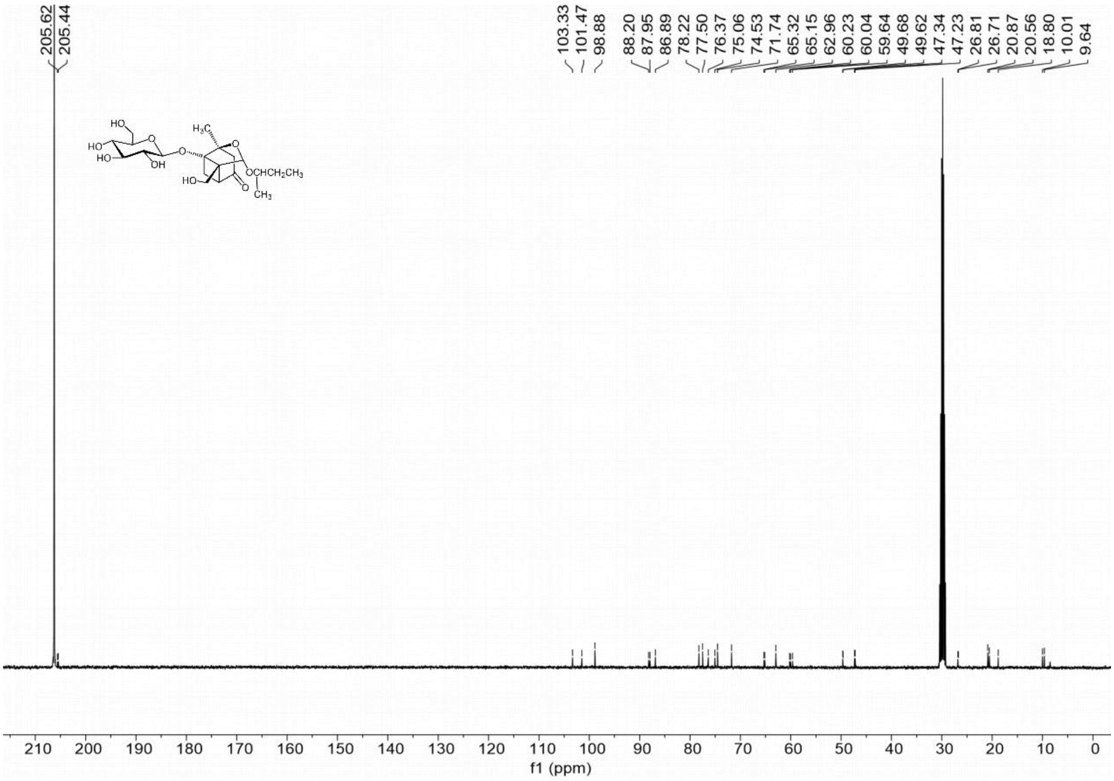

Figure S62. The ^{13}C NMR of compound **33**.

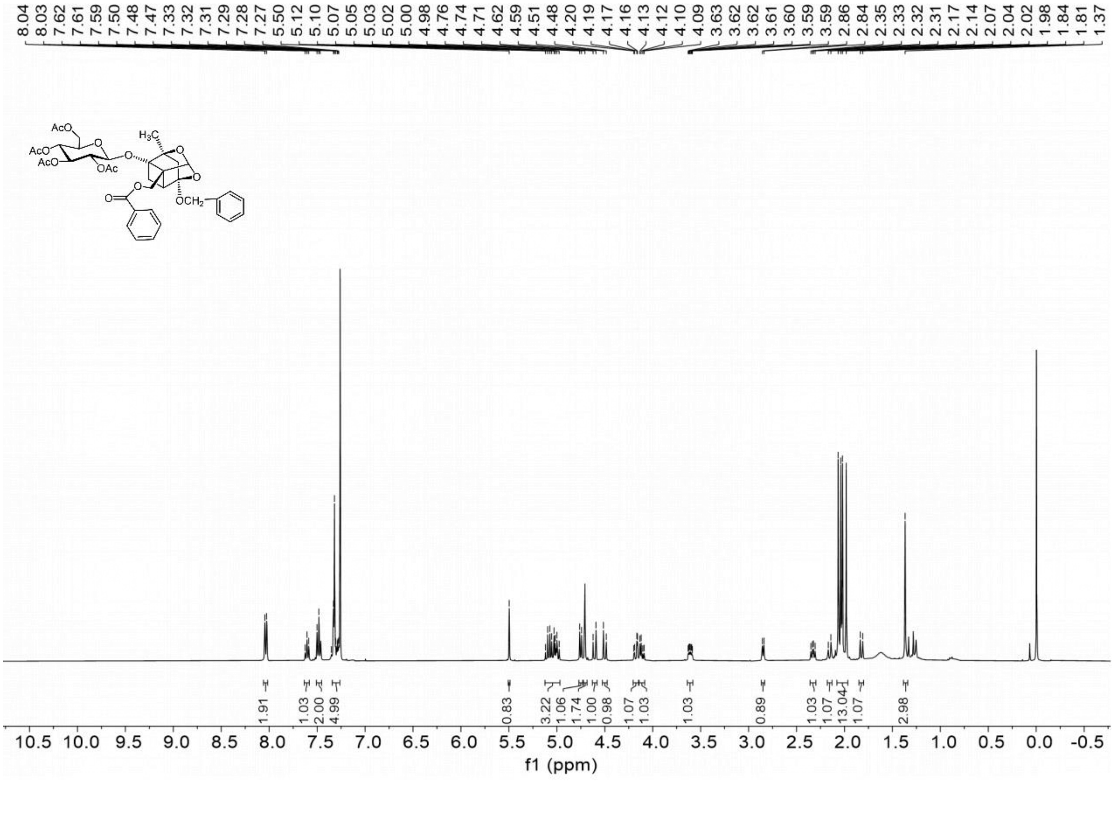

Figure S63. The ^{1}H NMR of compound **34**.

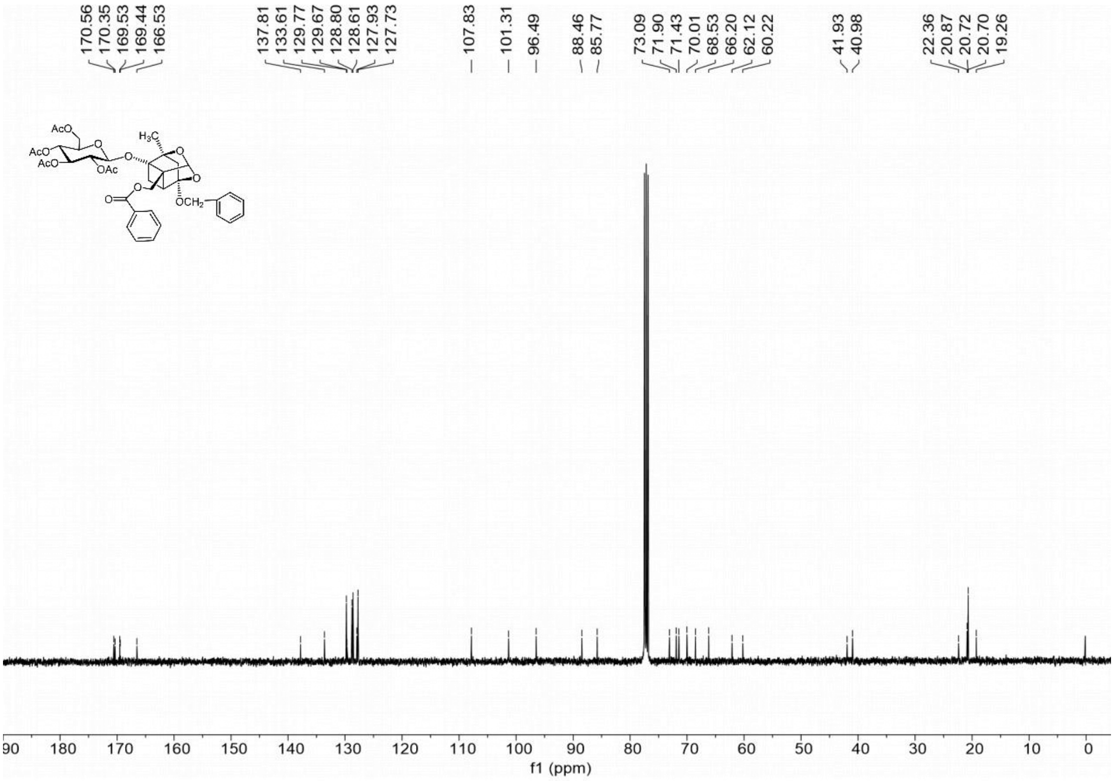

Figure S64. The ^{13}C NMR of compound **34**.

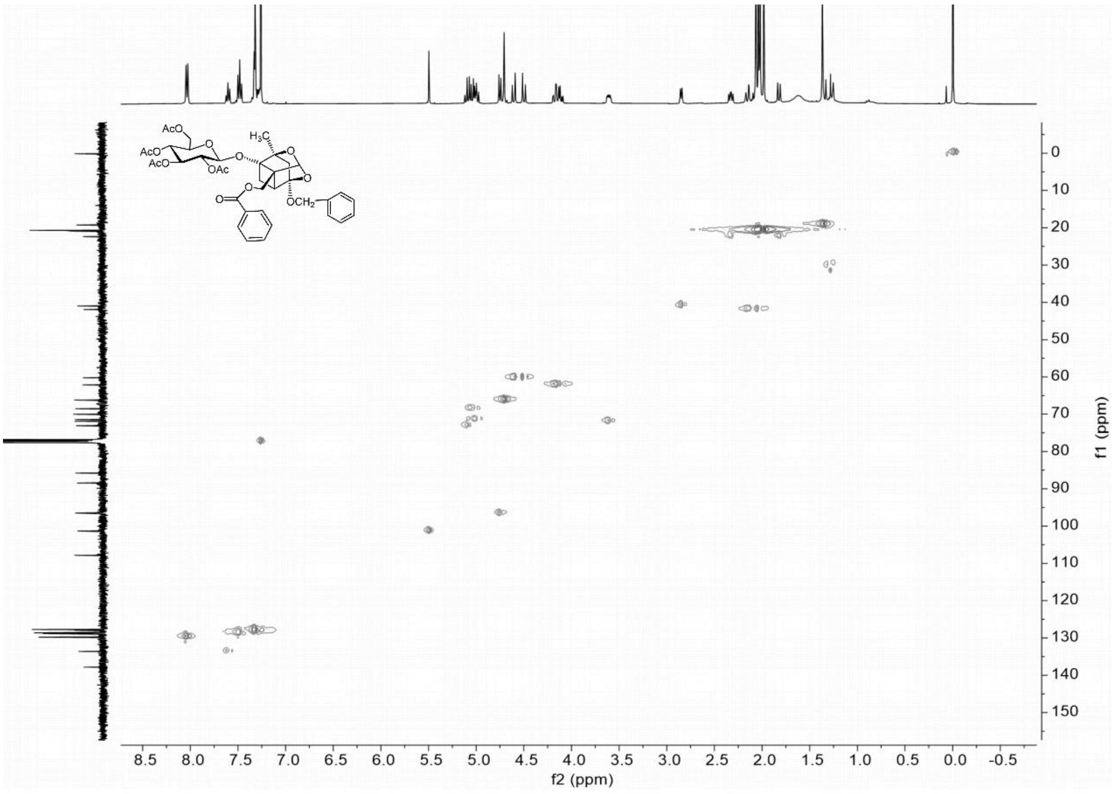

Figure S65. The HSQC-NMR of compound **34**.

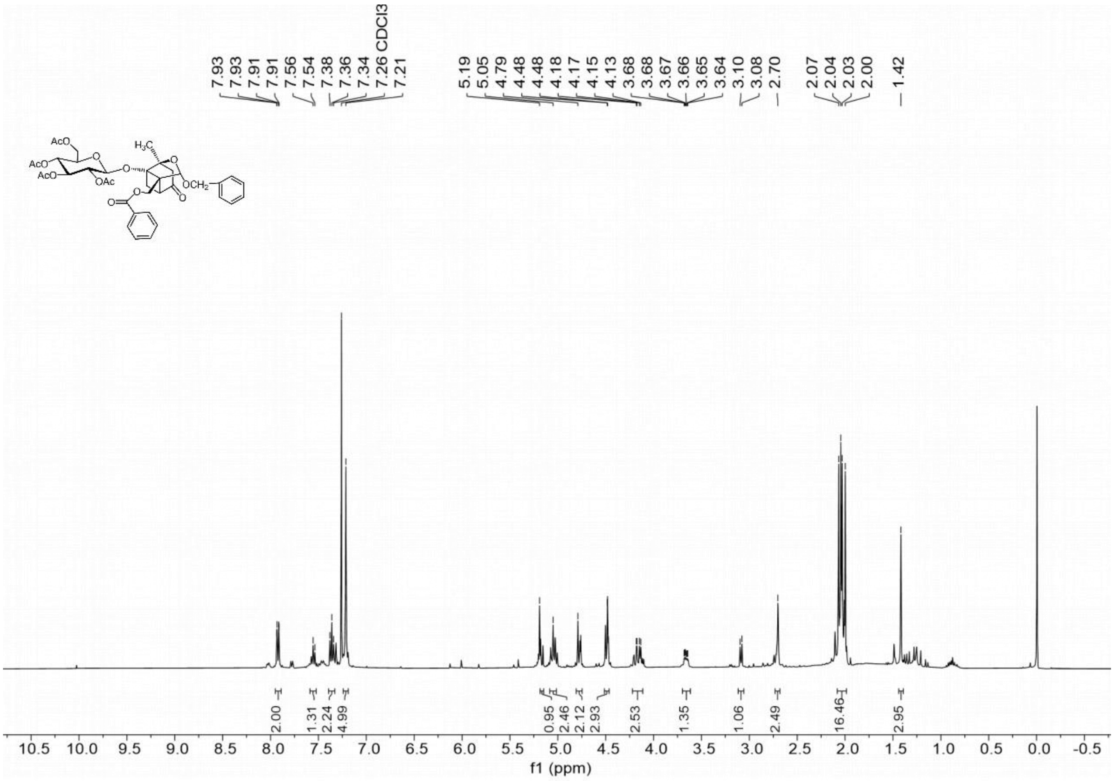

Figure S66. The ^{1}H NMR of compound **35**.

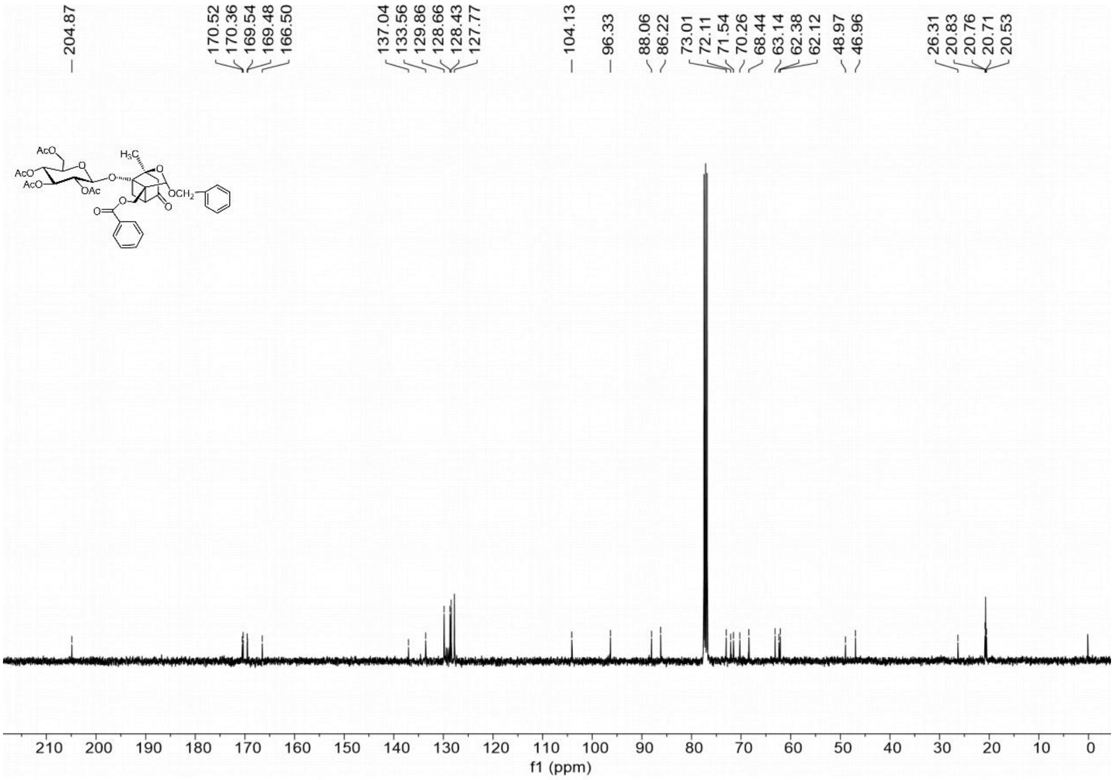

Figure S67. The ^{13}C NMR of compound **35**.

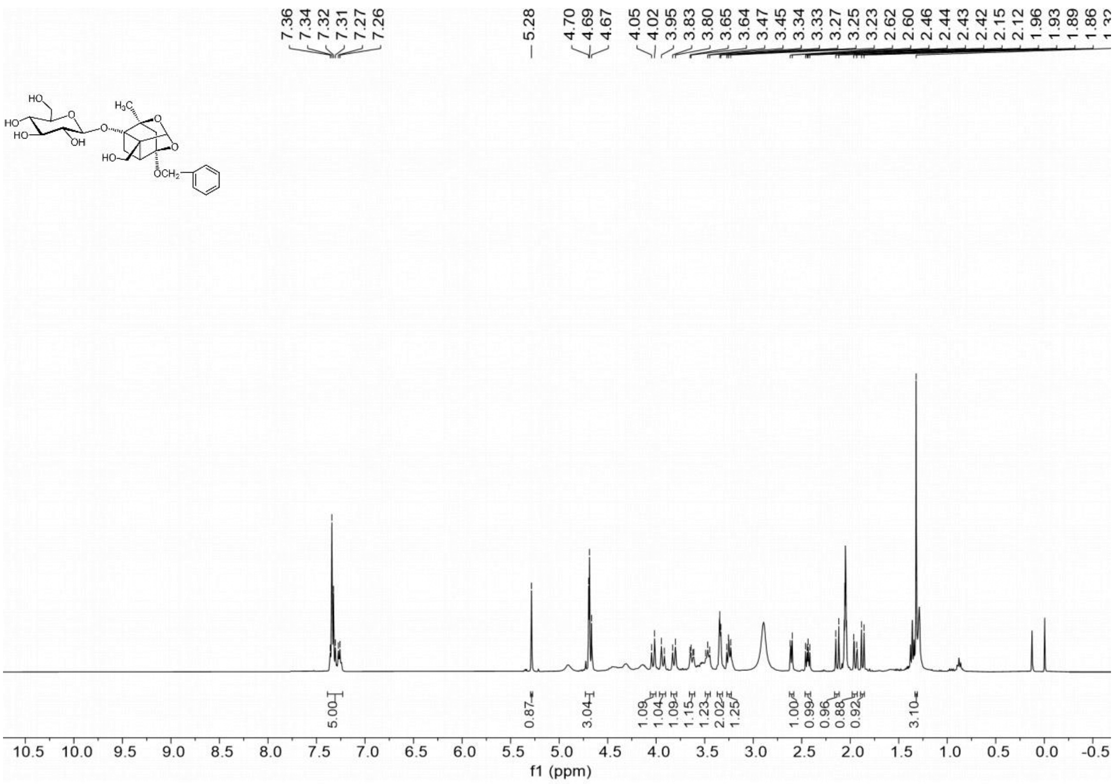

Figure S68. The ^{1}H NMR of compound **36**.

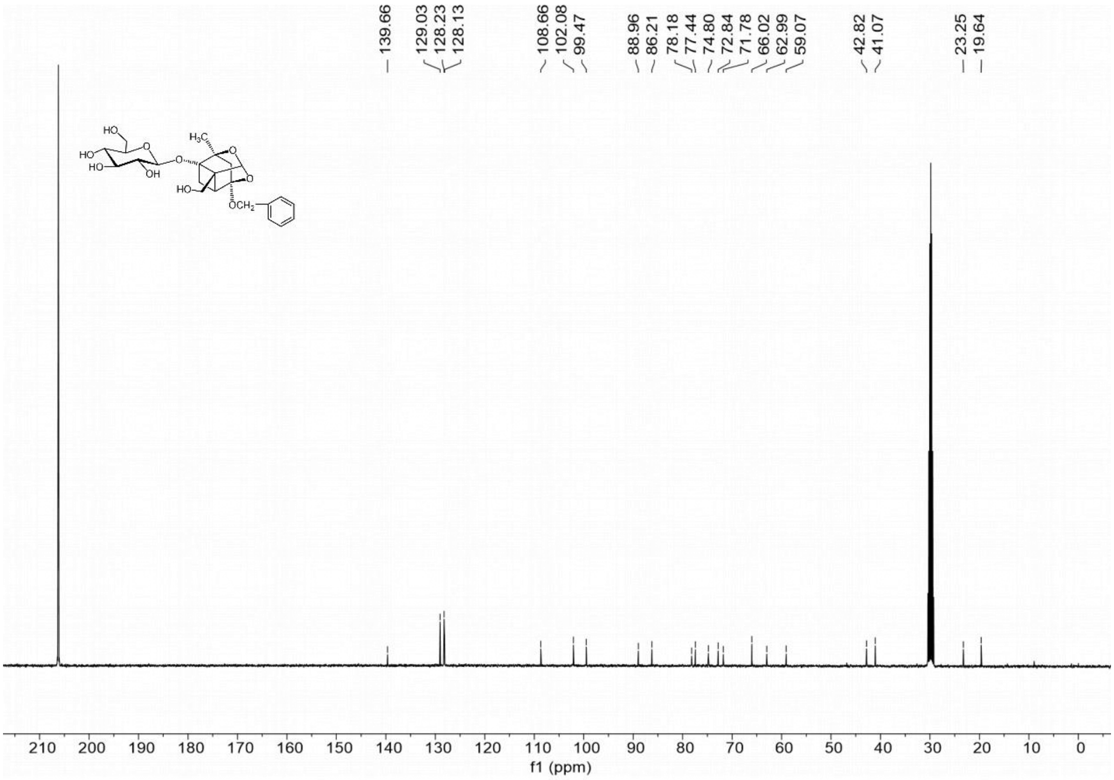

Figure S69. The ^{13}C NMR of compound **36**.

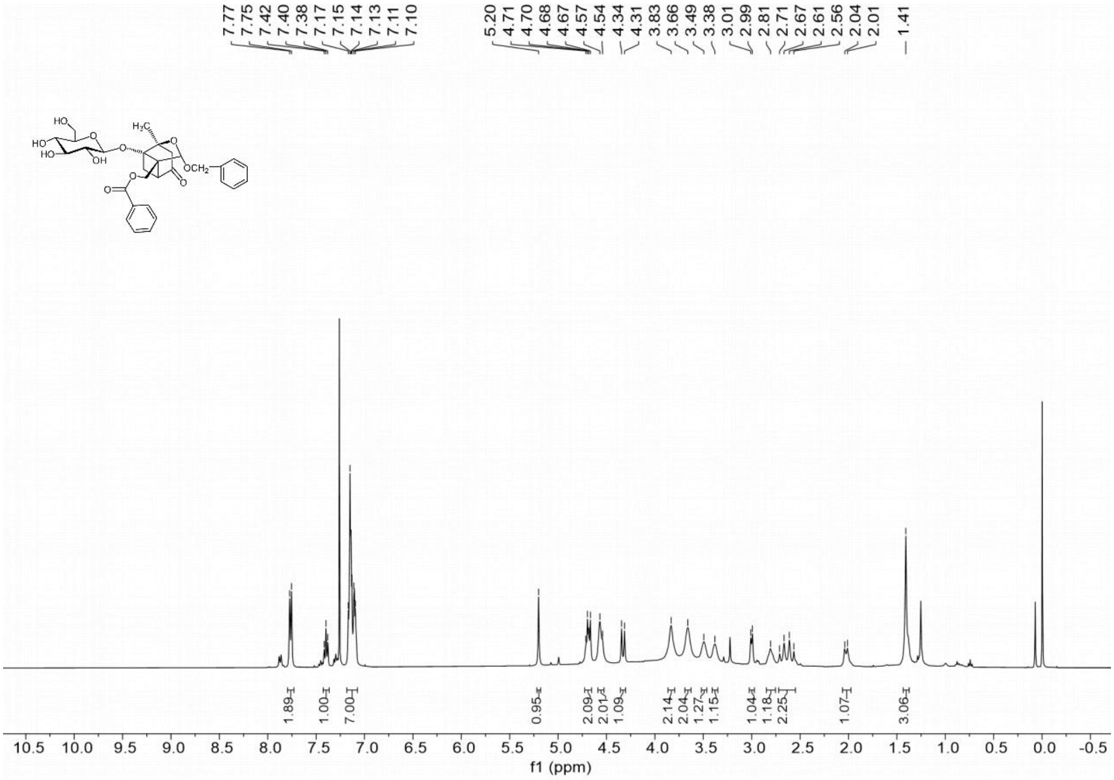

Figure S70. The ^{1}H NMR of compound **37**.

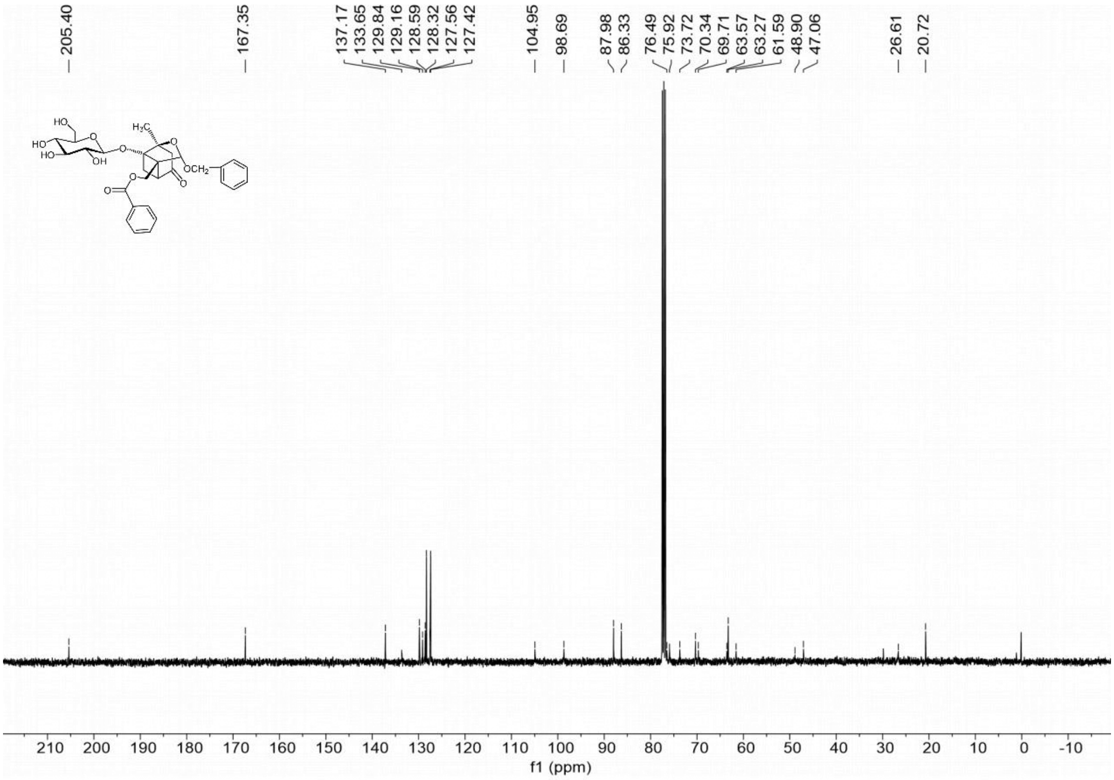

Figure S71. The ^{13}C NMR of compound **37**.

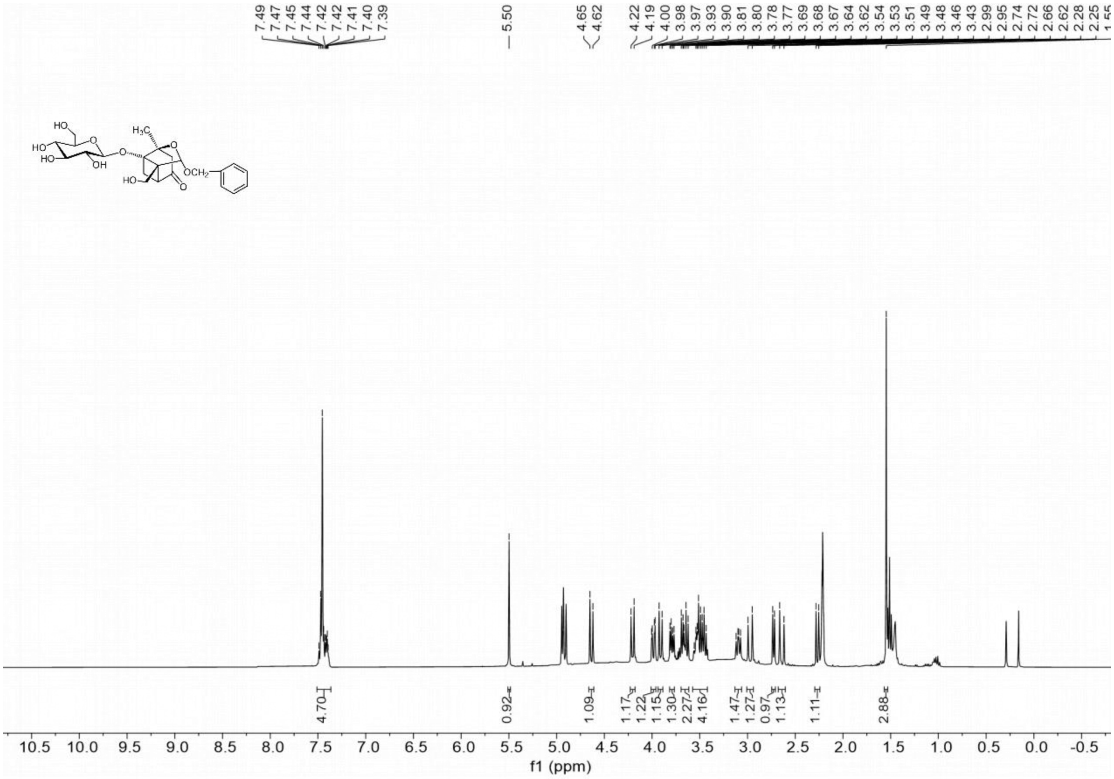

Figure S72. The ^{1}H NMR of compound **38**.

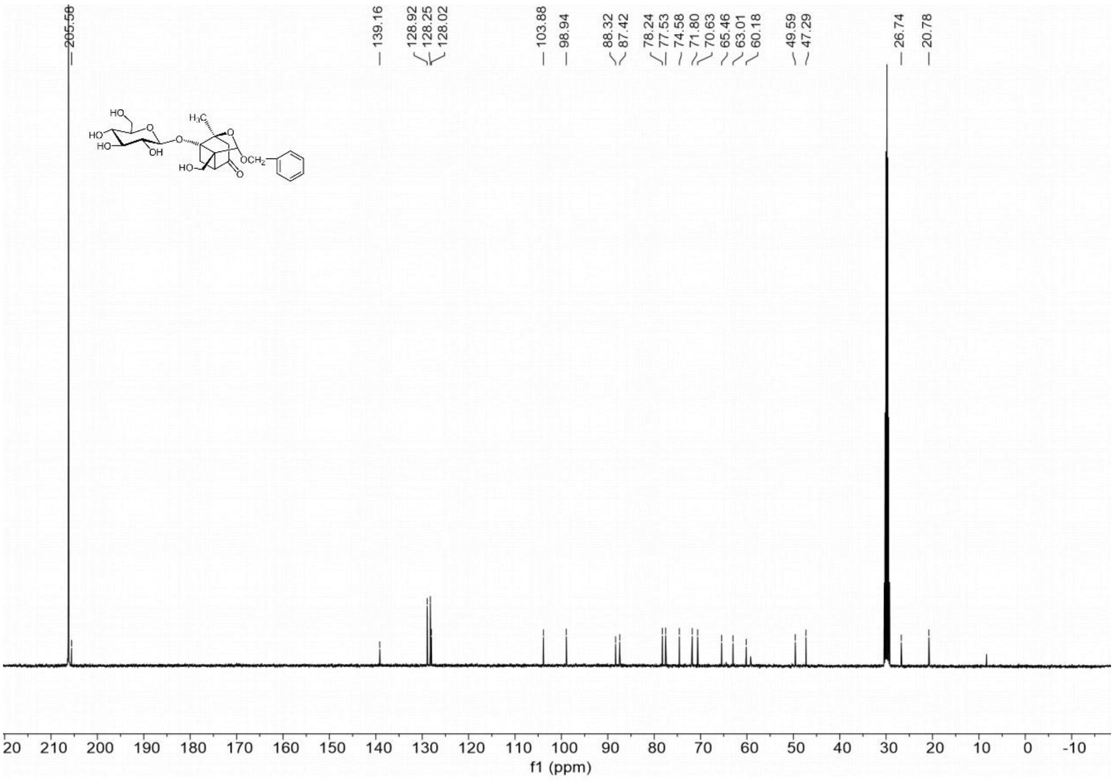

Figure S73. The ^{13}C NMR of compound **38**.

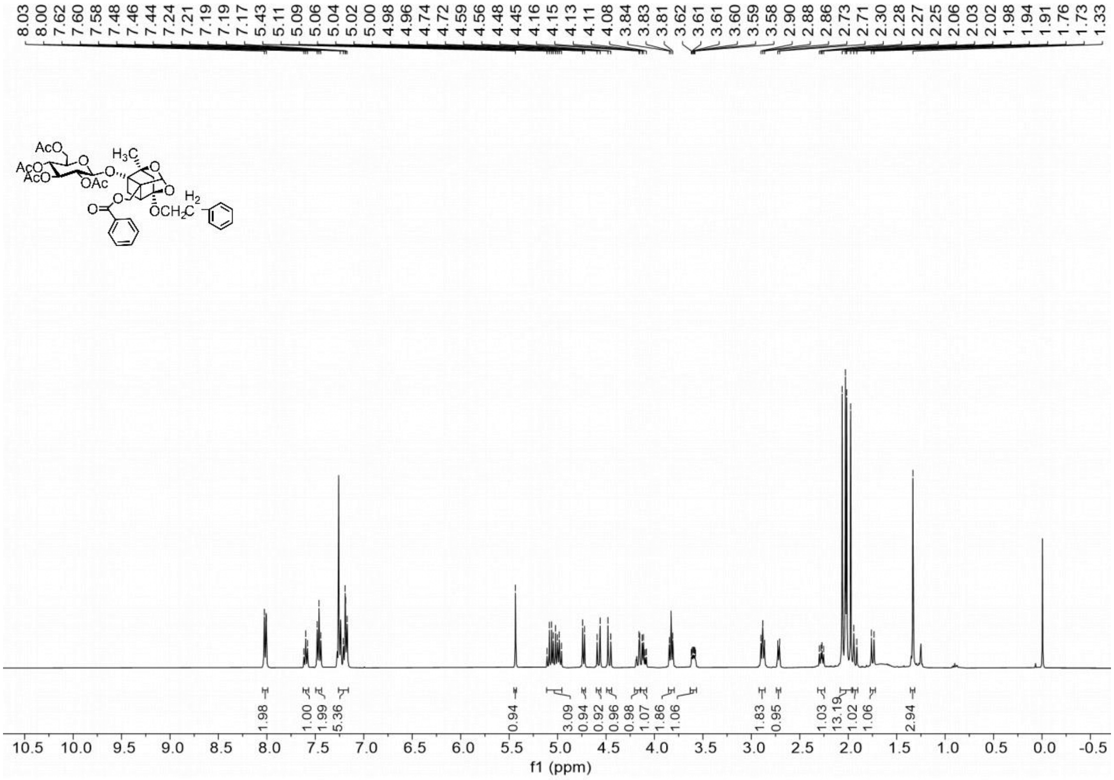

Figure S74. The ^{1}H NMR of compound **39**.

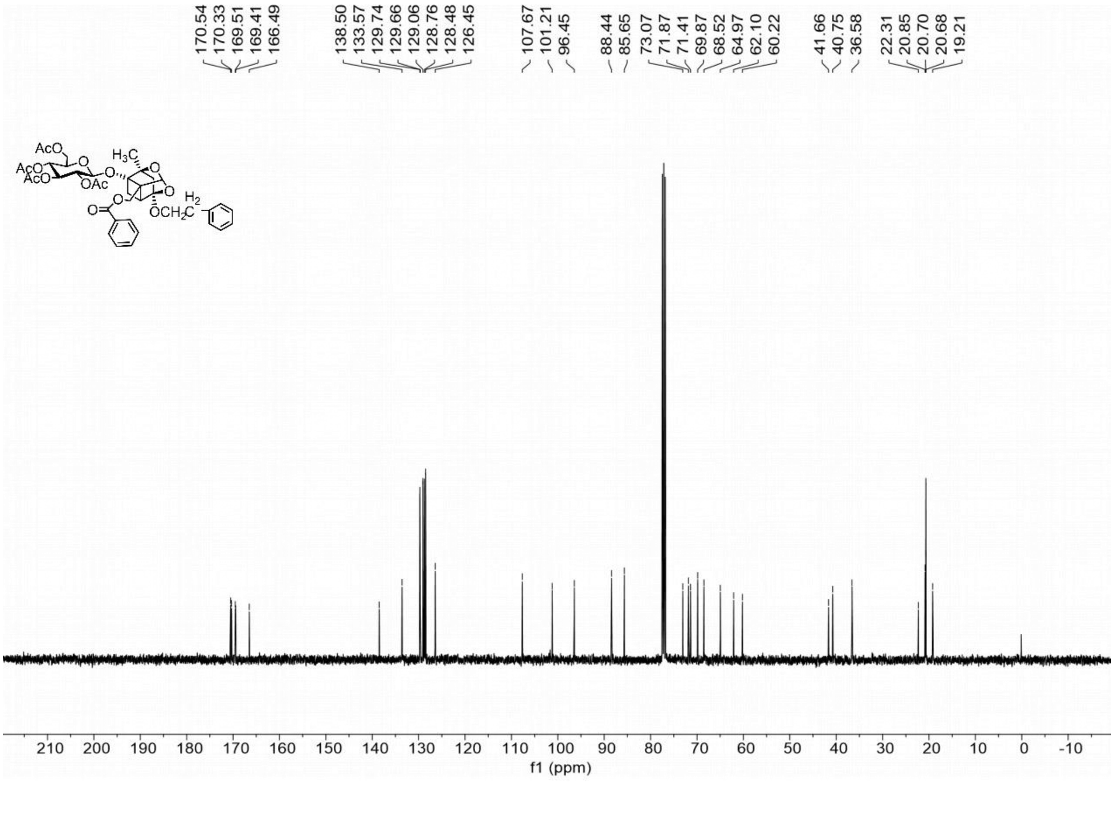

Figure S75. The ^{13}C NMR of compound **39**.

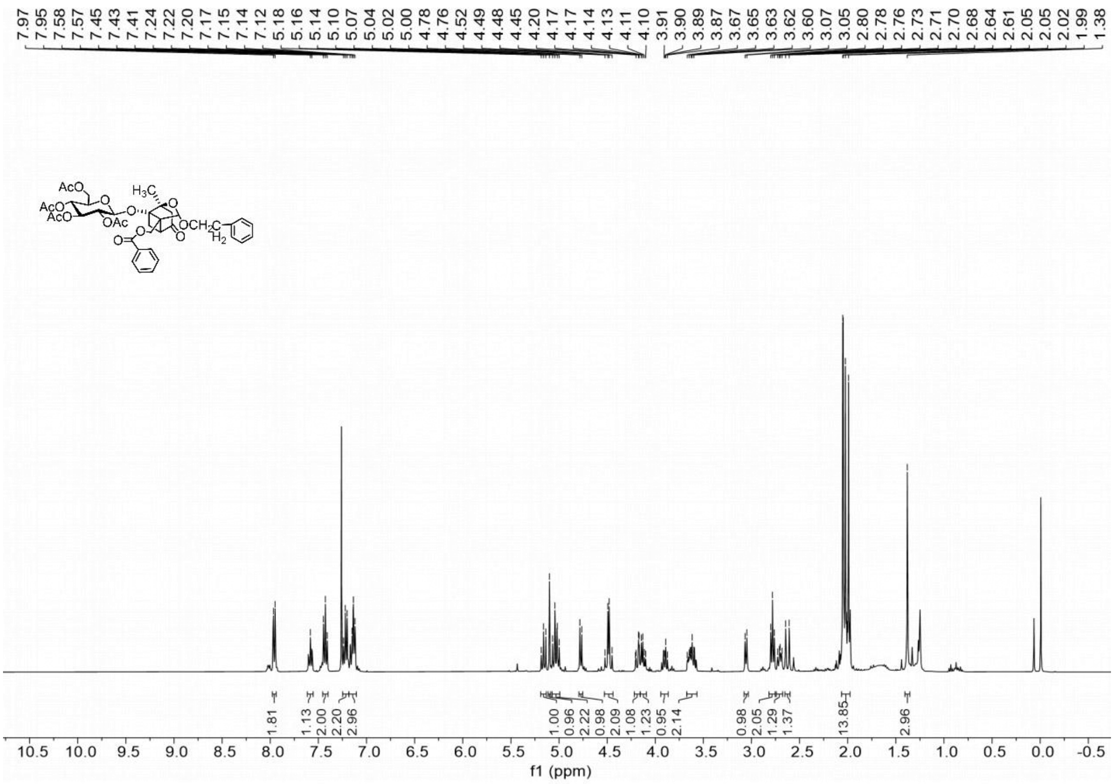

Figure S76. The ^{1}H NMR of compound **40**.

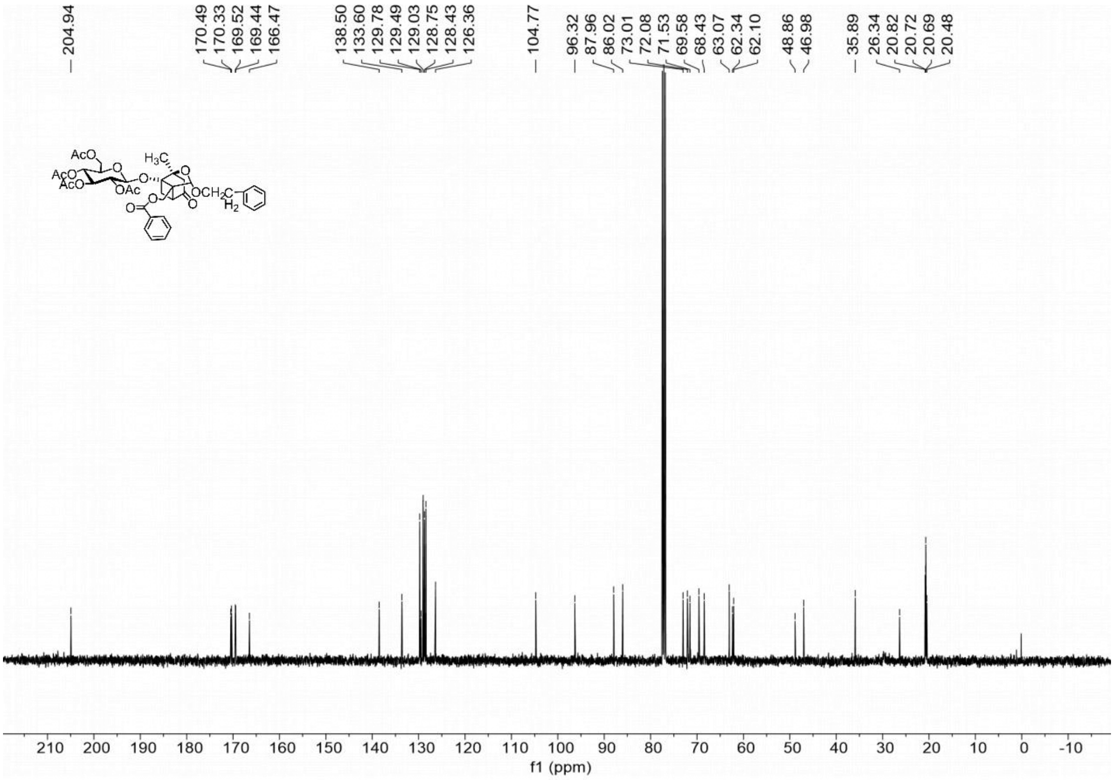

Figure S77. The ^{13}C NMR of compound **40**.

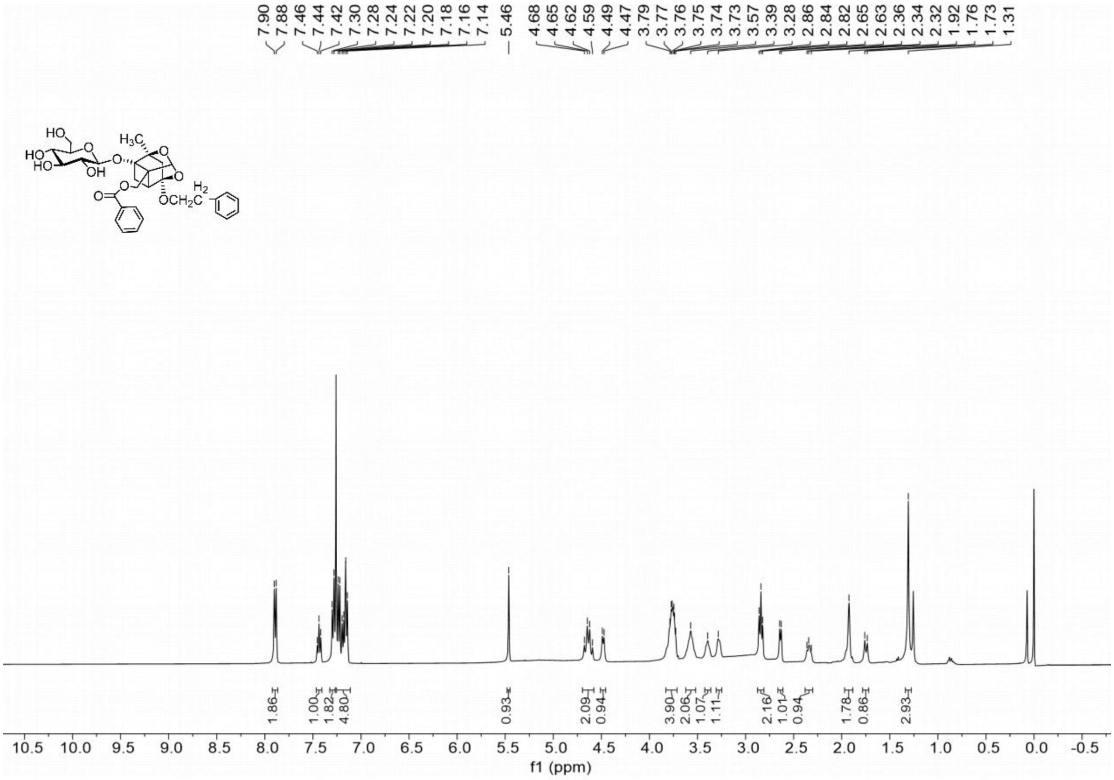

Figure S78. The ^{1}H NMR of compound **41**.

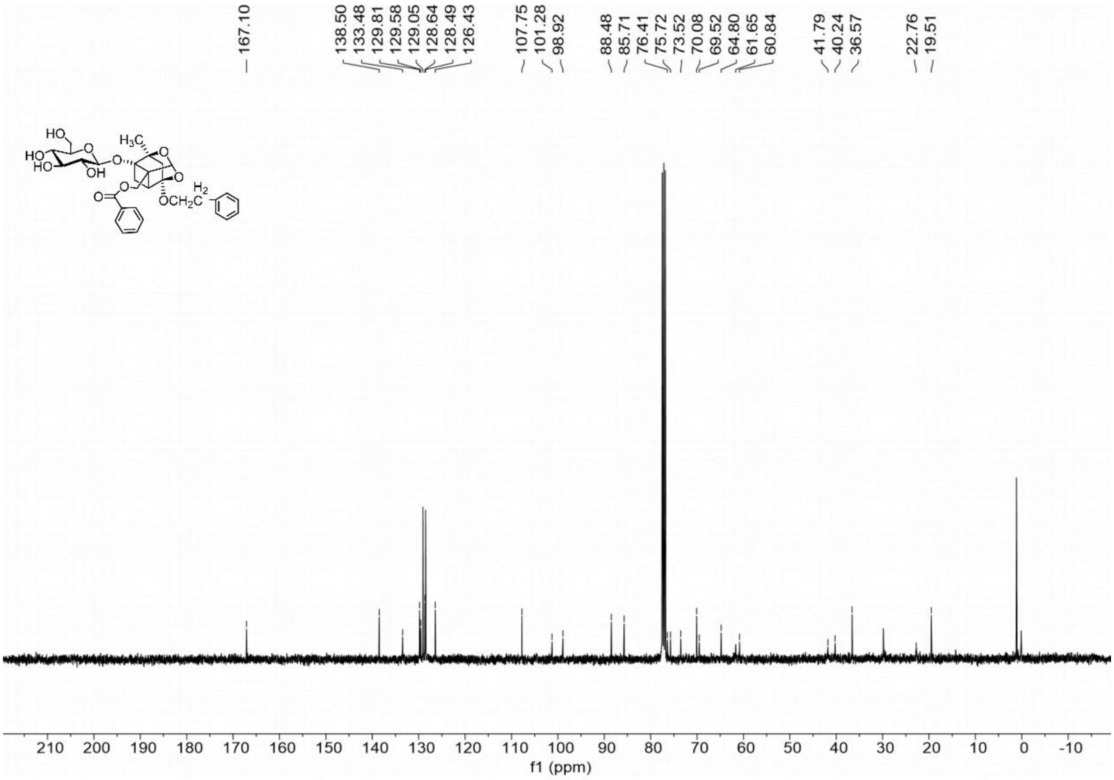

Figure S79. The ^{13}C NMR of compound **41**.

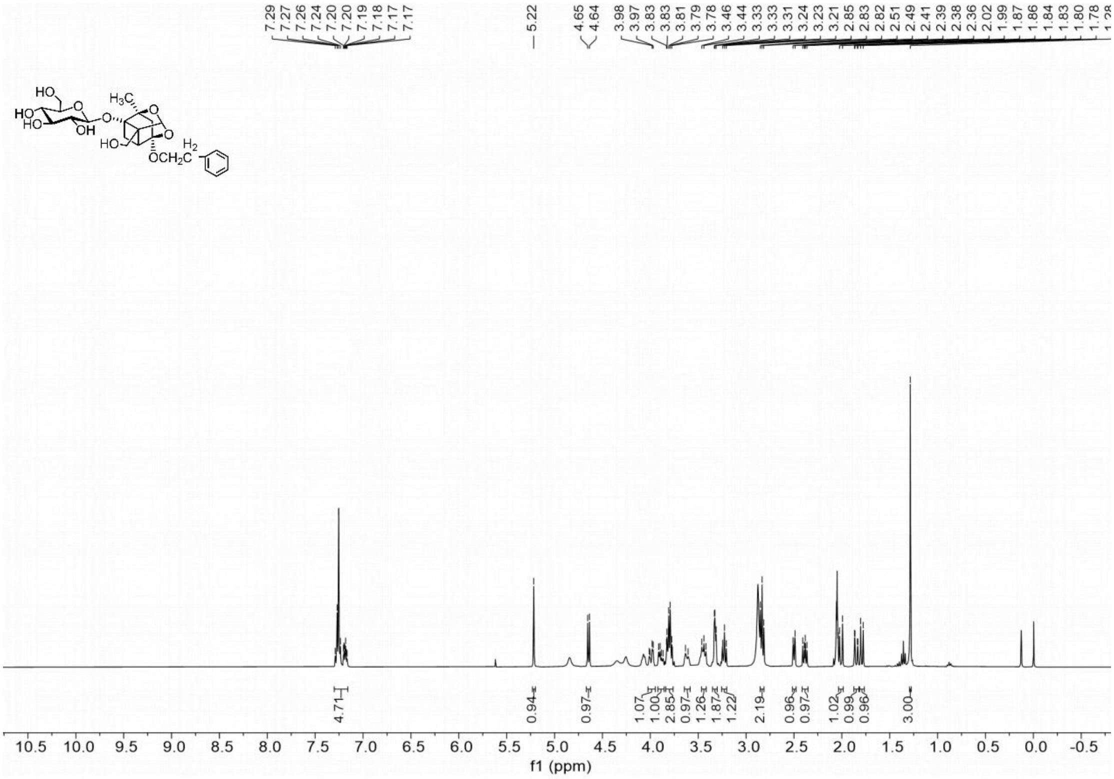

Figure S80. The ^{1}H NMR of compound **42**.

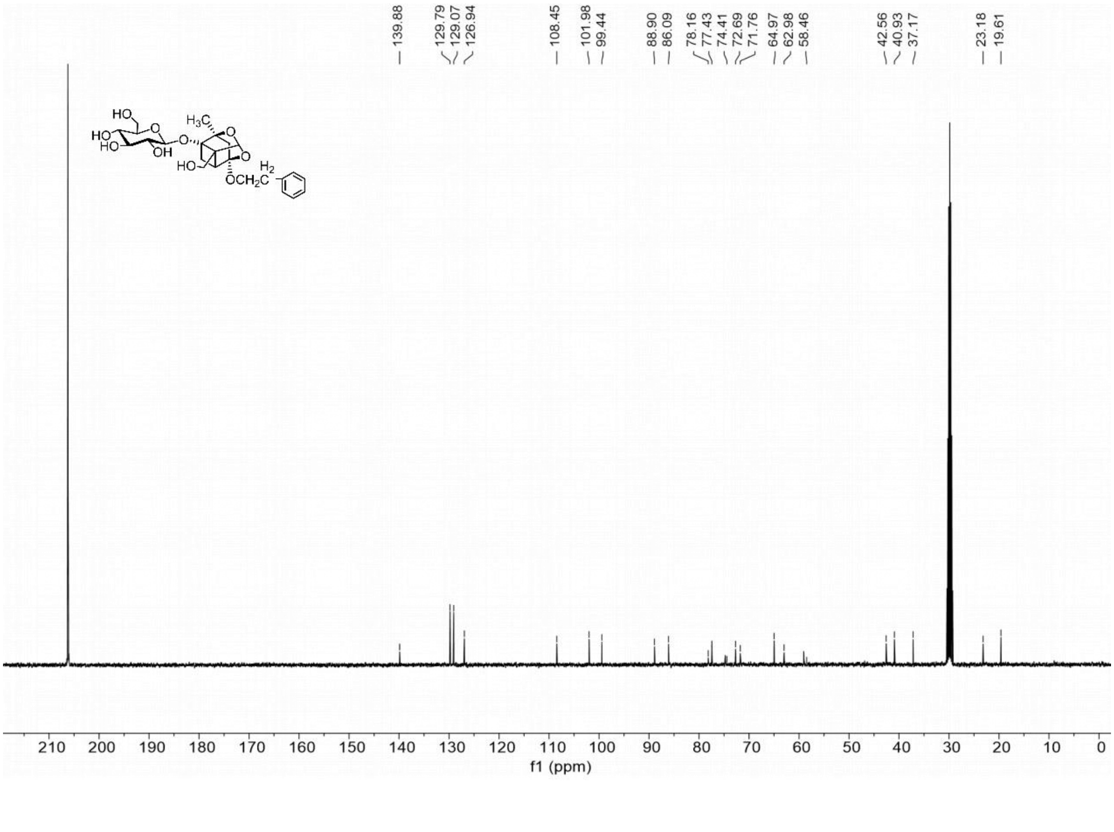

Figure S81. The ^{13}C NMR of compound **42**.

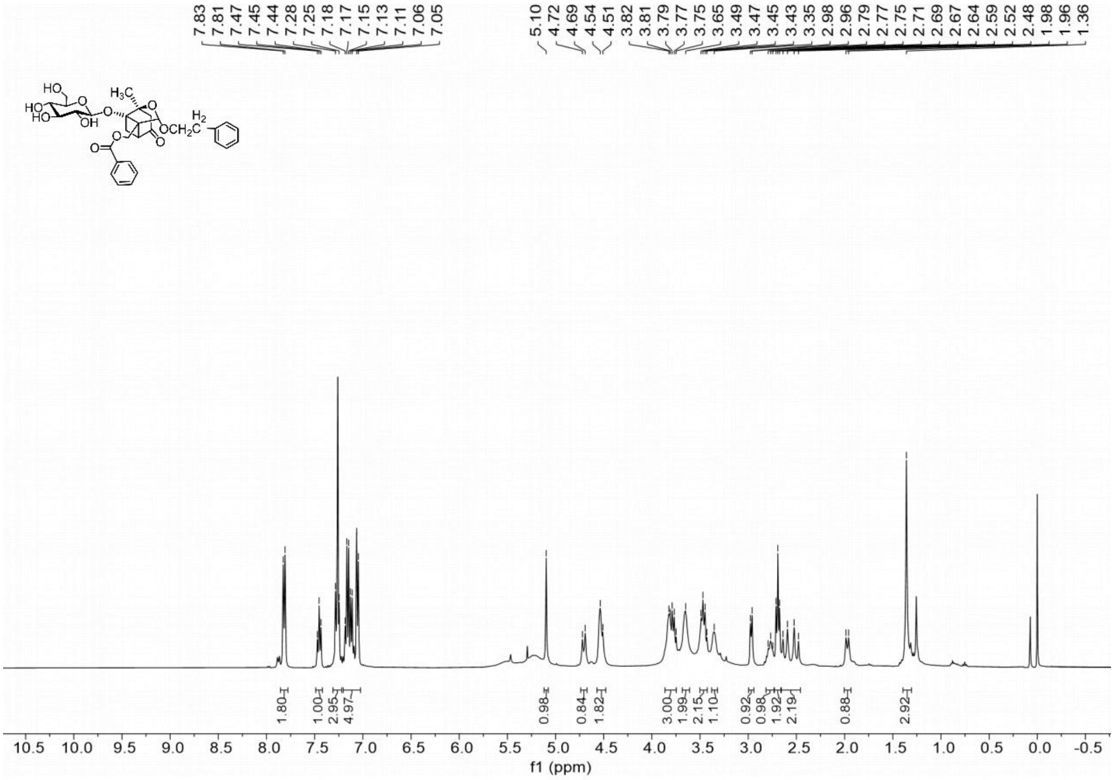

Figure S82. The ^{1}H NMR of compound **43**.

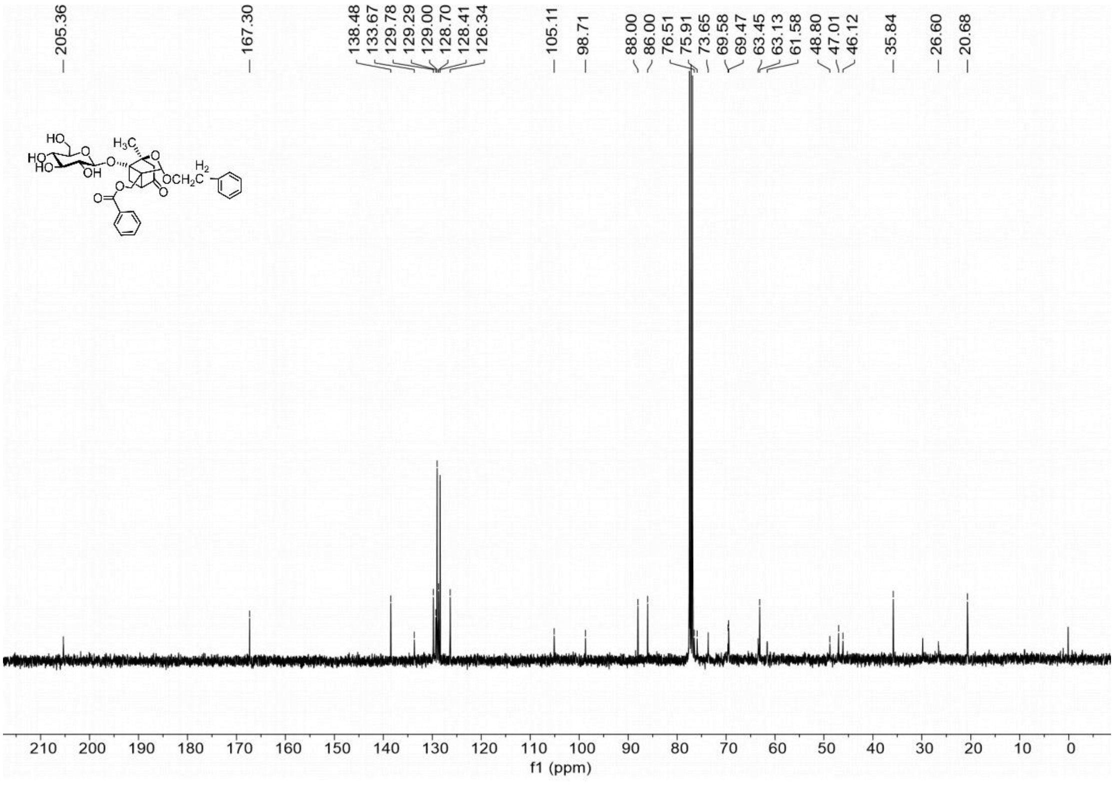

Figure S83. The ^{13}C NMR of compound **43**.

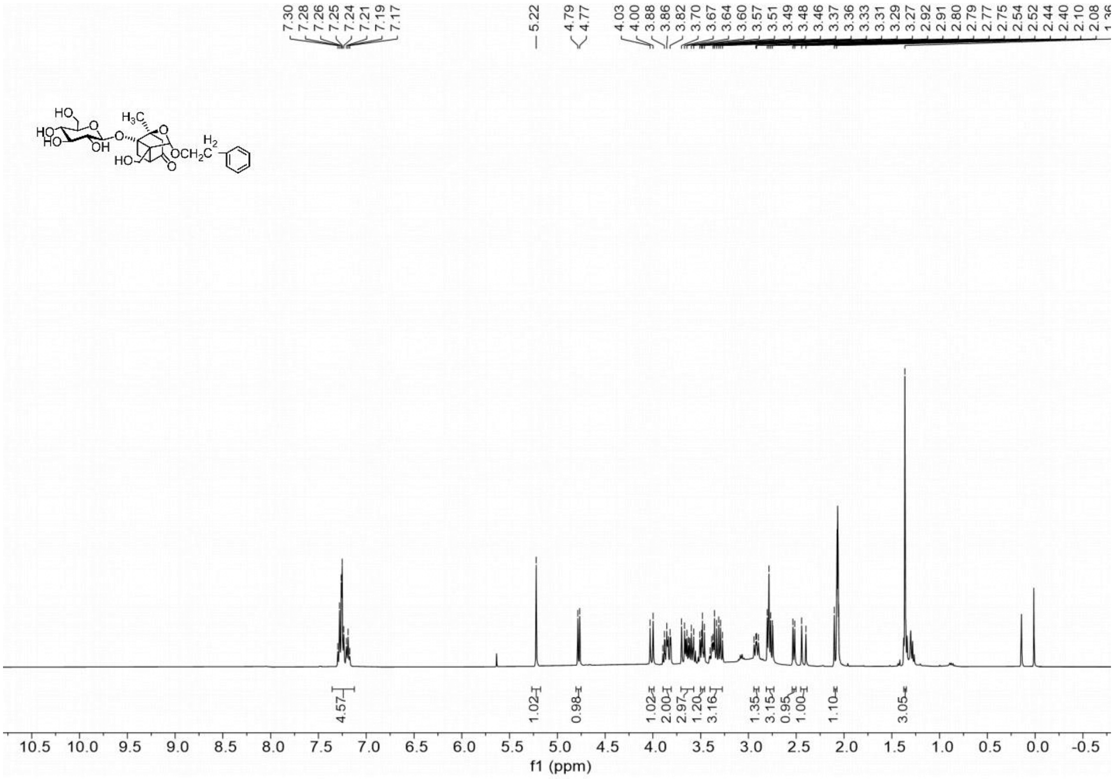

Figure S84. The ^{1}H NMR of compound **44**.

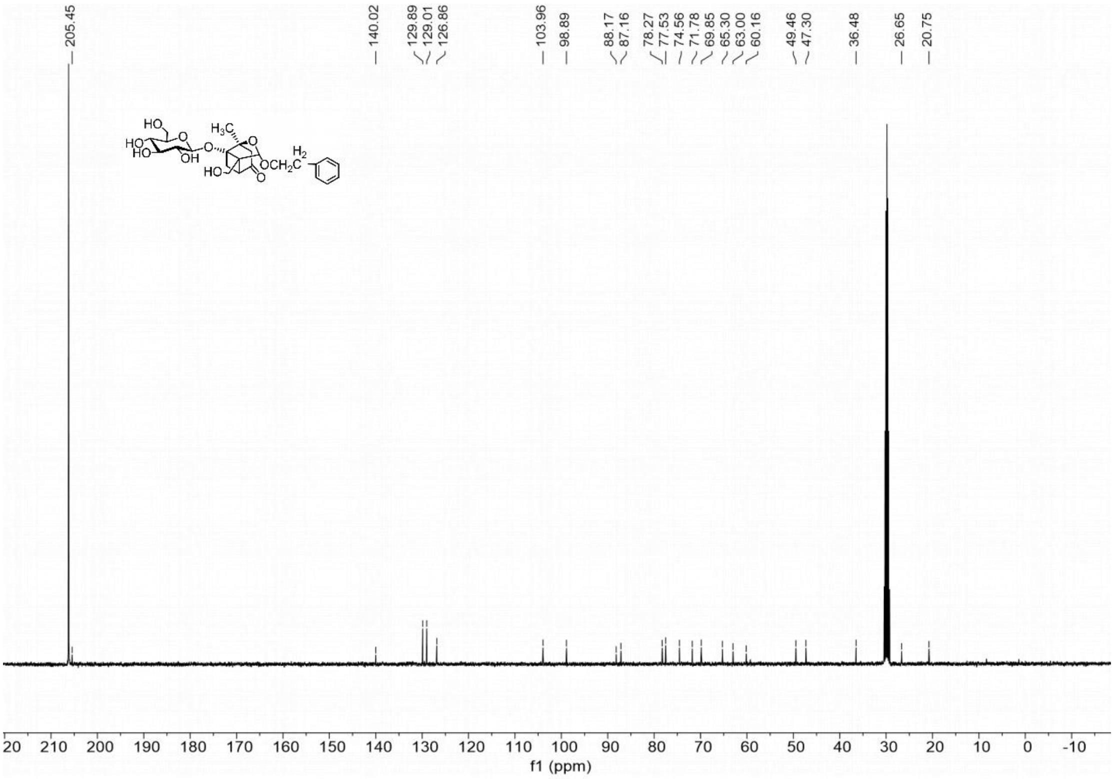

Figure S85. The ^{13}C NMR of compound **44**.

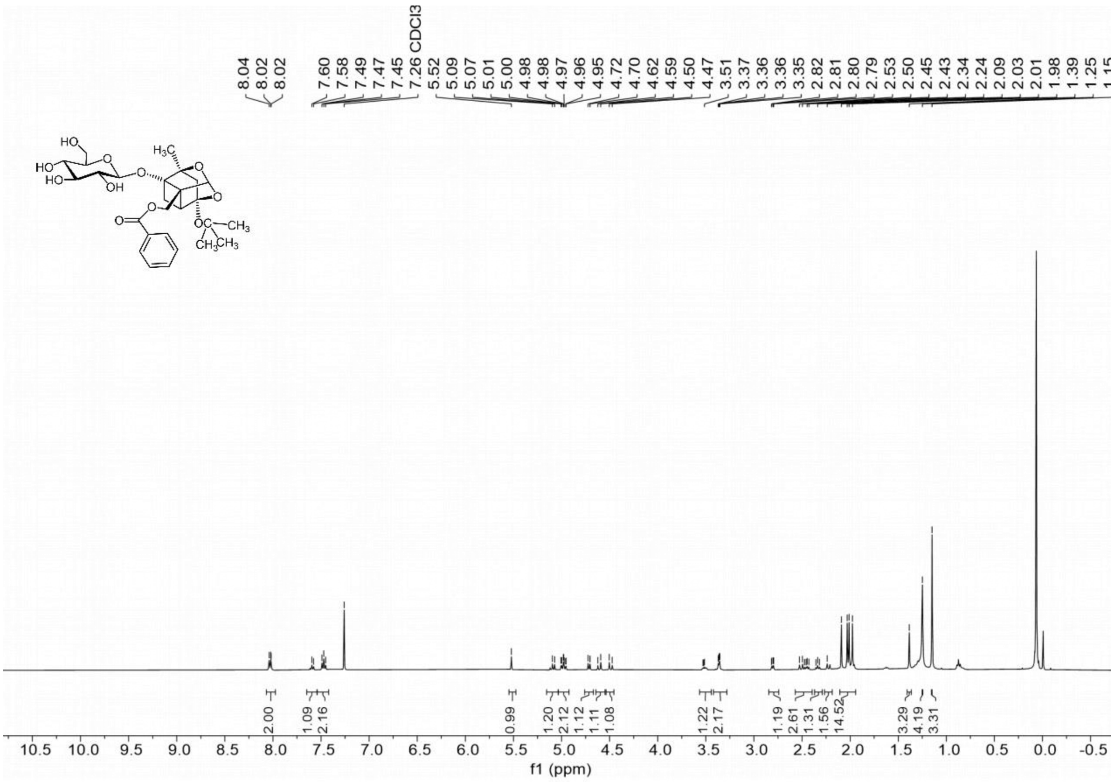

Figure S86. The ^{1}H NMR of compound **45**.

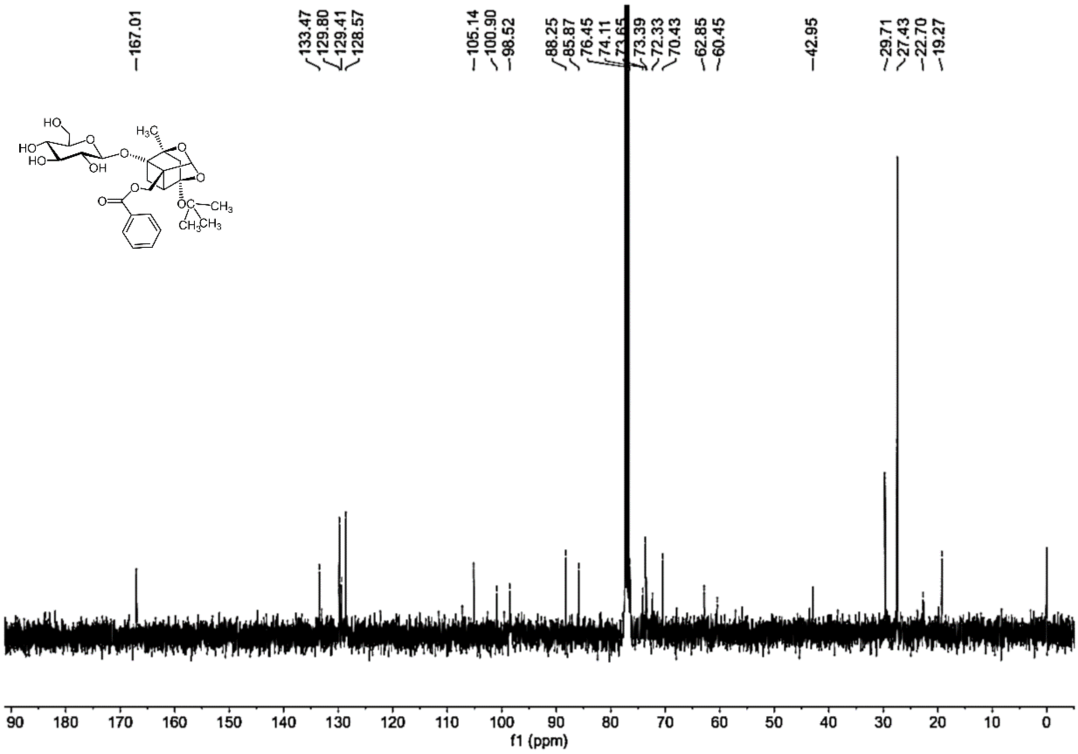

Figure S87. The ^{13}C NMR of compound **45**.

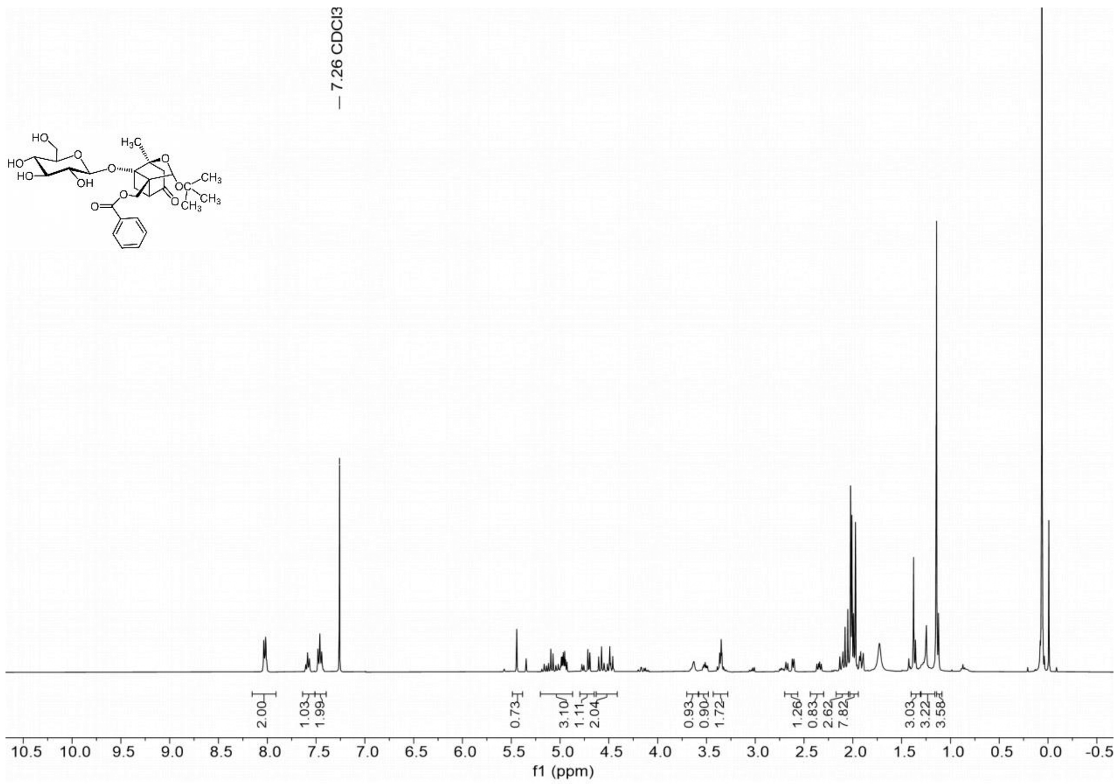

Figure S88. The ^{1}H NMR of compound **46**.

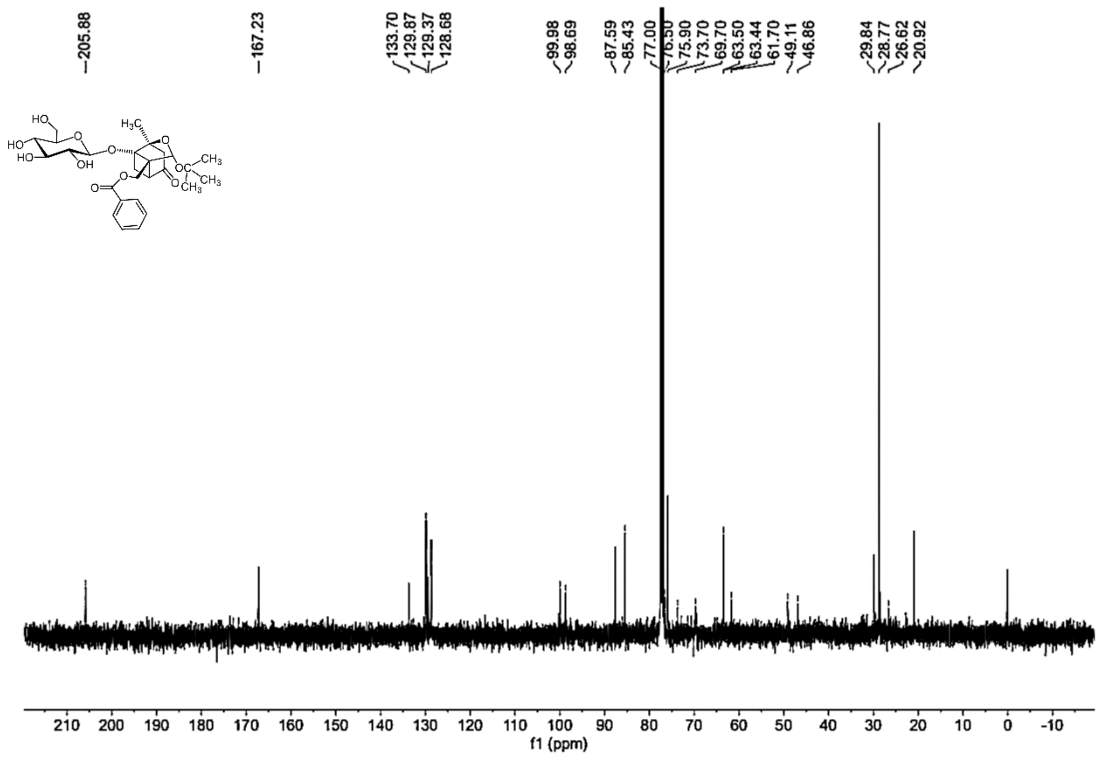

Figure S89. The ^{13}C NMR of compound **46**.

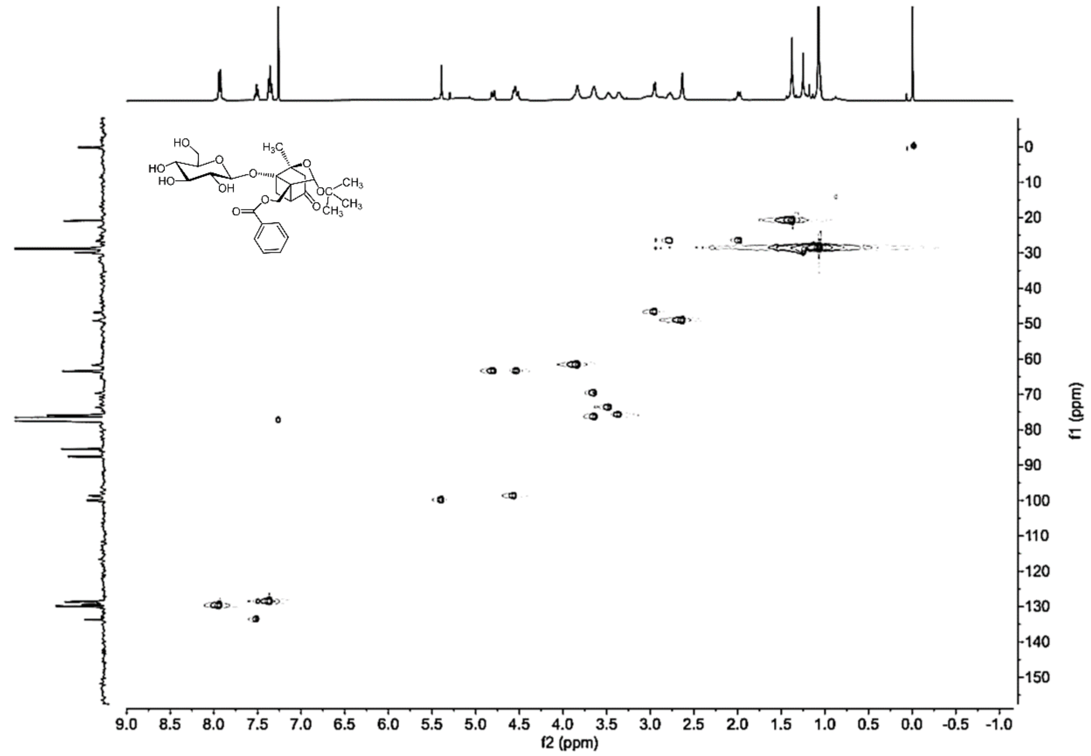

Figure S90. The HSQC-NMR of compound **46**.

3. Single crystal structure and data

CCDC 2249825 (Compound **11**), 2249824 (Compound **17**), and 2249823 (Compound **18**) containing the supplementary crystallographic data can be obtained free of charge from The Cambridge Crystallographic Data Centre via https://www.ccdc.cam.ac.uk/structures/search

Compounds single crystal data

**Table S3** Crystal data and structure refinement for compound **11**.	
Empirical formula	$C_{18}H_{28}O_{10}$
Formula weight	404.40
Temperature/K	293.15
Crystal system	orthorhombic
Space group	$P2_12_12_1$
a/Å	9.9334(8)
b/Å	13.2276(8)
c/Å	14.4439(14)
α/°	90
β/°	90
γ/°	90
Volume/Å^3	1897.9(3)
Z	4
ρ_{calc}g/cm^3	1.415
μ/mm^{-1}	0.116
F(000)	864.0
Crystal size/mm^3	0.35 × 0.3 × 0.25
Radiation	MoKα (λ = 0.71073)
2Θ range for data collection/°	6.16 to 52.732
Index ranges	-12 ≤ h ≤ 12, -16 ≤ k ≤ 11, -17 ≤ l ≤ 18
Reflections collected	5755
Independent reflections	3597 [R_{int} = 0.0249, R_{sigma} = 0.0589]
Data/restraints/parameters	3597/0/260
Goodness-of-fit on F^2	1.037
Final R indexes [I>=2σ (I)]	R_1 = 0.0673, wR_2 = 0.1612
Final R indexes [all data]	R_1 = 0.0919, wR_2 = 0.1791
Largest diff. peak/hole / e Å^{-3}	0.70/-0.29
Flack parameter	0.6(10)

**Table S4** Crystal data and structure refinement for compound **17**.

Empirical formula	$C_{34}H_{42}O_{15}$
Formula weight	690.67
Temperature/K	293.15
Crystal system	monoclinic
Space group	C2
a/Å	20.613(3)
b/Å	5.9223(6)
c/Å	29.982(3)
α/°	90
β/°	100.058(12)
γ/°	90
Volume/Å^3	3603.8(7)
Z	4
ρ_{calc}g/cm^3	1.273
μ/mm^{-1}	0.100
F(000)	1464.0
Crystal size/mm^3	0.35 × 0.1 × 0.05
Radiation	MoKα (λ = 0.71073)
2Θ range for data collection/°	6.234 to 52.744
Index ranges	-25 ≤ h ≤ 25, -7 ≤ k ≤ 4, -29 ≤ l ≤ 37
Reflections collected	7962
Independent reflections	5359 [R_{int} = 0.0214, R_{sigma} = 0.0581]
Data/restraints/parameters	5359/1/449
Goodness-of-fit on F^2	0.934
Final R indexes [I>=2σ (I)]	R_1 = 0.0693, wR_2 = 0.1838
Final R indexes [all data]	R_1 = 0.1128, wR_2 = 0.2076
Largest diff. peak/hole / e Å^{-3}	0.59/-0.26
Flack parameter	-1.2(8)

**Table S5** Crystal data and structure refinement for compound **18**.	
Empirical formula	$C_{34}H_{42}O_{15}$
Formula weight	690.67
Temperature/K	293.15
Crystal system	monoclinic
Space group	C2
a/Å	19.019(3)
b/Å	6.4924(8)
c/Å	29.815(3)
α/°	90

β/°	95.370(11)
γ/°	90
Volume/Å^3	3665.3(8)
Z	4
ρ_{calc}g/cm^3	1.252
μ/mm^{-1}	0.099
F(000)	1464.0
Crystal size/mm^3	0.35 × 0.3 × 0.25
Radiation	MoKα (λ = 0.71073)
2Θ range for data collection/°	6.634 to 52.742
Index ranges	-21 ≤ h ≤ 23, -8 ≤ k ≤ 8, -37 ≤ l ≤ 37
Reflections collected	14487
Independent reflections	7000 [R_{int} = 0.0238, R_{sigma} = 0.0387]
Data/restraints/parameters	7000/1/471
Goodness-of-fit on F^2	1.015
Final R indexes [I>=2σ (I)]	R_1 = 0.0572, wR_2 = 0.1347
Final R indexes [all data]	R_1 = 0.0916, wR_2 = 0.1577
Largest diff. peak/hole / e Å^{-3}	0.17/-0.17
Flack parameter	-0.9(5)
